# HIV Prevention in Care and Treatment Settings: Baseline Risk Behaviors among HIV Patients in Kenya, Namibia, and Tanzania

**DOI:** 10.1371/journal.pone.0057215

**Published:** 2013-02-25

**Authors:** Daniel P. Kidder, Pam Bachanas, Amy Medley, Sherri Pals, Harriet Nuwagaba-Biribonwoha, Marta Ackers, Andrea Howard, Nick DeLuca, Redempta Mbatia, Muhsin Sheriff, Gilly Arthur, Frieda Katuta, Peter Cherutich, Geoffrey Somi, for the PwP Evaluation Study team

**Affiliations:** 1 Division of Global HIV/AIDS, Center for Global Health, Centers for Disease Control and Prevention, Atlanta, Georgia, United States of America; 2 ICAP Columbia University, New York, New York, United States of America; 3 Centers for Disease Control and Prevention, Nairobi, Kenya; 4 Centers for Disease Control and Prevention, Windhoek, Namibia; 5 ICAP Columbia University, Dar es Salaam, Tanzania; 6 ICAP Columbia University, Nairobi, Kenya; 7 Centers for Disease Control and Prevention, Dar es Salaam, Tanzania; 8 Ministry of Health and Social Services, Windhoek, Namibia; 9 Ministry of Health, Nairobi, Kenya; 10 Ministry of Health, Dar es Salaam, Tanzania; University of Buea, Cameroon

## Abstract

HIV care and treatment settings provide an opportunity to reach people living with HIV/AIDS (PLHIV) with prevention messages and services. Population-based surveys in sub-Saharan Africa have identified HIV risk behaviors among PLHIV, yet data are limited regarding HIV risk behaviors of PLHIV in clinical care. This paper describes the baseline sociodemographic, HIV transmission risk behaviors, and clinical data of a study evaluating an HIV prevention intervention package for HIV care and treatment clinics in Africa. The study was a longitudinal group-randomized trial in 9 intervention clinics and 9 comparison clinics in Kenya, Namibia, and Tanzania (N = 3538). Baseline participants were mostly female, married, had less than a primary education, and were relatively recently diagnosed with HIV. Fifty-two percent of participants had a partner of negative or unknown status, 24% were not using condoms consistently, and 11% reported STI symptoms in the last 6 months. There were differences in demographic and HIV transmission risk variables by country, indicating the need to consider local context in designing studies and using caution when generalizing findings across African countries. Baseline data from this study indicate that participants were often engaging in HIV transmission risk behaviors, which supports the need for prevention with PLHIV (PwP).

**Trial Registration:**

ClinicalTrials.gov NCT01256463

## Introduction

As the global HIV epidemic enters its fourth decade, 34 million people are estimated to be living with HIV [1]. Since the beginning of the epidemic, HIV care and treatment efforts have rapidly expanded and 5.3 million people are now receiving anti-retroviral treatment (ART) [2]. The expansion of HIV treatment efforts has decreased the morbidity and mortality associated with HIV infection and people living with HIV (PLHIV) are now living longer [3]. Unfortunately, HIV incidence remains high with an estimated 2.6 million new infections in 2009 [2]. Traditional HIV prevention efforts have focused on reducing HIV risk among individuals who are HIV-negative or of unknown serostatus. Recently, however, there has been increasing recognition of the importance of addressing the prevention needs of PLHIV as part of a comprehensive and integrated HIV prevention, care, and treatment strategy [4]. In addition, studies have demonstrated the efficacy of providing ART as a mechanism to reduce the likelihood of HIV transmission [5], adding further momentum to the strategy of prevention with PLHIV (PwP).

Population-based surveys from a number of countries in sub-Saharan Africa highlight the need for effective prevention services for PLHIV. First, fewer than 40% of those who have HIV are aware of their status [2], and even fewer know their partner's HIV status. Moreover many PLHIV, especially women, find it difficult to disclose their HIV-positive status to their sexual partners [6], [7], [8]. However, many PLHIV are in serodiscordant relationships with estimates from eastern Africa indicating that 40 to 50% of married HIV-positive individuals have an HIV-negative spouse or partner [9], [10], [11]. Second, low rates of condom use in stable relationships may place the HIV-negative partner in serodiscordant partnerships at high risk of acquiring HIV [12]. Furthermore, alcohol use among some PLHIV has been found to be high and is associated with both increased risky sexual behavior [13], [14] and reduced adherence to antiretroviral medications (ARVs) [15]. PLHIV may also be co-infected with other sexually transmitted infections (STIs) which can be more severe and difficult to treat in HIV-positive persons, and incident STIs are an indicator of unprotected sex [16]. Another effective prevention service is preventing mother to child transmission (PMTCT) of HIV. Although PMTCT services are improving in sub-Saharan Africa, many HIV-positive women report an unmet need for contraception and safer pregnancy counseling [12], [17], and with access to PMTCT services often limited, access to contraception is even more critical.

Many PLHIV in these population studies are not aware of their status and may not be enrolled in HIV clinical care [10], [12], [13]. However, with the expansion of HIV care and treatment services, more PLHIV are now engaged in clinical care and have regular contact with health care providers. Yet data are limited regarding whether PLHIV in care are engaging in behaviors that put them at risk for acquiring other STIs or transmitting HIV to uninfected partners. In addition, studies that have been done among PLHIV in clinical settings have typically been small scale and conducted in a single country. This paper describes the baseline socioedemographic, HIV risk behaviors, and clinical data for a clinic-based HIV prevention intervention for PLHIV attending HIV care and treatment clinics in Kenya, Namibia, and Tanzania. Country differences in health and risk behaviors are also examined in order to explore how these factors differ across cultural contexts.

## Methods

The protocol for this trial and supporting CONSORT checklist are available as supporting information; see Checklist S1 and Protocol S1.

### Study design

The study was a longitudinal group-randomized trial, randomized at the clinic level. Six HIV care and treatment clinics in each of three countries (Kenya, Namibia, and Tanzania) participated, for a total of 18 clinics. The sites chosen were six district-level hospitals with HIV outpatient facilities on-site. Each clinic was matched to another clinic in the same country based on level of care and services provided (e.g., health care provider/patient ratio, number of HIV-positive patients enrolled in care), for a total of three pairs of clinics in each country. Intervention status was randomly assigned (by coin flip) to one clinic in each of the three matched pairs. The other clinic was assigned to a wait-list comparison condition. At the intervention clinics, health care providers (HCPs) and lay counselors (LCs) were trained to provide a package of HIV prevention messages and services (described below) as part of the routine care offered to all HIV-positive patients (not just study participants) during their clinic visits. In the comparison clinics, providers and staff delivered services following their usual standard of care. Following final study data collection, HCPs and LCs in the comparison clinics were trained to deliver the HIV prevention services and messages.

Patient participants were assessed at baseline, 6-months, and 12-months post-intervention. To allow sufficient time for patients to be exposed to the intervention during regular clinic visits, the 6-month follow-up data collection began 6 months after clinic staff were trained and the intervention was implemented. To make the intervention and comparison clinic project procedures as similar as possible, the 6 and 12-month follow-up assessments occurred during the same time periods in both the intervention and comparison clinics.

#### HIV Prevention Intervention

The intervention and materials developed for this project were adapted from Partnership for Health (PfH) [18], which is a brief, provider-delivered counseling program for PLHIV. PfH is designed to improve patient-provider communication about safer sex, disclosure of serostatus, and HIV prevention by incorporating counseling about safer sex and disclosure into the routine services provided to all PLHIV by health care providers during each clinic visit. It is based on a social cognitive model that uses message framing, repetition, and reinforcement to increase the patient's knowledge, skills, and motivations to practice safer sex. The investigators obtained input from health care providers in Kenya, Uganda, and Botswana for the adaptation of the materials for the African context and field tested the interventions in clinics in Kenya. The original intervention was expanded to include additional prevention messages (e.g., partner testing, alcohol reduction) and services (e.g., family planning, STI screening) to more comprehensively address prevention with PLHIV within the context of sub-Saharan Africa.

Following completion of the baseline data collection, HCPs (including physicians, clinical officers, nurses, pharmacists) in the intervention clinics were trained to incorporate HIV prevention services and messages into their routine clinical patient assessments and to provide these services and messages to all clinic patients (not just study participants) during each routine clinic visit. Specifically, when HCPs met with a patient individually in the exam room they incorporated HIV prevention services and messages into the routine care provided to the patients. The HIV prevention services included assessing and addressing sexual risk behaviors, partner HIV testing and knowledge of partner's status, medication adherence, alcohol use; identifying and treating STIs; and providing sufficient quantities of condoms. HCPs also addressed family planning needs. For those desiring pregnancy, guidance on safer conception, pregnancy, and delivery was provided to men and women. Participants who did not currently desire pregnancy were encouraged to use condoms plus another form of highly effective contraception. Hormonal contraceptives were provided within the HIV care and treatment clinics and women who were interested in long-acting methods were referred to other clinics to receive those methods. Health care providers were also trained to help patients set a prevention goal (e.g., increasing condom use).

Trained lay counselors provided group education sessions in clinic waiting areas on basic HIV/AIDS, HIV transmission to partners, mother-to-child transmission, treatment adherence, and healthy living. In addition, they provided individual counseling on the prevention issues raised by HCPs including disclosure, encouraging HIV testing of sex partner(s) and children, condom use (including condom use demonstrations), alcohol reduction, and medication adherence. Finally, counselors were trained to conduct partner and couples HIV testing and counseling (Namibia and Kenya). In Tanzania, lay counselors only provided pre- and post-test counseling, as they were not permitted by national guidelines to conduct HIV testing. Patients were either referred by HCPs to the LCs for additional counseling or the patients could choose on their own to visit the LCs.

#### Ethics

The study was reviewed and approved by Institutional Review Boards (IRBs) at all the collaborating institutions conducting the research. These included the US Centers for Disease Control and Prevention (CDC), Columbia University Medical Center, Kenya Medical Research Institute (KEMRI)/National Ethical Review Committee, Namibia Ministry of Health and Social Services (MOHSS), Tanzania National Institute of Medical Research (NIMR), and Zanzibar Medical Ethical Committee (ZAMEC).

Eligible participants were read an informed consent form and given the opportunity to ask questions before written consent was obtained. Participants consented to complete three questionnaires during the study period, allow collection of information from their medical charts, and provide contact information for participant tracking during the follow-up period. Participants were offered a drink and/or a small snack during or after the questionnaire administration.

### Data collection procedures

Each day, study staff approached every third patient in the clinic waiting area and asked if he/she was interested in participating in a study. Interested patients were taken to a private area, told about the project, and screened for eligibility. Inclusion criteria required that patient participants were 18 years of age or older, enrolled in the HIV care and treatment clinic (documented HIV infection) and had received care at the study clinic at least twice prior to study enrollment. Participants also had to report being sexually active within the past 3 months, and report planning to attend the clinic for at least 1 year. Women who knew they were pregnant and male partners of pregnant women were ineligible for study enrollment, as family planning counseling and pregnancy were study outcomes. Respondents who were aware of their partners already participating in the study were excluded. All patient interviews were available in English, as well as Kiswahili (in Kenya and Tanzania) and Oshiwambo and Afrikaans (in Namibia).

In order to have sufficient representation of patient participants by gender and ART status, each clinic attempted to enroll equal numbers of men and women, as well as equal numbers of ART and pre-ART patients (care only). Treatment status was assessed on the screening form to track the number of participants enrolled in each group.

The patient baseline questionnaire took approximately one hour to complete and was administered by a trained project interviewer who was not part of the clinic staff. Data collection start dates were staggered across countries, with each baseline data collection period taking approximately 3 months. Baseline data collection began in October 2009 and was completed in all countries by April 2010. Data presented in this paper were collected from patient medical chart review and patient questionnaires.

#### Medical Chart Review

Data extracted from patients' medical charts included date of HIV diagnosis, dates of HIV clinic visits in the past 6 months, clinical indicators of HIV (e.g., most recent CD4 count, WHO clinical stage), prescribed medications, pregnancy status, contraceptive use, STI symptoms and STI treatment provided, and meetings with lay counselors. In addition, data on dates and amount of medication dispensed to patients in the past 6 months were collected from pharmacy records.

#### Participant Questionnaire

The patient questionnaire collected information regarding sociodemographics, HIV testing and care, mental and physical health, social support, alcohol use, HIV medications and adherence, disclosure of serostatus, knowledge of HIV/AIDS, sexual behavior, sexual self-efficacy, history of violence, STIs, fertility desire, family planning, and prevention services received. As much as possible, measures included in the questionnaire had been used previously in studies conducted in Africa.

To assess adherence participants were asked to name their HIV medications, number of pills taken, dose (number of times per day), and whether they had missed any doses in the last 30 days [19], [20]. For sexual behavior, participants could report up to five partners with whom they had sex in the past 3 months and identified whether each partner was a spouse, main partner, or non-main partner. Disclosure of the participant's HIV status to each sex partner(s) was assessed. For this analysis, disclosure of HIV status was to their spouse or most recent main partner. If they didn't report a spouse or main partner, disclosure to a non-main partner was used. Participants also indicated whether each partner had been tested for HIV and if so, whether the participant knew that partner's HIV status.

The 10-item World Health Organization's Alcohol Use Disorders Identification Test (AUDIT) [21] was used to measure alcohol use. Based on the AUDIT scoring criteria, participants were categorized as non-drinker (0), non-problem drinker (<8), problem drinker (≥8), and likely dependent on alcohol (≥13 for women, ≥15 for men). STI symptoms were based on patients' report of whether they had experienced discharge from the penis or vagina, sores in the genital area, or (for female patients only) abdominal pain.

### Data analysis

The SAS GLIMMIX procedure was used to test for differences between countries (Table 1) and between males and females for categorical variables (SAS PROC MIXED was used for continuous variables). When testing for country differences, clinic was entered as a random effect to account for the correlation of observations within clinic, country was entered as a categorical independent variable, and the variable of interest was entered as the dependent variable. A multinomial distribution with a generalized logit link was assumed for the dependent variable. For gender differences, gender was the independent variable and the variable of interest was the dependent variable. Clinic was again entered as a random effect and the appropriate distribution for the dependent variable used (binomial, multinomial or continuous). All data analyses were considered significant at p<0.05.

**Table 1:** Sociodemographic and health characteristics of baseline PwP evaluation study participants.

	Overall (n = 3538)	Kenya (n = 1156)	Namibia (n = 1186)	Tanzania (n = 1196)	Country Differences*
	n (%)	n (%)	n (%)	n (%)	p-value
**Gender**					0.5139
Male	1483 (41.9)	490 (42.4)	514 (43.4)	479 (40.1)	
Female	2054 (58.1)	665 (57.6)	672 (56.7)	717 (60.0)	
**Age**					0.0045
18–29	616 (17.4)	229 (19.8)	215 (18.1)	172 (14.4)	
30–39	1667 (47.1)	545 (47.2)	585 (49.3)	537 (44.9)	
40–49	980 (27.7)	303 (26.2)	306 (25.8)	371 (31.0)	
≥50	274 (7.8)	78 (6.8)	80 (6.8)	116 (9.7)	
**Education**					<0.0001
No school	340 (9.6)	27 (2.3)	95 (8.0)	218 (18.3)	
Primary	1890 (53.5)	665 (57.6)	403 (34.0)	822 (69.0)	
Secondary	1214 (34.4)	394 (34.1)	678 (57.2)	142 (11.9)	
More than secondary	88 (2.5)	69 (6.0)	9 (0.8)	10 (0.8)	
**Marital status**					<0.0001
Married/living together	2170 (61.4)	822 (71.2)	542 (45.7)	806 (67.5)	
Single, never married	781 (22.1)	95 (8.2)	608 (51.3)	78 (6.5)	
Separated/divorced	359 (10.2)	156 (13.5)	10 (0.8)	193 (16.2)	
Widowed	225 (6.4)	82 (7.1)	26 (2.2)	117 (9.8)	
**Number of wives (married/cohabiting males only)**					0.2266
1	972 (94.8)	381 (97.0)	271 (93.5)	320 (93.6)	
2	53 (5.2)	12 (3.1)	19 (6.6)	22 (6.4)	
**Number of wives/partners husband lives with (married females only)**					0.1064
1	943 (86.8)	389 (91.8)	187 (87.0)	367 (81.9)	
2	123 (11.3)	31 (7.3)	21 (9.8)	71 (15.9)	
3–5	21 (1.9)	4 (0.9)	7 (3.3)	10 (2.2)	
**Number of people in household**					<0.0001
1–2	719 (20.4)	313 (27.1)	193 (16.4)	213 (17.9)	
3–4	1218 (34.6)	513 (44.5)	312 (26.5)	393 (33.0)	
5–6	826 (23.4)	258 (22.4)	244 (20.7)	324 (27.2)	
≥7	761 (21.6)	70 (6.1)	429 (36.4)	262 (22.0)	
**Have children** (% yes)	3196 (90.4)	1084 (93.9)	1059 (89.3)	1053 (88.0)	<0.0001
**Number of living children (of those with children)**					0.0218
0–1	762 (23.9)	268 (24.7)	254 (24.1)	240 (22.8)	
2	868 (27.2)	327 (30.2)	283 (26.8)	258 (24.5)	
3	659 (20.6)	234 (21.6)	209 (19.8)	216 (20.5)	
≥4	904 (28.3)	255 (23.5)	310 (29.4)	339 (32.2)	
**Religion**					<0.0001
Roman Catholic	728 (20.6)	311 (27.0)	236 (19.9)	181 (15.1)	
Evangelical/Pentecostal	954 (27.0)	251 (21.8)	670 (56.6)	33 (2.8)	
Islamic/Muslim	912 (25.8)	9 (0.8)	0 (0.0)	903 (75.5)	
Protestant	477 (13.5)	428 (37.1)	7 (0.6)	42 (3.5)	
Other	463 (13.1)	155 (13.4)	271 (22.9)	37 (3.1)	
**Paid work last 6 mos.** (% yes)	1565 (44.3)	744 (64.4)	555 (46.9)	266 (22.3)	<0.0001
**Paid work last 7 days** (% yes, of those who worked in past 6 mos.)	1307 (83.3)	651 (87.5)	452 (81.2)	204 (76.1)	0.0589
**Monthly household income** b					0.0079
<30 USD	929 (30.8)	225 (20.6)	301 (27.6)	403 (48.4)	
30–99	1131 (37.5)	524 (48.0)	328 (30.1)	279 (33.5)	
≥100	954 (31.7)	344 (31.5)	460 (42.2)	150 (18.0)	
**Years since HIV diagnosis**					<0.0001
<1 years	925 (26.2)	331 (28.7)	189 (16.0)	405 (33.9	
1 to <2 years	806 (22.8)	283 (24.5)	200 (16.9)	323 (27.0)	
2 to <3 years	723 (20.5)	250 (21.7)	218 (18.4)	255 (21.3)	
≥3 years	1081 (30.6)	291 (25.2)	577 (48.7)	213 (17.8)	
**Most recent CD4**					0.4564
<200	828 (23.7)	245 (21.3)	304 (26.1)	279 (23.6)	
200–349	1036 (29.6)	338 (29.3)	341 (29.3)	357 (30.2)	
350–500	797 (22.8)	278 (24.1)	269 (23.1)	250 (21.2)	
≥501	838 (24.0)	291 (25.3)	252 (21.6)	295 (25.0)	

Notes. Percentages may not total to 100% due to rounding. The number of participants for individual variables may not sum to overall totals due to non-response from some participants.

* F-statistics used to test for country differences.

a One Muslim observation was added per site in Namibia (6 total) to allow the model to converge.

b Local currency was converted to US dollars (USD) for comparability.

## Results

### Participants

**Figure 1:**
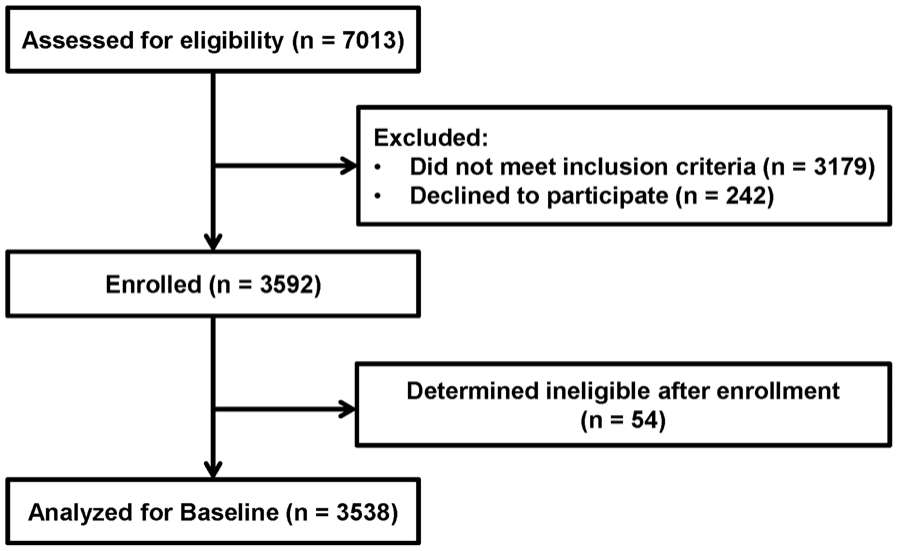
Flow diagram of patient participants in a group-randomized trial of prevention with people living with HIV in HIV care and treatment clinics.

A total of 3,538 eligible patient participants were enrolled and completed the baseline questionnaire between September 2009 and April 2010 (see Figure 1 for patient flow diagram). Despite intensive recruitment efforts, all countries had difficulty enrolling male patients, especially those who were receiving care only and not taking ARVs. Given the challenges enrolling men, the recruitment goals for each clinic were revised from an even 50 participants in the male/female and on ARV/not on ARV groups to 60 males and 60 females on ARVs, 50 females in care only, and 30 men in care only. Even with these revised targets, one clinic had difficulty recruiting men who were in care only.

### Sociodemographics

The majority of participants (58%) were female (Table 1). Nearly half of participants were between 30 and 39 years old; however, women were younger (M = 35, SD = 7) compared to men (M = 41, SD = 9, F = 548, p<.0001). Nearly two-thirds of participants either attended school through the primary grades (54%) or had not attended school (10%). Sixty one percent of study participants reported being married or living together as married. More men (69%) than women (56%) reported being married/living together as married (F = 29.55, p<0.0001). Only 5% of married men reported 2 wives or partners they lived with as married, although 13% of married women reported that their husband had more than 1 wife or partner that he lived with as married. Ninety percent of participants had children who were still living and 51% had 2 or fewer living children. There were country differences in sociodemographics which are shown in Table 1.

Because there were no Islamic/Muslim participants in Namibia, one Muslim observation was added per site in Namibia (6 total) to allow the model to converge. There were significant differences in religion (F = 52.03, p<0.0001; Table 1). Tanzania had 75% Islamic/Muslim participants, whereas Kenya was more evenly split among the different religions, with the largest group being Protestant (37%). Namibian participants were mostly Evangelical/Pentecostal (56%).

Less than half of participants (44%) had done paid work in the last 6 months. Among those who had worked in the last 6 months, 83% had also worked in the past 7 days. Men were more likely to have reported paid work in the last 6 months (55%) than women (37%; F = 126.24, p<0.0001) and in the last 7 days (male = 86%, female = 81%; F = 5.31, p = 0.02). Monthly household income was converted from local currency to US dollars for comparability across countries, with 68% of participants earning less than $100 per month.

### Health Status and Medication Adherence

About a quarter (26%) of participants were diagnosed with HIV in the previous year, and more than two-thirds (70%) had been diagnosed with HIV within the past 3 years. In Namibia, nearly half of participants had been diagnosed for over 3 years, compared with only 25% of Kenyan and 18% of Tanzanian participants (p<0.0001). Nearly a quarter of all participant's most recent CD4 count was less than 200 cells/mm^3^, and another 30% had CD4 counts between 200 and 349. ART patients had a median CD4 count of 287 cells/mm^3^ (median time since CD4 test = 137 days) and the median CD4 count for those not on ART was 413 (median time since test = 83 days). Among participants who had been on ARVs less than one year, the median CD4 was 190 cells/mm^3^. Although there were no significant gender differences in time since HIV diagnosis, CD4 count was significantly higher for women compared to men (422 vs. 332, p<0.0001).

Nearly all participants (88%) were taking HIV medications (ARVs and cotrimoxazole), and 83% of those taking medications reported that they had not missed any doses in the last 30 days (Table 2). There was no significant difference in adherence between those taking cotrimoxazole only compared with those on both ARVs and cotrimoxazole. When limited to the 64% of participants taking ARVs, adherence rates were similar with 81% reporting that they had not missed a dose in the past 30 days.

**Table 2:** Participant baseline HIV risk behaviors by country.

	Overall (n = 3538)	Kenya (n = 1156)	Namibia (n = 1186)	Tanzania (n = 1196)	Country Differences*
	n (%)	n (%)	n (%)	n (%)	p-value
**Disclosed HIV status to partner**					0.99
No	640 (18.2)	216 (18.7)	120 (10.2)	304 (25.5)	
Yes	2879 (81.8)	937 (81.3)	1053 (89.8)	889 (74.5)	
**Partner's HIV status**					<0.0001
HIV+	1675 (47.6)	548 (47.5)	681 (58.3)	446 (37.3)	
HIV−	666 (18.9)	217 (18.8)	179 (15.3)	270 (22.6)	
Unknown	1177 (33.5)	389 (33.7)	309 (26.4)	479 (40.1)	
**Consistent condom use, past 3 months** a					<0.0001
No	765 (23.7)	269 (24.2)	137 (14.2)	359 (31.1)	
Yes	2466 (76.3)	842 (75.8)	828 (85.8)	796 (68.9)	
**Alcohol use (AUDIT)**					<0.0001
Non-drinker	2826 (80.1)	985 (85.4)	813 (69.0)	1028 (86.0)	
Non-problem drinker	518 (14.7)	101 (8.8)	275 (23.3)	142 (11.9)	
Harmful drinker	113 (3.2)	34 (3.0)	61 (5.2)	18 (1.5)	
Likely dependent	71 (2.0)	34 (3.0)	29 (2.5)	8 (0.7)	
**Taking HIV medications** b					<0.0001
No	420 (11.9)	2 (0.2)	351 (29.6)	67 (5.6)	
Yes	3116 (88.1)	1153 (99.8)	835 (70.4)	1128 (94.4)	
**Missed dose of HIV medication,** b **past 30 days**					0.71
No	2574 (82.6)	975 (84.6)	686 (82.2)	913 (80.9)	
Yes	542 (17.4)	178 (15.4)	149 (17.8)	215 (19.1)	
**Any STI symptoms, ever**					0.005
No	2258 (63.8)	605 (52.4)	800 (67.5)	853 (71.3)	
Yes	1279 (36.2)	550 (47.6)	386 (32.6)	343 (28.7)	
**Any STI symptoms, past 6 months**					0.88
No	3135 (88.7)	1013 (87.7)	1062 (89.7)	1060 (88.8)	
Yes	398 (11.3)	142 (12.3)	122 (10.3)	134 (11.2)	
**Desire pregnancy or partner pregnancy in next 6 months**					<0.0001
No	2870 (83.5)	998 (88.9)	960 (82.9)	912 (78.9)	
Yes	567 (16.5)	125 (11.1)	198 (17.1)	244 (21.1)	
**Use highly effective family planning method** c					0.04
No	1837 (65.0)	605 (60.8)	550 (59.5)	682 (75.1)	
Yes	991 (35.0)	390 (39.2)	375 (40.5)	226 (24.9)	

Notes. Percentages may not total to 100% due to rounding. The number of participants for individual variables may not sum to overall totals due to non-response from some participants.

* F-statistics used to test for country differences.

a Used a condom at every sexual encounter.

b Includes antiretroviral medications and cotrimoxazole.

c Among those not desiring pregnancy or partner pregnancy (males). Family planning methods include pill, injectable, IUD, implant, male and female sterilization.

### HIV Transmission Risk Behaviors

Most participants (82%) reported that they had disclosed their HIV status to their partner (Table 2). Two-thirds of participants reported that they knew their partner's HIV status, with 48% reporting their partner was HIV-positive, 19% reporting their partner was HIV-negative, and 34% of participants did not know their partner's HIV status.

Self-reported consistent condom use rates were high with 76% of participants reporting that they had used a condom at every sexual encounter in the past three months (Table 2). Men were more likely to report consistent condom use than women (81% vs. 73%, p<0.0001). There were also country differences (p<0.0001), with more participants in Namibia reporting consistent condom use (86%) compared with Kenya (76%) and Tanzania (69%).

Most participants (80%) reported no alcohol use, 15% were non-problem drinkers, 3% harmful drinkers, and 2% scored as likely dependent on alcohol (Table 2). Fewer men reported no alcohol use and they were more likely than women to be classified as non-problem or harmful drinkers. There were also significant country differences (p<0.0001).

Over one-third of participants (36%) reported ever experiencing STI symptoms (Table 2), with significant country differences (p = 0.005), but no significant gender differences. Eleven percent of participants reported STI symptoms in the past six months, with more women (14%) reporting symptoms than men (7%; F = 46.52, p<.0001).

Female participants were asked whether they desired a pregnancy and male participants were asked whether they desired their spouse or main partner to be pregnant in the next 6 months. Only 17% of participants desired pregnancy (Table 2), although more men reported desiring their partners to be pregnant (20%) than women reported desiring a pregnancy (14%; p<.0001). There were also country differences, with Tanzanian participants more likely to desire pregnancy (21%), followed by 17% of Namibians and 11% of Kenyan participants (p<.0001).

Among female participants who did not desire pregnancy and males who did not desire their partner to be pregnant, only 35% were using a highly effective contraceptive method (e.g., pills, injectable, IUD, implant, male or female sterilization). There were significant country differences in highly effective contraceptive use (p = .04); participants in Tanzania reported lower rates of contraceptive use (25%) than participants in either Kenya (39%) or Namibia (41%).

## Discussion

The group-randomized trial described in this paper is designed to evaluate the effectiveness and feasibility of integrating HIV prevention into the routine care of PLHIV attending care and treatment clinics in Kenya, Namibia, and Tanzania. Baseline data from the study participants provides information about the sociodemographic characteristics, health status, and HIV risk behavior of patients attending care. These data indicate that participants were similar in socioeconomic status (SES) to nationally representative samples in each of the countries [10], [22], [23], with low education and income levels. The lack of paid work and low income reported by many of the PLHIV in this study may affect their health and clinic attendance. For example, transportation costs are often reported as a reason for poor clinic attendance among PLHIV [24], [25], [26].

The mean age of participants in this sample was higher than national estimates of ages of PLHIV, especially for women [10], [22], [23], [27]. In addition, the majority of participants in this study were diagnosed with HIV within the past 3 years. Over half of participants had their most recent CD4 count less than 350 cells/mm^3^, and participants who had been on ARVs less than one year (20% of those on ARVs) had very low CD4 counts (median = 170). These data suggest that many participants accessed care late in their illness, a finding consistent with other studies from sub-Saharan Africa showing that many patients access care after developing advanced symptomatic disease [28], [29], [30], [31] and aren't benefiting from care and treatment interventions to improve morbidity and mortality. In addition, their declining CD4 counts and increased viral load are also placing their sexual partners at increased risk of HIV acquisition [32], [33], [34], [35], [36]. Increased HIV testing and counseling (HTC) efforts with focused linkage to care and treatment services in both facilities (e.g., ANC, TB, STI, and out-patient departments) and communities (e.g., home-based testing and mobile testing) are needed to identify individuals with asymptomatic disease, especially men, and ensure earlier enrollment into appropriate prevention, care, and treatment services. In addition, as one-third of participants did not know their partners' HIV status, more intensive efforts are clearly needed to increase partner and couple testing to identify HIV-positive persons and discordant couples and to link them with prevention, care, and treatment services.

Men in HIV care and treatment clinics and in the study sample are under-represented compared to the population of PLHIV. Men also had significantly lower CD4 counts than women. Nearly two-thirds of men had CD4 counts less than 350 cells/mm^3^ compared to less than half of women (64% vs. 46%, p<0.0001). This is consistent with several other studies from sub-Saharan Africa which have found that men are less likely than women to enroll in care at an earlier clinical stage [37], [38], [39], and to remain in clinical care after enrollment [40], [41], [42]. These findings highlight the importance of strengthening HIV testing and counseling programs targeting men, such as medical male circumcision programs and partner testing in ANC settings. Efforts to identify HIV-positive men earlier, link them to prevention, care and treatment services, and retain them in care are clearly needed.

The risk behavior data from this study identify HIV prevention needs among PLHIV attending HIV clinics. Specifically, nearly one-fifth of the participants were in a known discordant relationship, and over one-third did not know their partner's HIV status. In addition, 18% of the sample had not disclosed their HIV status to their partner and 24% were not consistently using condoms. These data indicate that many participants are in need of prevention services such as support for disclosure of HIV status, partner testing, and reducing unprotected sex. In addition, alcohol use was reported by about 20% of participants. Alcohol use has been associated with poor adherence [43], [44], [45] and increased disease progression [46], [47], [48], as well as increased risky sexual behavior among PLHIV [49]. Interventions to address these prevention issues in the context of comprehensive care and treatment services are indicated.

There were significant differences between countries in many of the reported HIV risk behaviors. For example, consistent condom use among PLHIV in care was 86% in Namibia, but significantly lower in Tanzania (69%). Similarly, only 60% of participants in Tanzania and 63% in Kenya knew their partner's HIV status, but 73% of Namibian participants knew their partner's status. There were differences in reported alcohol use as well, with Tanzanian and Kenyan participants less likely to drink alcohol than Namibian participants, and Tanzanian participants also less likely to report problem drinking. These participant baseline differences between countries highlight the importance of considering the local context when designing multi-country studies and the need to recognize that regional differences could affect generalizability of results from studies conducted in a single region or country.

This study has several limitations. Given that this was a clinic-based sample conducted in three countries with generalized HIV epidemics, the generalizability of these findings to PLHIV not enrolled in clinical care and/or to PLHIV in non-generalized epidemics may be limited. In addition, much of the data in this paper are from patient self-report, thus social desirability and recall bias may have affected participants' responses. As a result, some variables may be overestimated (e.g., condom use, disclosure, adherence) and others underestimated (e.g., alcohol use).

In summary, the baseline data from this study suggest that increased efforts are needed to identify PLHIV earlier, especially men, and to ensure they access prevention, care, and treatment services following diagnosis. Many PLHIV would also benefit from prevention interventions which address disclosure support, partner testing, alcohol and sexual risk reduction, as well as providing contraceptives and support for safer pregnancy. Integrating these services into the clinical care of HIV-positive persons may increase access to prevention interventions and improve retention in clinical care [4].

## Supporting Information

Checklist S1 **CONSORT Checklist.**
(DOCX)

Protocol S1 **Trial Protocol.**
(DOC)
