# Supplementary material for: HIV Prevention in Care and Treatment Settings: Baseline Risk Behaviors among HIV Patients in Kenya, Namibia, and Tanzania
Source: PLoS One. 2013 Feb 25;8(2):e57215. doi: 10.1371/journal.pone.0057215 (PMC3581447; doi:10.1371/journal.pone.0057215)
Supplement: Protocol S1 — Trial Protocol. (DOC) [file pone.0057215.s002.doc]

**INSTITUTIONAL REVIEW BOARD APPLICATION**

**HIV Prevention for People Living with HIV/AIDS: Evaluation of an Intervention Toolkit for HIV Care and Treatment Settings**

**Table of Contents**

**Project Overview 5**

Protocol Summary 5

Acronyms 6

Project Investigators and Roles 7

**Introduction 8**

Literature Review and Background 8

Study Justification 11

Intended use of study findings 11

Study design and locations 12

Objectives 13

Hypotheses 13

General Approach 14

**Procedures/Methods: Design 14**

How Study Design Addresses Research Questions and Meets Objectives 14

Audience Stakeholder Participation 14

Cost Benefit/Prevention Effectiveness 15

Study Timeline 15

**Procedures/Methods: Study Population 16**

Descriptions and Source of Study Population and Catchment Area 16

Case Definitions 16

Participant Inclusion Criteria 16

Participant Exclusion Criteria 16

Justification of Exclusion of Any Sub-segment of the Population 17

Estimated Number of Participants 17

Sampling, Including Sample Size and Statistical Power 17

Enrollment 19

Consent Process 20

**Procedures/Methods: Variables/Interventions 21**

Variables 21

Study Instruments 22

HIV Prevention Interventions 26

Outcomes and Minimum Meaningful Differences 32

**TABLE OF CONTENTS** (continued)

Training for All Study Personnel 33

**Procedures/Methods: Data Handling and Analysis 35**

Data Analysis Plan 35

Data Collection 38

Information Management and Analysis Software 41

Data Entry, Editing, and Management 41

Quality Control / Assurance 43

Bias in Data Collection, Measurement, and Analysis 43

Intermediate Reviews and Analysis 43

Limitations of Study 43

**Procedures/Methods: Handling of Unexpected or Adverse Events 44**

Response to New / Unexpected Findings, Changes in Study Environment 44

Identifying, Managing and Reporting of Adverse Events 44

Emergency Care 44

**Procedures/Methods: Dissemination, Notification, and Reporting of Results 44**

Notifying Participants of their Individual Results 44

Notifying Participants of Study Findings 45

Anticipated Products/Inventions Resulting from the Study and their Use 45

Disseminating Results to the Public 45

Publications Committee 45

**References 46**

**TABLE OF CONTENTS** (continued)

**APPENDICES**

Appendix A. Consent Form: Pilot Study 53

Appendix B. Pilot Study Questionnaire 55

Appendix C. Project Eligibility Screening Form 56

Appendix D. Consent Form: Patient 60

Appendix E. Consent Form: Health Care Provider and Lay Counselor 63

Appendix F. Consent Form: Patient for Observation of Clinic Visit 66

Appendix G. Contact Information Form 68

Appendix H. Patient Questionnaire 74

Appendix I. Clinical Care Survey: Health care provider 123

Appendix J. Integration of Prevention into Care and Treatment: Health care provider 127

Appendix K. Integration of Prevention into Care and Treatment: Lay counselor 133

Appendix L. Lay Counselor Record 141

Appendix M. Health Care Provider Observation Form 143

Appendix N. Lay Counselor Observation Form 146

Appendix O. Clinic Services Form 149

Appendix P. Patient Medical Chart Review Form 153

Appendix Q. Incident Report Form (1254) 160

**PROJECT OVERVIEW**

**Protocol Summary**

Each year, approximately 2.5 million people worldwide become newly infected with HIV, a trend expected to continue unless effective prevention interventions are rapidly brought to scale in the areas most affected by HIV (UNAIDS, 2006). The rapid scale-up of HIV care and treatment in resource-limited settings has provided the opportunity to reach many HIV-positive individuals with prevention messages and interventions in care and treatment settings. However, HIV prevention is rarely incorporated into the routine care and treatment of people living with HIV/AIDS, leaving missed opportunities to reach large numbers of patients with critical interventions.

The President’s Emergency Plan for AIDS Relief (PEPFAR) is supporting the Centers for Disease Control and Prevention’s (CDC) Global AIDS Program (GAP) Prevention Branch to develop and evaluate an HIV prevention intervention package for health care settings in sub-Saharan Africa. The HIV prevention intervention will be delivered to HIV-seropositive patients in HIV care and treatment clinics during all routine visits. Health care providers (including physicians, clinical officers, and nurses) will deliver HIV prevention messages on correct and consistent condom use, disclosure of serostatus, partner HIV testing, adherence, alcohol reduction, and male circumcision for HIV negative men in serodiscordant relationships during clinic visits. Health care providers will also assess and treat sexually transmitted infections (STIs), and provide basic contraceptives and brief safer pregnancy counseling.

In addition, trained lay counselors will deliver HIV prevention interventions in the clinics. Lay counselors will be persons without medical training, many of whom will be people living with HIV, who will be trained to provide counseling and services to HIV-positive patients and their families in HIV care and treatment settings. They will provide HIV prevention counseling, promote HIV testing of partners and children (and provide HIV testing where allowed by national guidelines), and counsel HIV-positive patients on medication adherence and alcohol use.

The prevention interventions will be evaluated as a package in HIV clinics in three sub-Saharan African countries: Kenya, Namibia, and Tanzania. This project will be a longitudinal group-randomized trial and will include 9 intervention clinics (3 per country) and 9 comparison clinics (3 per country). Two hundred patients per clinic (total N = 3600) will be followed for 12 months to evaluate intervention effectiveness. This evaluation will examine the effectiveness of the HIV prevention interventions delivered by health care providers and lay counselors on patient-level outcomes such as risky sexual behavior, disclosure of HIV status, partner HIV testing, alcohol use, HIV antiretroviral (ARV) medication adherence, STIs pregnancies, and contraceptive use.

In addition to the patient outcomes, the acceptability of the interventions and materials, as well as the feasibility of integrating the interventions into HIV care and treatment settings, will be assessed.

Data will be collected via patient interviews, health care provider and lay counselor questionnaires, observations of health care provider and lay counselor patient visits, patient medical chart review, and review of clinic service data. Data collection will occur over a 12 month period.

**Acronyms**

ART Antiretroviral Therapy

ARV Antiretroviral medication

AUDIT Alcohol Use Disorders Identification Test (World Health Organization)

HIV Human Immunodeficiency Virus

AIDS Acquired Immunodeficiency Syndrome

CDC Centers for Disease Control and Prevention (United States)

CD4 CD4 lymphocyte or helper T cell (a type of white blood cell)

DoD Department of Defense (United States)

DHS Demographic and Health Survey

GAP Global AIDS Program (CDC)

GEE Generalized estimating equations

GRT Group-randomized trial

HCP Health care provider

ICAP Columbia University International Center for AIDS Care and Treatment Programs (Kenya, Tanzania)

ICC Intraclass correlation coefficient

IMAI Integrated Management of Adolescent and Adult Illness (World Health Organization)

ITT Intent-to-treat

KEMRI Kenya Medical Research Institute

LC Lay counselor

MOH Ministry of Health (Kenya, Tanzania)

MOHSS Ministry of Health and Social Services (Namibia)

MOS-HIV Medical Outcomes Survey for people living with HIV

NIMR National Institute of Medical Research (Tanzania)

OGAC Office of the Global AIDS Coordinator (United States)

PEPFAR President’s Emergency Plan for AIDS Relief (United States)

PLWHA People Living with HIV/AIDS

PMTCT Preventing Mother-to-Child Transmission of HIV

PwP Prevention with Positives

STI Sexually Transmitted Infection

USAID United States Agency for International Development

USG United States Government

WHO World Health Organization

5As Assess, advise, agree, assist, and arrange (World Health Organization)

**Project Investigators and Roles**

Institutional Review Boards (IRBs)

The project protocol will be reviewed and approved by the IRBs below prior to project implementation.

Atlanta, GA: CDC, FWA00001413

New York, NY: Columbia University, FWA00002636

Kenya: KEMRI (Kenya Medical Research Institute), FWA00002066

Namibia: Ministry of Health and Social Services, FWA00012477

Tanzania: NIMR (National Institute of Medical Research), FWA00002632

**CDC Atlanta**

CDC is the lead agency and is providing funding to countries for conducting this project. Responsibilities include managing the design of the project, instrument development, and evaluation. The CDC research team consists of the following members:

Pamela Bachanas, PhD, CDC Principal Investigator and Project Officer

Janet Moore, PhD

Daniel Kidder, PhD

Steve Flores, PhD

**Columbia University International Center for AIDS Care and Treatment Programs (ICAP) – New York**

ICAP-NY is the organization that provides technical support to ICAP-Kenya and ICAP-Tanzania. Responsibilities include providing technical assistance with project design, instrument development, and implementation.

Andrea Howard, MD

**Kenya Project Team**

CDC - Kenya

Marta Ackers, MD, MPH

Elissa Margolin, MPH

Odylia Muheje, MA

ICAP – Kenya

ICAP-Kenya is the organization that provides support to the Ministry of Health (MOH) HIV care and treatment sites that will participate in this project. Responsibilities include providing assistance with project design and instrument development. ICAP-Kenya staff will be responsible for implementing the project and data collection in the clinics.

Muhsin Sheriff, MD, MPH

Emily Koech, MD, MMeD

**Namibia Project Team**

The Ministry of Health and Social Services (MOHSS) oversees the HIV care and treatment sites that will participate in this project. CDC-Namibia’s responsibilities include providing assistance with project design and instrument development. MOHSS staff will be responsible for implementing the project and data collection in the clinics.

CDC - Namibia

Nick DeLuca, PhD

Jeff Hanson, PhD

**Tanzania Project Team**

CDC – Tanzania

Thomas Finkbeiner, MD, MPH

Irene Benech, MD

ICAP - Tanzania

ICAP-Tanzania is the organization that provides support to the MOH HIV care and treatment sites that will participate in this project. Responsibilities include providing assistance with project design and instrument development. ICAP-Tanzania staff will be responsible for implementing the project and data collection in the clinics.

Harriet Nuwagaba Biribonwoha, MD, PhD

Redempta Mbatia, MD, MSc EPi, DLSHTM

**INTRODUCTION**

**Literature review and background**

Prevention interventions for HIV-positive persons are an essential part of a comprehensive HIV prevention strategy. Although early prevention efforts focused almost exclusively on HIV-negative individuals, more recent efforts have expanded to include prevention with HIV-positive individuals. Changes in the risk behaviors of HIV-positive persons are likely to have larger effects on the spread of HIV than comparable changes in the risk behaviors of HIV-negative persons (King-Spooner, 1999). The goals of prevention interventions with HIV-positive individuals are to reduce the spread of HIV to sex partners and children and to improve the health and quality of life of HIV-positive persons.

An increasing number of HIV-positive persons are being identified through counseling and testing activities and enrolled in HIV care and treatment programs, creating an excellent opportunity for health care providers to reach large numbers of individuals with highly effective prevention interventions (CDC, 2003; Gayle, 2004; Herbst et al., 2005; Schreibman & Friedland, 2003). The World Health Organization (WHO), CDC, and PEPFAR strongly encourage integration of HIV prevention interventions into routine care and treatment for HIV-positive persons (CDC, 2003; PEPFAR, 2007; WHO, 2008). Patients have regular contact with their health care providers in these settings, offering opportunities for ongoing intervention. Additionally, patients typically consider providers to be trusted sources of health information and are accustomed to following providers’ recommendations. Several HIV prevention activities are well-suited for these settings: 1) provider- and counselor-delivered behavioral risk reduction interventions and condom distribution, 2) encouragement of disclosure to partners and partner HIV testing, 3) assessment and treatment of sexually transmitted infections (STIs), and 4) provision of family planning services.

There are several HIV prevention behaviors that are critical for decreasing HIV transmission risk that can be addressed with HIV-positive patients in care and treatment settings. In generalized, high prevalence epidemics, many new infections result from multiple, often concurrent sexual partnerships. Thus, reducing the number of sex partners is an important HIV prevention strategy (Halperin & Epstein, 2004; Morris & Kretzschmar, 1997). In addition, using a condom at each sexual encounter is a highly effective method of lowering HIV transmission risk. However, rates of condom use are very low in sub-Saharan Africa, with estimates ranging from 4% to 28% for the most recent sex act (Cleland & Ali, 2006; Desgrees-du-Lou et al., 2002; Kapiga & Lugalla, 2003). This is particularly important in sub-Saharan Africa, as the rate of serodiscordance among spouses and cohabitating partners is high. For example, in Zambia and South Africa, approximately 20% of couples in the general population were found to be HIV serodiscordant (Lurie et al., 2003; McKenna et al., 1997). In Kenya, approximately 50% of HIV-positive persons have an HIV-negative spouse or cohabitating partner (Central Bureau of Statistics, 2003). This high rate of serostatus discordance suggests that large numbers of married and cohabitating couples in the general population are at high risk for HIV transmission. In addition, rates of disclosure of HIV status are often low, resulting in many individuals who do not know that their spouse or partner is living with HIV. For example, several studies have shown that only one-fourth to one-half of HIV-positive individuals disclose their serostatus to their regular sex partners (Antelman et al., 2001; Medley et al., 2004; WHO, 2003). There are many reasons why people do not disclose their status to partners. For instance, many women, in particular, fear losing their homes or livelihood if they disclose their HIV status to their spouse or partner (WHO, 2003).

These findings document the importance of addressing disclosure, partner testing, condom use and partner reduction in prevention efforts with HIV-positive individuals. However, few, if any, standardized HIV “positive prevention” intervention programs have been implemented and evaluated in health care settings in sub-Saharan Africa. Two brief provider-delivered prevention interventions for HIV-positive persons have been evaluated in the U.S. (Fisher et al., 2006; Richardson et al., 2004), and are well-suited for adaptation to the African HIV clinic setting. Both interventions include brief discussions with patients during routine clinic visits about disclosure to partners and risk reduction strategies including correct and consistent use of condoms. Richardson et al. (2004) reported that their brief provider-delivered discussions with loss-framed messages (emphasis on harmful nature of risk behaviors) resulted in reductions in unprotected sex acts with multiple sex partners in HIV-positive individuals. Similarly, Fisher et al. (2006) found that HIV-positive patients who received a provider-delivered prevention intervention had significantly fewer unprotected sex acts than HIV-positive persons who did not receive the intervention. Together, these studies show that health care providers can deliver brief prevention messages to their patients during routine clinic visits in busy medical clinics that result in reductions in patients’ risky sexual behaviors.

Family planning is also an important area for health care providers and counselors to discuss with their patients. Approximately half of HIV-positive women sampled in a recent Demographic and Health Survey (DHS) in Kenya and Uganda reported that their last pregnancy was unplanned or unwanted (54% and 49%, respectively; Bunnell, 2007). Further, in Kenya, one factor associated with unplanned pregnancy was unmet need for family planning (Magadi, 2003), and among HIV-positive women who did not want more children the majority (64%) reported that their needs for contraception were not being met (Bunnell, 2007). Given that sexual activity tends to increase as health is restored in HIV-positive persons on antiretroviral therapy (ART; Bunnell et al, 2006a & 2006b), and women report low rates of condom use in relationships (Cleland & Ali, 2006), addressing the family planning needs of HIV-positive persons is essential to reduce the number of unwanted and unplanned pregnancies and ultimately to reduce the number of HIV-positive children. However, a significant proportion of HIV-positive persons choose to have children with their partner (Mpangile et al., 2006; Nakayiwa et al., 2006). For example, in Nigeria among discordant couples, one study found that over half who already had children, as well as all of those who did not, desired and planned to have children in the future (Oladapo et al., 2005). Counseling on safer pregnancies and reducing chances for transmission to children for HIV-positive couples who desire children is also critical for prevention efforts. WHO (2008) strongly encourages counseling and services to prevent unintended pregnancy, foster lower risk pregnancy in those who desire children, and prevent maternal-to-child transmission. While family planning services are available to some HIV-positive women through family planning clinics, providers in family planning clinics may not know the serostatus of their patients or may not have the specialized training and knowledge to meet the particular family planning needs of HIV-positive women and couples. Further, given the added time and transportation burden of another clinic visit, many women do not attend these clinics. To date, family planning services for HIV-positive women have rarely been integrated into HIV care and treatment settings, leaving missed opportunities to offer critical prevention counseling and contraceptives to female patients and couples.

STIs are prevalent in populations in sub-Saharan Africa, especially among HIV-positive persons (Buve et al., 2001a & b; Watson-Jones et al., 2000). Two decades of evidence suggested that genital ulcer diseases and other STIs facilitated the sexual transmission of HIV. STIs increase HIV shedding in the genital tract thereby increasing infectiousness (Cohen, 1997; Nagot et al., 2007). STIs also increase susceptibility to HIV by recruiting HIV-susceptible inflammatory cells to the genital tract as well as by disrupting mucosal barriers to infection (WHO/UNAIDS, 2006). However, findings have been mixed on the effect of STI control on prevention of HIV transmission and acquisition. Specifically, a number of randomized control trials have failed to show that treating STIs had a significant impact on HIV incidence (Celum et al., 2009; Kaul, et al., 2004; Wawer et al., 1999; Kamali et al., 2003; Gregson et al., 2007; Celum et al., 2008).

Although the data are unclear on the role of STI treatment on HIV acquisition and transmission, STI management remains important for people living with HIV. Specifically, incident STIs in HIV-positive persons are an indicator of unprotected sex and suggest a need to address sexual risk behavior in order to prevent transmission of HIV to partners. In addition, partner management of STIs provides an opportunity for partner HIV testing, HIV disclosure support, and either discordant couples counseling or HIV care services for concordant positive couples. Lastly, STI management remains a component of quality HIV care services as well as a critical factor for STI control. In general, WHO’s guidelines recommend syndromic management of STIs in resource-limited settings due to lack of equipment and trained staff for diagnostic testing, as well as counseling to reduce ongoing risky behavior (WHO, 2007). However, in sub-Saharan Africa as elsewhere, many HIV clinics do not routinely evaluate or provide STI treatment to their patients unless the patient complains to the clinician. Patients are often referred to an outpatient clinic for STI assessment and treatment but they frequently do not access services. Therefore, assessing and treating patients for STIs as part of routine care for PLHIV is an important recommendation for health care providers (WHO, 2008).

The rate of alcohol use in HIV-positive persons in sub-Saharan Africa is high (Kiragu et al., 2006; WHO, 2006a). Alcohol use is associated with both increased risky sexual behavior (Cook et al., 2006; Fritz et al., 2002) and reduced adherence to ARVs (Braithwaite et al., 2005; Chesney et al. 2000; Kalichman et al., 2007a; Kangudie et al., 2006). A counseling intervention among STI patients in South Africa reduced alcohol use, which in turn was associated with decreased sexual risk behaviors (Kalichman et al., 2007b). However, there are few resources for substance use counseling in most clinical settings in sub-Saharan Africa, and countries vary significantly in the availability of treatment programs for persons with alcohol use disorders. Currently, there is no standard intervention for addressing alcohol misuse among HIV-positive persons.

In summary, research in the U.S. has shown that health care providers can effectively discuss HIV prevention with patients during routine clinic visits (Fisher et al., 2006; Richardson et al., 2004). However, there are significant barriers to this task in sub-Saharan Africa. The inadequate number of health care workers in clinics and large numbers of patients lead to heavy patient loads and severe time constraints for health care providers. Consequently, providers often have little time to discuss HIV prevention issues with their patients. Yet many patients need in-depth discussions on prevention issues, such as overcoming barriers to disclosure, partner testing, and negotiating condom use with partners. In some HIV clinical settings in sub-Saharan Africa, lay counselors have been placed in clinics to provide ARV medication adherence and positive living counseling. Several countries have found that including lay counselors in the clinic to provide these services is a feasible model of task-shifting to reduce health care provider burden. For example, Namibia recently began placing lay counselors in HIV clinics where they conduct HIV testing and counsel patients on adherence and positive living. To date, there are no clinic-based programs for lay individuals focusing on prevention issues for HIV-positive persons. Incorporating lay counselors, many of whom are HIV-positive, into clinic settings offers a cost-effective and supportive model for delivering prevention counseling and partner testing to HIV-positive patients in care and treatment settings. These lay counselors can reinforce prevention messages delivered by health care providers and assist patients in overcoming barriers to engaging in safer behaviors.

**Study Justification**

It is important to provide HIV prevention messages and services to HIV-positive individuals, yet few clinics in sub-Saharan Africa have integrated HIV prevention into the care and treatment of HIV-positive persons. To address the prevention needs of those who are HIV-positive, care and treatment programs should be multifaceted and comprehensive, incorporating key behavioral prevention messages, STI management, family planning counseling and services, alcohol assessment and intervention, and medication adherence counseling. CDC’s Prevention Branch of GAP, in collaboration with the US Agency for International Development (USAID), and the US Department of Defense (DoD), has developed a package of clinic-based HIV prevention interventions for HIV-positive individuals that includes behavioral and biomedical interventions. The interventions will be delivered in HIV care and treatment clinics by trained health care providers and lay counselors.

The components of the HIV prevention package include brief health care provider-delivered prevention messages (i.e., disclose to partner, get partner tested, use condoms during every sexual encounter, adhere to ARVs, reduce alcohol use), STI assessment and treatment, and brief family planning counseling and services. Lay counselors will be integrated into the HIV care and treatment clinic and will provide more in-depth HIV prevention counseling, discuss barriers to prevention, deliver a brief alcohol intervention (if indicated), offer medication adherence counseling, and provide HIV testing (if allowed).

This prevention intervention package will be evaluated to assess the impact of the package (not individual components) on HIV-positive patients’ sexual risk behaviors, alcohol use, unintended pregnancy outcomes, and STIs, as well as its impact on delivery of services in the clinics, including the number of contraceptives dispensed, and number of counseling sessions provided. The acceptability of the intervention and the feasibility of integrating the prevention intervention package into HIV care and treatment clinics will also be assessed.

**Intended use of study findings**

This project will evaluate the impact of a clinic-based HIV prevention intervention adapted for, implemented, and evaluated in sub-Saharan Africa. Effects of the intervention on HIV transmission risk behaviors will be evaluated. Also examined will be the feasibility of integrating this comprehensive prevention intervention package into HIV care and treatment clinics in resource-limited settings. Findings will be used to inform plans for scaling-up prevention interventions in other sub-Saharan African HIV clinics.

**Study design and locations**

Study design

This project will be a longitudinal group-randomized trial, randomized at the clinic level. Six HIV care and treatment clinics in each of three countries (Kenya, Namibia, and Tanzania) will participate (18 clinics total). The six clinics in each country are matched based on size, health care provider/patient ratio, and services provided (e.g., number of health care providers, size of clinic and number of HIV-positive patients seen, level of care provided). Intervention status was randomly assigned to one clinic in each matched pair.

Health care providers and staff will be trained to deliver the HIV prevention interventions to all patients in clinics randomly assigned as intervention sites. In the comparison site clinics, providers and staff will deliver their usual standard of care services. Following completion of the project, health care providers in the comparison clinics will be trained to deliver the HIV prevention interventions.

A measurement cohort of 3600 HIV-positive patients will be randomly recruited to participate from each of the 18 clinics in the 3 participating countries (n=200 per clinic). Participants in the intervention and comparison groups will be assessed at 3 time points: Baseline (enrollment), 6-, and 12-months. After participants have completed the baseline assessments, the prevention interventions will be implemented by health care providers and lay counselors in the intervention clinics.

Assessment of patients will include completion of an interviewer-administered questionnaire. At each assessment, trained interviewers will ask participants about their sexual behavior, STI symptoms, pregnancy status and intentions, contraceptive use, alcohol use, HIV ARV medication adherence and adherence to co-trimoxazole prophylaxis, partner HIV testing history and HIV status, mood and perceived social support (see Appendix H). To the extent possible, participants will be interviewed following a routine HIV clinic visit in order to reduce burden on patients.

In addition to information collected from patient interviews, data will be collected from patient chart review (Appendix P), and questionnaires administered to health care providers (Appendix I, J) and lay counselors (Appendix K). Fidelity to the intervention protocol will be assessed by observations of patient visits with health care providers (Appendix M) and lay counselors (Appendix N), as well as services delivered in the clinics (i.e., STI treatment, family planning services; Appendix O).

Study locations

Three countries in sub-Saharan Africa will participate in this project: Kenya, Tanzania, and Namibia. In each country 6 HIV care and treatment clinics have been selected for project participation. The sites chosen are typical District-level hospitals with HIV outpatient facilities and with patient populations representative of the national HIV epidemics. The participating intervention and comparison HIV clinics will be similar in their level of HIV service provision at baseline. The participating clinics are as follows:

|  | **Intervention clinics** | **Comparison clinics** |
| --- | --- | --- |
| Kenya:  Working with Columbia-ICAP in Ministry of Health facilities. | | |
|  | Thika District Hospital | Nyeri Provincial Hospital |
|  | Ruiru Health Centre | Kangema Health Centre |
|  | Kiambu District Hospital | Muranga District Hospital |
| Namibia:  Ministry of Health and Social Services facilities. | | |
|  | Swakopmund Hospital | Walvis Bay Hospital |
|  | Oshakati State Hospital | Onaandjokwe Hospital |
|  | Outapi State Hospital | Engela Hospital |
| Tanzania:  Working with Columbia-ICAP in Ministry of Health facilities. | | |
|  | Tumbi Regional Hospital, Kibaha, Pwani region | Mnazi Moja Regional Hospital, Unguja, Zanzibar |
|  | Kisarawe District Hospital, Kisarawe, Pwani region | Bagamoyo District Hospital, Bagamoyo, Pwani region |
|  | Mchukwi Mission Hospital, Rufiji, Pwani region | Mkuranga District Hospital, Mkuranga, Pwani region |

**Objectives**

1. To evaluate the effectiveness of integrating an HIV prevention intervention package into routine care and treatment of HIV positive patients in HIV clinics in resource-limited settings. The impact of the intervention package on the following outcomes will be assessed:

Behavioral

- Risky sexual behaviors (i.e., unprotected sex, number of sex partners)
- HIV testing of partners
- Disclosure of HIV status to sex partners
- Alcohol use
- HIV ARV medication adherence

Biologic

- Unintended pregnancies
- STIs (i.e., urethral discharge. Vaginal discharge will not be examined as an outcome due to the increased likelihood of overestimating STIs in HIV-positive women with candidiasis or bacterial vaginosis.)

Service Delivery

- Family planning counseling (e.g., safer pregnancy) and services (e.g., contraceptive pills and injections dispensed, safer pregnancy discussions, PMTCT referrals)

2. To assess the feasibility and acceptability of integrating a prevention intervention package into the routine care and treatment of HIV-positive patients in resource-limited HIV clinics in three sub-Saharan African countries.

**Hypotheses**

Project hypotheses focus on the effectiveness of the prevention intervention package on behavioral outcomes, biologic outcomes, and delivery of services in the HIV care and treatment settings. Intervention clinics will integrate the HIV prevention intervention package into routine care of HIV-positive patients; comparison clinics will provide standard of care to HIV-positive patients.

Compared to participants attending comparison HIV clinics, participants at intervention clinics will be more likely over a 12-month period to report or show a(n):

Behavioral

- Decrease in unprotected vaginal and anal sex
- Decrease in number of sex partners
- Increase in number of sex partner(s) getting an HIV test
- Increase in disclosure of HIV status to sex partner(s)
- Decrease in alcohol use
- Increase in adherence to HIV antiretroviral medications

Biologic

- Decrease in unintended pregnancy or partner pregnancy
- Decrease in STIs (i.e., urethral discharge)

Service Delivery

Compared to the comparison clinics, the intervention clinics will:

- Provide more family planning counseling (e.g., safer pregnancy) and services (e.g., contraception) to HIV-positive women and their partners

**General Approach**

The general approach will be confirmatory (hypothesis testing). The project will use data collected from the baseline and follow-up measures to compare changes over time between persons at the clinics randomly assigned to receive the HIV prevention intervention package and persons who are at the comparison clinics receiving the standard of care for that country. Quantitative analyses will be conducted to examine the research questions outlined above and to test associated hypotheses.

**PROCEDURES/METHODS: DESIGN**

**How Study Design Addresses Research Questions and Meets Objectives**

The project design is a longitudinal group randomized trial that will be conducted with 18 project sites in 3 sub-Saharan African countries. Each country will have 6 project sites that are paired and randomized to either the intervention or comparison arm. The intervention clinics will implement the HIV prevention intervention package, and health care providers and lay counselors will be trained on implementation. The comparison clinics will continue to provide the customary care services being provided.

Questionnaires for clinic patients will assess HIV risk behaviors, treatment adherence, disclosure of HIV status to sex partners, partner testing history, alcohol use, STI symptoms and treatment, and family planning services. These data will allow us to test the hypotheses that the HIV prevention intervention decreases HIV risk behaviors and improves patient health outcomes.

Patient chart review will provide information about patient health status, frequency of getting HIV/ARV medications, STI syndromes identified and treated, contraceptives, pregnancy, and scheduled appointments.

Process assessments and observations of health care provider and lay counselor patient visits will provide information about the types of HIV prevention information being provided to patients in order to assess intervention fidelity. Health care providers and lay counselors will also complete questionnaires to assess the acceptability and feasibility of the intervention.

**Audience/stakeholder participation**

The primary audience for this project will be health care providers caring for HIV-positive individuals in resource-limited clinics in sub-Saharan Africa and people living with HIV/AIDS. We have worked collaboratively with health care providers from HIV care and treatment sites from all three participating countries in the development of this protocol, the intervention materials, and the evaluation plan. Input on the adaptation and development of the materials and interventions was obtained from local health care providers and staff in participating and additional sub-Saharan African countries. In addition, the materials have been field-tested and revised based on feedback received from local health care providers and other health officials. We also have obtained input from HIV-positive patients attending care and treatment clinics in these countries on some of the prevention materials and messages, as well as on the proposed protocol for integration of prevention services into HIV clinics. Key stakeholders for this project have also been involved in the development of all aspects of the intervention and protocol, including representatives from multiple US Government (USG) agencies (CDC-Kenya, CDC-Namibia, CDC-Tanzania, USAID-Washington, USAID-Namibia, DoD, the Office of the Global AIDS Coordinator (OGAC), Namibia Ministry of Health and Social Services (MOHSS), and Columbia-ICAP (New York, Kenya, and Tanzania). The Ministries of Health in Kenya and Tanzania are stakeholders and partners with the USG in-country teams. They are aware of these efforts and are supportive of the activities being conducted in this project.

We have a project steering committee with in-country representatives and experienced providers who have provided guidance and input into this project. This committee consists of representatives from CDC Headquarters (Atlanta); the OGAC; members of the OGAC Task Force on Prevention with Positives, which includes USG agencies participating in PEPFAR (i.e., CDC, USAID, and DoD); and representatives from the participating country project teams. All decisions related to the design and implementation of the project will be made by majority agreement of Steering Committee members.

**Cost benefit/prevention effectiveness**

The cost effectiveness of the prevention intervention compared to standard of care also will be evaluated. Specifically, this aspect of the evaluation will involve estimating incremental program costs at the facility level in the clinics implementing the intervention package compared to standard of care clinics. Costs associated with training health care providers, commodities required for the intervention, and new staff needed to implement the intervention (lay counselors) will be assessed. Also, an aggregate outcome measure of effectiveness to link with the costing data will be developed. This will be an aggregate of other outcome measures already being collected as part of this project; therefore, no additional human subjects or consent issues are applicable. The CDC-Atlanta team will obtain additional consultation from the costing team at CDC to refine the assessments and plan for collection of costing data as the project evolves.

**Study timeline**

The following describes the project timeline after IRB approvals are obtained.

- October 2008: Pilot and revise questionnaires
- January – February 2009: Project enrollment and baseline
- February – March 2009: Intervention training for health care providers and lay counselors
- September – October 2009: 6-month Follow-up
- April – May 2010: 12-month Follow-up
- May – July 2010: Data cleaning and data analysis
- July 2010: Project results available

**PROCEDURES/METHODS: STUDY POPULATION**

**Description and source of study population and catchment area**

The primary project participants will be HIV-positive persons who are receiving HIV care and/or treatment in the project clinics. The catchment area will be the service area of the project clinics, which vary in size. Data will be also collected from health care providers and lay counselors in the project clinics in order to determine what HIV prevention messages are being provided to patients and, in the intervention clinics, to determine intervention fidelity.

**Case definitions**

Each participant will have taken an HIV test, been diagnosed with HIV infection, and begun attending the HIV clinic prior to enrolling in the project. Each participating clinic (intervention and comparison) will enroll 200 participants. Of those patients, approximately half will be receiving HIV ARV medications and half will only be receiving care for their HIV disease and will not yet be eligible for or prescribed ARVs. Patients are eligible to participate regardless of the amount of time since diagnosis with the infection.

Health care providers (physicians, clinical officers, and nurses) and lay counselors will also be participants in this project. They will be observed and interviewed to determine the types of HIV prevention messages being provided to clinic patients. They will be asked about acceptability and feasibility of implementing the intervention package.

**Participant inclusion criteria**

Clinic patients

- At least 18 years old
- HIV-positive patients receiving care at a project clinic and seen at the clinic at least twice prior to enrollment (half of patients will be taking ARVs)
- Sexually active within the past three months
- Planning to attend the clinic for at least 1 year
- Able to provide informed consent to participate in the project
- Able to conduct interview in one of the following languages:

Kenya: English, Kiswahili and Kikuyu

Namibia: English, Oshiwambo, Damara-nama, Otjihero, Afrikaans

Tanzania: English, Kiswahili

Health care providers and lay counselors

- At least 18 years old
- Fluent/literate in the language selected for the project training
- Planning to remain at the clinic for one year
- Willing to complete project questions and agree to be observed during patient visits
- Physicians, clinical officers, and nurses who are employees of the project clinic and providing care and treatment to patients in the HIV project clinics
- Lay counselors who are providing HIV prevention services in the project clinics

**Participant exclusion criteria**

Clinic patients

- Younger than 18 years of age
- Not sexually active within the last three months
- Planning to move from the vicinity of the clinic within one year
- Not enrolled in the HIV clinic and/or have not been seen for at least two clinic visits
- Cannot provide informed consent
- Patients who are acutely ill or are determined by clinical staff to be too ill to participate
- Spouses or identified partners of participating patients
- Pregnant women and male partners of pregnant women, as family planning counseling and unintended pregnancy are some of the primary study outcomes
- Not able to complete interview in one of the languages in the inclusion criteria
- Participated in pilot study

Health care providers and lay counselors

- Younger than 18 years of age
- Not fluent/literate in the language selected for the project training
- Planning to leave the clinic within one year
- Not willing to complete project questions
- Do not agree to be observed during patient visits

**Justification of exclusion of any sub-segment of the population**

Except for the exclusion criteria described above, sub-segments of the population will not be excluded from participation. Women who know they are pregnant at the time of enrollment and male partners of women pregnant at time of enrollment will be excluded from the study as family planning counseling and unintended pregnancy are some of the primary study outcomes. If participants (or participants’ partners) are already pregnant when they enroll in the study it would not be possible to examine these outcomes.

**Estimated number of participants**

Two hundred patients per clinic (total N=3600 participants) will be recruited and enrolled in this project. It is important to note that the health care providers and lay counselors will be providing the intervention components to all clinic patients, and those participating in the study will only be a sample of the clinic patients receiving the intervention. Each clinic will enroll approximately 100 participants who are receiving ARV medications for their HIV, and approximately 100 participants who are receiving care for their HIV disease but who are not taking ARVs. The rationale for this is to have a sample with the broadest representation of the HIV-positive population, including those healthier and more likely to be sexually active, as well as those on ARVs who come to clinic regularly but who may be sicker. Individual country requirements for ART eligibility and availability of ARVs will be the determining factors for health care providers deciding when patients receive ART. Treatment and medication status will be assessed on the screening form in order to track the number of participants enrolled in each group, and enrollment of each group will stop at each site after the required numbers of participants in care and on treatment have been met. In addition, each site will attempt to enroll an equal number of male and female participants.

There will be approximately 10 health care providers at each clinic who will be eligible to participate for a total of about 180 health care providers. There will also be about 4 lay counselors in each of the 9 intervention clinics for a total of about 36 lay counselors who will be eligible for project participation. In addition, counselors in comparison clinics who are providing services to HIV-positive patients (e.g., adherence counseling) will be eligible to participate in the evaluation to provide information about services provided to patients.

**Sampling, including sample size and statistical power**

Participants will be sampled from the database of patients already receiving care at the project clinics. Information about gender, whether on ART, and last clinic visit will be used to create a list of potential participants. From that list, patients will be randomly selected to create a list of patients to approach about participating in the project. When patients arrive at the clinic the list will be checked and if their name is on it they will be asked if they are interested in participating in the project (see enrollment section below for further description). If they are not, their name will be removed from the list. Each clinic will enroll approximately half of each gender and half who are on ART. A tally of persons in each of the gender by ART cells will be kept updated daily. As each of the cells near the enrollment targets the study coordinator will monitor the potential participant list to ensure that appropriate numbers of participants are in each group.

The primary outcome variable for the project will be participant self-report of any unprotected sex in the past 3 months. Prior studies have found rates of condom use to be very low in the areas in which this project will be conducted (Arthur et al., 2007; Feldblum et al., 2000; Kapiga et al., 2006; Mwaluko et al., 2003; Norman, 2003; Stanton et al., 1999; Ross et al., 2007; Watson-Jones et al., 2007). We will assume a rate of at least 50% for any unprotected sex in the past 3 months in the comparison condition for sample size estimation.

Although the proposed project includes measurements at three time points (baseline, 6 months and 12 months), sample size estimates are presented for the simple difference between proportions in the two project conditions at the 12-month follow-up only. This approach is conservative because analytic approaches taking into account the multiple observations on each participant will be more powerful. We use the cross-sectional approach for sample size estimation because no prior data are available on which to base prediction of the pattern of intervention effects over time and the magnitude of the over-time correlation of groups and group members that we can expect in this project. Our sample size estimates do take into account expectations of attrition in the project cohort, which we expect to be moderate (at most 30% at the 12-month follow-up).

In the following sample size table, we present calculations for the number of clinics needed for at least 80% power to detect differences of varying size between the intervention and comparison conditions with a two-sided test and α=0.05. Sample size estimates are based on a formula in Murray (1998) for group-randomized trials:

Where g is the number of groups per condition, and are the event rates in the two project conditions, *m* is the number of members per group (clinic), is the intraclass correlation coefficient measuring the correlation of members nested within groups, nested within project conditions, and and are critical values from the t-distribution.

Table 1 presents the sample sizes necessary for at least 80% power to detect a 20-30% reduction in the rate of unprotected sex in the past 3 months in the treatment condition compared to the comparison condition at the 12-month follow-up. We use 50% as the rate of unprotected sex in the comparison condition for sample size estimation; if the actual rate is higher or lower with the same reduction in the treatment condition, power will be greater, as variance in the binomial distribution is largest at 50% and decreases as the event rate moves away from 50% in either direction. Power calculations for group-randomized trials also need to take the expected intraclass correlation coefficient (ICC) into account to have adequate power. However, there are no prior published reports of ICCs for sexual risk behaviors among clinic attendees in sub-Saharan Africa on which to base our power calculations. A prior study did report an ICC of .0011 for STI prevalence at the community level in Kenya (Feldblum et al., 2000), and another study presented an ICC of about .01 for any unprotected sex in the past 90 days in a neighborhood-randomized trial in the U.S. (Pals et al., 2008). ICCs are generally larger for behavioral variables than for biologic variables and also generally increase as the group size decreases (e.g. larger for clinics than for communities). Murray and Blitstein (2003) showed that including multiple timepoints in analytic models can substantially reduce the ICC. Based on these prior results, we use ICCs of .01 and .02 for sample size estimation.

**Table 1. Number of groups and members per condition needed for at least 80% power to detect a 20-30% difference between intervention and comparison conditions in report of any unprotected sex in the past 3 months (assuming intraclass correlations of 0.01 or 0.02).**

| **P1** | **P2** | | **ICC** | **Absolute Difference** | **% Difference** | **Members per group enrolled** | **Members per group retained** | **Number of groups needed per condition** |
| --- | --- | --- | --- | --- | --- | --- | --- | --- |
| .50 | .3875 | | .01 | .1125 | 22.5 | 200 | 140 | 7 |
| .50 | .3750 | | .01 | .1250 | 25 | 200 | 140 | 6 |
| .50 | .3625 | | .01 | .1375 | 27.5 | 200 | 140 | 5 |
| .50 | .3500 | | .01 | .1500 | 30 | 200 | 140 | 4 |
| .50 | | .3750 | .02 | .1250 | 25 | 200 | 140 | 9 |
| .50 | | .3625 | .02 | .1375 | 27.5 | 200 | 140 | 7 |
| .50 | | .3500 | .02 | .1500 | 30 | 200 | 140 | 6 |

P1=proportion reporting any unprotected sex in the comparison group; P2=proportion reporting any unprotected sex in the intervention group

Table 1 shows that with 9 clinics per condition, each with 200 patients enrolled, the project would have at least 80% power to detect a 25% difference between intervention and comparison (absolute difference of 12.5%) with an ICC of 0.02. If the ICC is smaller or the intervention effect larger, power will be greater then 80%.

Summary

We anticipate enrolling 200 patients per clinic with 9 clinics per condition (3 per condition per country), for a total of 18 clinics and approximately 3600 patients (at least 2520 retained at 12 months). Power calculations demonstrate that this number of participants will allow us at least 80% power to detect a 25% relative difference between conditions for the primary outcome variable of any unprotected sex during the past 3 months, allowing for up to 30% loss to follow-up.

**Enrollment**

Patients arriving at the clinic will provide their health card or name to a clinic staff member, who will check the list of possible study participants for his/her name. If the patient’s name is on the list, the clinic staff member will ask the patient if he/she is interested in hearing about the project. If the patient is interested in hearing about the project, the clinic staff member will introduce the patient to a research study project interviewer or project coordinator. Each interested patient will be taken to a quiet place in the waiting area outside of hearing range of other patients and staff or, if possible, to a private room. They will be told about the project and asked screening questions (Appendix C) to assess project eligibility. Patients who are eligible will be asked to participate in the project but will be assured that their decision about participation will not affect the care they receive in the clinic. Those who decline will be thanked for their time. Those who agree to participate in the project will complete the project consent process (described in the next section) and complete the baseline interview. If the participant is not available at the time of enrollment to complete the interview, he/she will be scheduled to return to the HIV clinic within 10 days to meet with a project interviewer.

**Consent process**

Eligible persons choosing to participate will be shown the consent form (Appendix D). Printed consent forms will be available in the participant’s chosen language and will be read to the participant by the project interviewer in that language. Potential participants will be informed that their participation is voluntary, that they do not have to answer questions that make them uncomfortable, and that all information will be kept private (i.e., no personal identifiers will be used and only summary information across all participants will be reported). Although there are no anticipated risks for participating in the interviews, some individuals may experience emotional discomfort with the questions. If this should occur, participants will be reminded that they are free to withdraw their participation at any time or may refrain from answering any questions that make them uncomfortable. Patients will be informed that their participation or refusal to participate will not in any way affect their care or treatment. They will be invited to ask questions about any aspect of the project and their participation, and the interviewer will answer them. Participants will be informed that all information collected for this project is confidential and will not be shared with any hospital staff (aside from the project coordinator and interviewers) or their family, friends, or employers. If they agree to participate in the project, participants will sign their name on the consent form to indicate they have obtained the information and voluntarily agree to participate. Those who are not able to sign due to literacy issues will provide a thumbprint, as is customary in the participating countries.

Health care providers and lay counselors will have the option of participating in the evaluation of the project. That is, they will be able to choose whether to complete the questionnaires and allow observation of their interactions with patients/participants. The purpose and requirements of participating in the intervention evaluation will be explained and all questions will be answered. If interested in participating in the project, health care providers and lay counselors will read and sign the consent form. They will be informed that their participation is voluntary and that their jobs will not be affected by their participation in this project. They are free to withdraw at any time or refuse to answer any questions that make them uncomfortable. No information gathered from this evaluation will be shared with participants’ co-workers or supervisors.

There will be a master list with each participant’s name and an assigned identification number. That identification number will be written on all data collected; participant names will not appear on any data. Patients, health care providers, and lay counselors who enroll in this project will each be assigned an identification number so that their name is not linked to their questionnaires or data, in order to protect their confidentiality. Only the site project coordinator will have access to the master list so they can ensure that data across project visits can be linked correctly to each participant. The master list will be locked in a filing cabinet in a secure room in the clinic. At follow-up project visits, participants will tell the project coordinator their name, the coordinator will consult the master list for the identification number, and that number will be entered on all forms and in the database. All information collected (medical chart information and interviews) will be stored securely in the researchers’ offices or on computers with secure access. The CDC-Atlanta, ICAP-New York, and in-country research teams will only have access to the data without identifiers that can link to individual patients.

**PROCEDURES/METHODS: VARIABLES/INTERVENTIONS**

**Variables**

Variables will be collected from patients and from clinic charts. The variables will include both behavioral and biologic dependent variables. In addition, we will examine several potential variables that may mediate the hypothesized changes in outcome variables. The following paragraphs describe our primary outcome variables, our primary independent variables, and potential mediating variables.

Primary Outcome Variables

The primary outcome variables will be compared for those in the intervention and those in the comparison arms. These variables have been discussed previously and include self-reported sexual risk behaviors (e.g., any unprotected sex in the past 3 months, number of sex partners, proportion of sex acts that are unprotected with HIV-negative or unknown status partners), disclosure of HIV status to sex partners, partner testing for HIV, alcohol use, HIV medication adherence, STIs (i.e., urethral discharge) unintended pregnancies, family planning and safer pregnancy counseling, and family planning services (e.g., contraception).

Independent Variables

As discussed, we propose conducting a longitudinal cohort project with a two arm design. The intervention group will consist of participants from clinics in which the staff have been trained to provide the HIV prevention intervention package and the comparison group will be participants from clinics providing the current standard of care. For our primary outcomes, we will use an intent-to-treat (ITT) analysis approach. That is, participant data will be analyzed based on the project arm to which they were assigned. Thus, intervention or comparison arm will serve as our primary independent variable in these analyses.

The ITT approach preserves the unbiased comparability between the intervention arm and the comparison arm that results from random assignment, thereby strengthening our project’s internal validity. Furthermore, our primary research questions focus on hypothesized outcomes associated with providing a package of HIV prevention interventions for HIV-positive persons, and analyzing the data based on the project condition to which participants were randomized will allow us to examine the impact of this intervention.

In addition to our primary ITT analysis, we plan to examine other independent variables and their relationship to our outcome variables described above. These variables include number of clinic visits, amount of time spent with health care providers and lay counselors during clinic visits, and prevention activities discussed.

Potential Mediating Variables

Our potential mediating variables include domains such as physical and mental functioning, HIV transmission risk knowledge and perception of HIV transmission risk, social support, sexual self-efficacy, communication with partner, STI symptoms, CD4, and physical and sexual violence. Within each of these domains, our questionnaire will use sets of items to measure various subcategories of these domains. Scores on these items will be used as mediating variables to examine whether they impact our proposed primary outcome variables.

**Study instruments**

Overview

HIV-positive patients will complete a questionnaire administered by a trained interviewer. The questionnaire will assess domains such as sexual behavior, STI symptoms, pregnancy status and intentions, contraceptive use, alcohol use, medication adherence, partner HIV testing history and HIV status, disclosure to partners, mental health, physical health, and perceived social support.

Health care providers and lay counselors will also be administered questionnaires by the interviewers that will assess HIV prevention and clinical services provided to patients, as well as the acceptability and feasibility of implementing prevention interventions into care and treatment settings. The health care providers and lay counselors will also be observed by study staff during a small portion of the clinic and counseling sessions to assess fidelity to the intervention protocol.

The project staff will conduct medical chart and pharmacy record reviews of enrolled patients and will record clinical status and disease progression indicators, pregnancy and STI information, and ARVs dispensed. Clinics will record information on number of contraceptives delivered to HIV-positive women and number of STI syndromes treated in HIV positive patients during the course of the study. Finally, basic statistics on the clinic (e.g., number of patients seen, services provided) will be recorded over the course of the project by the site project coordinator to monitor the impact of the intervention on the clinic service delivery. See Table 2 below for information on which measures will be administered during which time periods.

**Table 2. Data collection time periods for project measures.**

|  | **Baseline** | **6-Month**  **Follow-up** | **12-Month Follow-up** |
| --- | --- | --- | --- |
| **Patient Questionnaire** | X | X | X |
| **Provider Questionnaires**   - Clinic Services - Integration of HIV prevention into care and treatment | X  X | X  X | X  X |
| **Lay Counselor Questionnaire**   - Integration of HIV prevention into care and treatment | X* | X* | X* |
| **Provider and Counselor Observation Checklist** | X | X | X |
| **Medical Chart Review and Clinical Records Review** |  |  |  |
| Clinical Services Form | X | X | X |
| Patient Medical Chart Review Form | X | X | X |

* Only at intervention clinics

***Patient Questionnaire***

The patient questionnaire (Appendix H) will be administered to participants by a trained interviewer at baseline, 6 months, and 12 months. This questionnaire includes questions from several different domains described below.

The patient questionnaire and consent forms will be translated into the eligible languages for each country (Kenya: Kiswahili and Kikuyu; Namibia: Oshiwambo, Damara-nama, Otjihero, Afrikaans; Tanzania: Kiswahili). Translation will be done by organizations contracted by CDC (Atlanta or in-country) who have prior experience with translation of health and technical information. Part of the translation process is to conduct quality control reviews of the translated documents to verify the accuracy of the translation and to ensure that the original intent of the questions has not been changed. The translation process will involve translating the text from English to the appropriate language, back translating into English and checking for errors, and correcting errors. The questionnaire will be administered to the participant in the language they select by interviewers fluent in that language. All interviewers will also be fluent in English.

Sociodemographics: This section assesses the basic demographic characteristics of participants including age, gender, education level, ethnicity, religion, relationship status, number of children, employment status and reasons for not working, income and income source, living situation, and travel to clinic. Where possible, questions from the Demographic and Health Survey (DHS) were selected for this measure. Participants will also be asked the date they were diagnosed with HIV, and the date of their most recent HIV test.

HIV testing and care: Information assesses when the participant was first diagnosed with HIV, where tested and why, last HIV and CD4 test, most recent CD4 count, length of time and locations where receiving HIV care, and number of visits to clinic in the past year.

Mental and Physical Health: The Medical Outcomes Survey for people living with HIV (MOS-HIV; Mast, 2004) will assess mental and physical functioning primarily over the past 30 days. Participants will also complete the Center for Epidemiologic Studies Depression Scale (Radloff, 1977), which is an assessment of depressive symptoms. This questionnaire asks participants about depressive feelings and behaviors over the past week.

Social Support: The Perceived Availability of Support questionnaire asks about participant’s level of social support. Participants report whether they have access to emotional, physical, and financial support needed to deal with challenging situations.

Alcohol use: The World Health Organization’s Alcohol Use Disorders Identification Test (AUDIT) is a 10-item scale assessing problematic drinking (WHO, 2001). Also included are items from the National Institute on Alcohol Abuse and Alcoholism (NIAAA) Task Force on Recommended Alcohol Questions which assesses frequency and amount of alcohol consumed in the past 6 months (NIAAA, 2003).

HIV antiretroviral medications and adherence: Participants will be asked whether they are currently taking HIV medications (including co-trimoxazole prophylaxis), total time taking HIV medications, current medication regimen and adherence to regimen, reasons for missing doses, and reasons for not taking HIV medications (if not taking).

Disclosure: Participants will be asked about disclosure of serostatus to sex partners, family members, relatives, and friends. They will also be asked whether they are worried about people finding out about their HIV status and what might happen if people found out.

Knowledge of HIV/AIDS transmission: Assesses knowledge of methods for sexually transmitting HIV and sources for HIV/AIDS information.

Sexual Risk Behaviors: Assesses behaviors related to possible sexual transmission of HIV. These include relationship status and sexual behaviors with up to 5 partners, concurrent partnerships, condom use, disclosure of serostatus to sexual partner(s), partner(s) HIV testing and serostatus, sex exchange, and physical and sexual violence experiences.

Sexual Self Efficacy: This will include questions about self-efficacy regarding condom use, discussions with partner about topics such as STDs and condom use.

STIs: Participants will be asked whether they have current symptoms of STIs (discharge, genital sores, pain during urination) and have been treated for STIs during the last 6 months.

Family Planning: Female participants will be asked about pregnancy status, desire to have children in the next year, current contraception use and source, length of time taking contraceptives, reasons for not using contraceptives, and who makes decisions regarding contraceptive use and family planning. Male participants will be asked modified questions about their partner/spouse’s status and intentions/family planning needs.

Prevention Services Received: This assesses participants’ exposure to the HIV prevention interventions from the health care providers and lay counselors in the clinic. Items assess the number of visits to providers and counselors, which HIV prevention topics were discussed, reasons why they did not meet with counselors (if applicable), comfort discussing sensitive topics, and the perceived responsibility of clinic staff to provide HIV prevention messages.

***Health care provider questionnaires***

The health care provider questionnaires will be administered at baseline, 6-months, and 12-months (see Table 2).

Clinical Care Survey: Health care providers in all clinics will be asked to document the types of care they provide to patients (Appendix I). This includes the number of patients they see per day, the amount of time they spend with them, frequency of discussing a variety of topics with patients during routine visits, and whether they provide various services or refer to other clinics for those services.

Integration of HIV Prevention into Care and Treatment: Health care providers at all clinics will provide information to assess whether and how often they addressed the topics in the intervention protocol (Appendix J). This includes use of the job tools/aids, whether they thought it was feasible and important to deliver the messages, and whether patients were comfortable receiving these messages from the health care providers. Additionally, the health care providers will answer questions about the impact of providing the intervention on the patients and the health care providers, as well as perceptions of clinic staff responsibility to provide HIV prevention interventions to patients.

***Lay Counselor Questionnaires***

Integration of HIV Prevention into Care and Treatment: Lay counselors in intervention clinics will provide information to assess whether and how often they addressed the topics in the intervention protocol (Appendix K). This includes the frequency of patient visits, amount of time they spent with patients, frequency of discussing various prevention topics, and use of the job tools/aids. Lay counselors will also answer questions about whether they thought it was feasible and important to deliver the messages, and whether patients were comfortable receiving these messages from the health care providers. Additionally, the lay counselors will answer questions about the impact of providing the intervention on the patients and the counselors, as well as perceptions of clinic staff responsibility to provide HIV prevention interventions to patients.

Lay Counselor Record: Lay counselors will record each patient visit on a form which will include the date of the visit, the length of the session, topics addressed during the discussion (e.g., disclosure, partner testing, adherence, alcohol reduction), next steps or a patient goal that was discussed, and the date of the next appointment with the counselor (Appendix L). This form will be completed for all patients who meet with a lay counselor in the intervention clinics and will be used as a form for counselors to document their interactions with patients. It will be kept in the patient’s chart so that it can be easily accessible to the health care provider as well as the lay counselor. Information from this record will be extracted as part of the medical chart review.

***Provider and Counselor Observation Checklists***

Assessing the effectiveness of the intervention package on behavioral, biologic, and service delivery outcomes depends on whether, and how accurately, health care providers and lay counselors deliver the intervention. In order to determine how the intervention changes service delivery, project staff will observe providers and lay counselors in the intervention clinics during approximately 3% of routine patient visits over the one year project period. Health care providers and counselors (where available) in comparison clinics will also be observed to have a complete assessment of standard of care visits and services provided in these the comparison clinics. At the beginning of each month, days will be randomly selected for observing health care providers and lay counselors. The number of observations per week will depend on the number of patients being seen at each clinic per week. For each observation, the observers will sit in as unobtrusive a place as possible in the room with the health care provider or lay counselor and will note which intervention activities are discussed with participants by health care providers (Appendix M) and lay counselors (Appendix N). Comparisons will then be made between the care provided at intervention clinics and the standard of care being delivered in comparison clinics.

***Medical Chart Review and Clinic Records Review***

Clinic Services: Integrating HIV prevention services into the HIV care and treatment clinic may impact overall clinic services and numbers of patients seen. The Clinic Services Form (Appendix O) will capture HIV services at the clinic level over time. Specifically, the number of patients enrolled at the clinic in care and on treatment, number of patients seen daily and weekly, number of patients who defaulted or were lost to follow-up, number of contraceptives provided to HIV-positive women prescribed through the HIV care and treatment setting, the number of treatment packages for STI syndromes dispensed to HIV-positive patients prescribed through the HIV care and treatment clinics, and number of HIV tests conducted will be recorded over the project period. The project staff will complete this form for intervention and comparison clinics by checking clinic records and log books.

Medical Chart Review: The Patient Medical Chart Review Form will record information from the medical charts of enrolled participants (Appendix P). Information recorded will include dates of HIV clinic visits for the year prior to enrollment, clinical indicators of HIV (e.g., CD4 count, WHO classification), medications, partner’s HIV status, reproductive health history, STI symptoms syndromically identified and STI treatment provided. Other information available in the medical chart will be recorded such as provider-delivered prevention messages, family planning, and STI management. These data will be collected for the year prior and during the year of the project. Information from the lay counselor record form maintained in the patient chart will also be extracted including number of visits, length of visits, and topics discussed. The project staff will complete this form for intervention and comparison clinics.

**HIV Prevention Interventions**

Overview:

This prevention intervention package is designed to integrate HIV prevention into routine care of HIV-positive patients. During the course of standard care, discussions between health care providers and HIV-positive patients about HIV prevention-related topics (e.g., reducing risky sexual behavior, disclosing HIV status, partners’ HIV testing) often do not occur. Consequently many HIV-positive individuals may unknowingly continue to engage in behaviors that put their sex partners and future children at risk for acquiring HIV. In addition, preventive services such as family planning and managing sexually transmitted infections (STIs) are often not delivered during routine HIV clinic visits, leaving further missed opportunities to reduce transmission of HIV.

Intervention clinic sites will implement the package of prevention interventions and deliver the interventions to all HIV-positive patients during routine clinic visits as part of standard care (see Figure 1). All HIV health care providers, nurses, and other relevant staff will be trained to implement the prevention interventions in the HIV care and treatment clinics. The package of HIV prevention interventions includes provider-delivered prevention messages, assessment and treatment of STIs, and provision of basic family planning counseling and services. Specifically, over the course of regular clinic visits, providers will ask about key prevention issues (e.g., partner testing, disclosure, condom use), assess for symptoms of STIs, and assess pregnancy status or intentions of women (or status/intentions of partners of men). Providers will treat STIs as indicated and provide basic family planning counseling or services as needed in the HIV clinic setting. Health care providers will also screen patients and determine if they need additional HIV prevention or adherence counseling. If they do, providers will complete a referral card with the topic(s) for counseling identified and refer the patients to lay counselors for the additional HIV prevention and/or adherence services following their visit with the provider.

Lay counselors will be persons without medical training, many of whom will be people living with HIV, who will be trained to provide counseling and services to HIV-positive patients in HIV care and treatment settings. They will be hired as project staff in the intervention clinics and located in the HIV clinic setting. They will provide HIV prevention and ART adherence counseling to HIV-positive patients and will counsel patients on issues such as disclosure of HIV status to partners, partner testing, adherence to medications, and alcohol reduction. Patients will be seen by a counselor in conjunction with their regularly scheduled clinic visit, and will be encouraged to be seen for additional counseling visits between clinic visits as needed. Lay counselors will also conduct HIV testing of partners in the HIV care and treatment clinics where permitted by country policy.

**Five HIV Prevention**

**Steps for PLWHA**

**HIV Prevention Messages for PLWHA**

**Counselors**

**HIV Prevention and Adherence Counseling for PLWHA**

**Doctors**

**Clinical Officers**

**Nurses**

**Family Planning and Safer Pregnancy Counseling for PLWHA**

Intervention Tools

Intervention Implementers

**Doctors**

**Clinical Officers**

**Nurses**

**Pharmacists**

**Social Workers**

**Figure 1. HIV prevention intervention package components and staff who will be implementing the intervention components.**

In comparison clinics, health care providers will continue providing the usual standard of care for patients and if counselors are available in each country, they will continue with the standard services that they provide. After completion of 12 month data collection in comparison sites, the health care providers will be provided with the opportunity to receive all aspects of the health care provider training. If counselors are present in the comparison clinics they will be provided with the opportunity to receive the complete lay counselor training.

Over the 12-month evaluation period, patients in the study clinics will be seen by their providers approximately every month for those who have recently started ART and every 2-3 months for those who have been on ART for a longer amount of time. For those not receiving ART, the current standard of care is clinic visits every 2-3 months. Thus, it is expected that participants who are on ART will receive the prevention intervention between 4 and 12 times and those not on ART will receive the prevention intervention approximately 4 times over the course of the project (depending on the number of times they come to the clinic). However, many patients not on ART may come for clinic visits less frequently which may result in less exposure to the intervention.

***Health Care Provider-delivered Prevention Messages***

*1) 5 HIV Prevention Steps for PLWHA in HIV Care and Treatment Settings*

and

*HIV Prevention Messages for PLWHA in HIV Care and Treatment Settings*

Background: Delivering prevention messages to HIV-positive individuals in care and treatment settings can significantly impact the HIV transmission risk behavior of infected persons. Two US-based health care provider-delivered interventions involving providers discussing prevention topics (e.g., serostatus disclosure, consistent condom use) were found to be effective at decreasing the rate of unprotected sex in HIV-positive individuals (Fisher et al., 2006; Richardson et al., 2004). In addition, these studies showed that integrating brief prevention discussions into routine clinic visits in busy medical settings was feasible. Training health care providers to routinely deliver prevention messages to HIV-positive patients in care and treatment settings in sub-Saharan African countries may also be an effective prevention strategy to facilitate reduction of sexual risk behaviors. The intervention and materials developed for this project were adapted from Partnership for Health (Richardson et al., 2004). The investigators obtained input from health care providers in Kenya, Uganda, and Botswana for the adaptation of the materials for the African context and field tested the interventions in 3 clinics in Kenya. The original intervention was expanded to include additional prevention messages and services to more comprehensively address prevention for HIV positive persons and to meet the demands of the African setting.

Intervention Components:

HIV health care providers (e.g., doctors, clinical officers, nurses) and professional staff (e.g., social workers, pharmacists) who are involved with the care of HIV infected patients in the HIV clinic will assess prevention issues and deliver specific prevention messages during routine clinic visits. Specifically, providers will do the following:

- Assess whether patient is sexually active
- Assess whether sex partner(s) have been tested for HIV and refer partner(s) for testing
- Assess whether patient has disclosed to sex partner(s) and encourage disclosure
- Encourage adoption of safer sex behaviors (e.g., abstain or be faithful to one partner, reduce number of partners, and use condoms with every sexual encounter)
- Explain the negative consequences of having sex without a condom
- Assess for alcohol use and refer for alcohol counseling (if indicated)
- Assess adherence to ARVs and refer for adherence counseling (if needed)
- Provide condoms
- Refer to lay counselor for in-depth HIV prevention counseling when indicated or desired

Health care providers will communicate these messages over the course of a clinical visit; however, providers may not communicate all messages to a patient during a single visit given time constraints. Providers will be trained to prioritize messages based on patients’ circumstances and they will raise all of these critical issues over the course of their patients’ regular care. In addition, they will encourage patients to follow up with the lay counselors for more in-depth discussions on these topics.

Training: Health care providers, social workers, and pharmacists will attend a 2 day training on integrating prevention into HIV care and treatment settings. Attendees will be trained on the critical role prevention efforts with HIV-positive persons can play in slowing the spread of HIV in their communities, and the importance of integrating prevention for people living with HIV into care and treatment settings. They will be trained to assess prevention issues and deliver prevention messages during regular clinic visits.

Providers and staff will be provided with two different job aids that can be used for delivering the prevention interventions to patients. One job aid is *5 HIV Prevention Steps for PLWHA in HIV Care and Treatment Settings*, which is a one page provider card that summarizes the key behavioral messages and prevention services HIV-positive patients should receive at *every* visit as part of routine care. The second job aid is *HIV Prevention Messages for PLWHA in HIV Care and Treatment Settings,* which is a scripted and illustrated flip chart that clinic staff can use to communicate prevention messages that are tailored to the person’s specific circumstances and partner’s status.

All providers will be trained to use the provider card and to address the 5 prevention steps with every patient at every visit (i.e., (1) give prevention recommendations, (2) assess adherence, (3) assess for STIs and treat according to national guidelines, (4) assess pregnancy status and intentions, (5) give condoms). The provider card summarizes the key prevention messages (disclosure, partner testing, risk reduction strategies) and services (STI management, family planning, condoms) and allows providers to discuss these issues with patients in their own words during the course of their visit. For providers who prefer to use a scripted job aid or prefer illustrations for teaching with patients, the flip chart can also be used to deliver prevention messages. The prevention messages are the same on both tools. Our experience has shown that most providers prefer to use the provider card; however, the flip chart is a very important component of skills building for providers during the training.

Providers and clinic staff will also be given supportive materials reinforcing these messages, including exam room and waiting area posters and patient handouts. They will also be trained to refer to lay counselors for in-depth discussions on the intervention topics and for assistance overcoming barriers to preventive behaviors (e.g., disclosure, condom use).

It is anticipated that the increased assessment of STIs will increase the demand for STI treatment. To ensure the ethical treatment of participants syndromically diagnosed with STIs, the study will assist the intervention clinics with ensuring that adequate supplies of STI drugs (following national guidelines) are available.

Materials:

- One page job aid with prevention interventions and messages for providers and staff to deliver during routine clinic visits
- Flip chart with scripted with prevention messages for providers to deliver during routine clinic visits
- Clinic waiting area posters with prevention messages for patients
- Clinic exam room posters reminding providers to talk to their patients about prevention
- Patient handouts on correct condom use and prevention (e.g., disclosure, partner testing, condom use)
- Participant Manual used during training and which includes resource information

*2) Family Planning and Safer Pregnancy Counseling for PLWHA*

Background: There is a large unmet need for family planning services among HIV-positive women. Most HIV care and treatment clinics do not offer integrated family planning services but refer women to family planning clinics. These clinics are often not equipped to meet the specific needs of HIV-positive women, or may not know the HIV status of the women they are seeing, as many HIV positive women will not disclose their status in another setting. Further, referrals to additional clinics with long queues and waiting times are often deterrents to HIV-positive women who may already be spending a day a month at the HIV clinic for their care and treatment. However, as health is improved due to ARV treatment, many HIV-positive women and their spouses or partners resume sexual activity and have unintended pregnancies, which carry the risk of giving birth to children who are HIV-positive. Alternatively, many couples living with HIV desire children. Counseling on safer pregnancies for women living with HIV and their partners is critical to reduce the chances of transmission to infants. The intervention and materials used in this project were adapted from WHO’s IMAI Reproductive Choices and Family Planning for people living with HIV (WHO, 2006b) in collaboration with WHO. These materials were adapted for the HIV care and treatment setting.

Intervention: HIV providers will ask female patients about their pregnancy status and pregnancy intentions during every routine clinic visit (or the status and intentions of their male patients’ partners). Providers will tailor the counseling and services they offer to the patient’s particular situation. Specifically,

- HIV-positive women who do not desire pregnancy in the near future will be counseled by the provider on dual protection and dual method use and provided with condoms and contraceptive pills or injections, if desired. Providers will refer women who desire longer-acting or more permanent forms of birth control (i.e., IUD, sterilization) to the family planning clinic.
- HIV-positive women who desire pregnancy will be informed by their provider of the risks involved and on safer ways to conceive, with particular emphasis on using condoms except for the most fertile days of the menstrual cycle. In addition, women’s health status and medication regimen will be assessed, and brief guidance on safer pregnancy and delivery to reduce the likelihood of maternal-to-child transmission will be provided.
- HIV-positive women who are pregnant will be given brief guidance on HIV-related issues for their pregnancy and referred to antenatal care and a Prevention of Mother-to-Child Transmission (PMTCT) Clinic.
- Male spouses or partners will be asked about their and their partner’s pregnancy intentions and counseled or referred accordingly.

Training: Health care providers, nurses, and other relevant staff will participate in a 2-day training on integration of basic family planning counseling and services into routine HIV care. They will receive training on the important role family planning (contraceptives and counseling on safer pregnancies) plays in the reduction of maternal-to-child transmission of HIV. Providers will be trained to assess pregnancy status and intentions every visit and will be trained to assess eligibility and provide contraceptives (pills, injections) to women who do not desire pregnancy (and who desire contraceptives). Additionally, they will be trained to assess health and medication issues for HIV-positive women considering pregnancy, and to provide brief guidance on safer conception, pregnancy and delivery. Finally, they will be provided with a job aide containing guidance and resource information on the above topics, and they will be trained on how to use this tool during their clinic visits with patients.

Materials:

- Flipchart for providers with information for assessing eligibility for oral and injectable contraceptives, safer pregnancy strategies for HIV-positive women desiring children, and guidance for HIV-positive pregnant women.
- Participant Manual from training with referral information.

*3) HIV Prevention and Adherence Counseling by Lay Counselors*

Background: Many HIV-positive patients may need additional counseling to reinforce prevention messages and address barriers to changing high risk behavior. Given high patient volume and limited number of health care staff, health care providers face significant time constraints and are therefore unlikely to spend much time discussing prevention issues in-depth with patients. Training lay persons, most of whom are HIV-positive, to provide prevention counseling to patients in HIV clinics may be a cost-effective and supportive solution to addressing the current limitations in health facilities. Lay counselors will be persons without medical training who will be trained to provide counseling and services to patients in the HIV care and treatment setting. The intervention and materials for this project have been developed in collaboration with WHO and will be used by lay counselors in HIV care and treatment settings for this project, as well as primary care settings as part of the IMAI training.

Intervention: As part of the prevention intervention package, lay counselors (many of whom will be HIV-positive) will be hired and placed in the clinics to provide HIV prevention and ARV medication adherence counseling, group education sessions, and HIV testing to patients and their partners. Health care providers in the clinic will screen patients on prevention issues and medication adherence then refer those who need prevention or adherence counseling to lay counselors. Providers will complete a referral card and check the most important issue for the patient to address with the lay counselor (e.g., disclosure, getting partner tested, adherence, etc.). After completing the visit with the health care provider, patients will meet with the lay counselor for approximately 30 minutes. Patients will also meet with the counselor as needed for additional counseling between clinic visits. Lay counselors will use standardized screening questions to determine the most important topics to address with patients. Topics lay counselors may address in individual counseling sessions will include:

- Prevention of sexual transmission (partner disclosure, partner testing, partner reduction, correct and consistent condom use)
- Adherence to medication regimens
- Reduction of alcohol use

Lay counselors will also work with couples on the following:

- Partner testing, if not already tested
- Counselor-assisted disclosure if needed
- Counseling discordant couples on risk reduction strategies such as negotiating condom use, correct and consistent condom use, and supporting each other with health-related issues (e.g., adherence, alcohol use).

Using the 5As model adapted from the WHO IMAI training (WHO, 2004), prevention counselors will be trained to “assess, advise, agree, assist, and arrange” during the counseling sessions. For example, the lay counselor will *assess* the nature of the high risk behavior and offer information and make recommendations to the patient (*advise*). The lay counselor and patient will *agree* together on a behavior to change. The lay counselor will *assist* the patient in developing behavior change steps and resolving potential barriers to successfully changing the identified behavior. Finally, the lay counselor will *arrange* or organize any components necessary to help the patient meet the goal, as well as arrange another visit to continue addressing these issues.

In addition, where the local government allows, lay counselors will administer rapid HIV tests to sex partners of HIV-positive patients. The lay counselor will give each patient a slip to give to their partner if their partner has not been tested or needs to be re-tested. This slip encourages the partner to get tested for HIV and indicates where this can be done. Patients will be told that the contact slips should be brought back to the clinic when the partner comes in for testing in order to track partner testing. The counselor will refer any partner who tests HIV-positive immediately to the HIV clinic for care and treatment.

Lay counselors will also be trained to conduct group education sessions in the clinics and support groups (where appropriate). These educational sessions will deliver key prevention and healthy living messages to HIV patients who may not be seen by counselors for individual counseling sessions. Topics covered in these educational and/or group sessions will include:

- Basics of HIV
- Treatment of HIV and Adherence
- Prevention of Sexual Transmission of HIV
- Prevention of transmission to unborn children (PMTCT, family planning)
- Healthy Living (prevention of opportunistic infections, safe water and food, personal hygiene, coping and emotional support)
- Nutrition and exercise

Group education sessions will last approximately 45 minutes, and will include presentation and discussion of the information and a question and answer session. The lay counselor will use a flip chart designed with illustrations for patients and questions and text on the lay counselor side. The education flip chart also will be used if needed during individual counseling sessions to explain basic information about HIV/AIDS that a patient needs to understand to be able to successfully address a particular counseling issue. These sessions will be conducted in the waiting area of the clinic. Patients attending the clinic that day will be able to participate in these sessions. These sessions are typically conducted by nursing staff in clinics. Having the lay counselors conduct these sessions will allow nurses to perform other duties in the clinic.

Training: Lay counselors will participate in a 4-week training to learn skills in prevention-related counseling and rapid testing and counseling (where permitted) that will make them valuable members of the HIV clinic team. The first two weeks will include training on the importance of prevention and healthy living for persons living with HIV, and the role of the care and treatment clinic for providing prevention information and support. They will be trained to assess patients using standardized screening questions and to help patients address the most important prevention topics for their situation. Counselors will be trained to deliver prevention counseling to patients using the 5As approach described previously. They will be trained to counsel couples in addition to individuals, when indicated or desired. Counselors will also learn how to deliver a brief alcohol intervention for patients who use alcohol regularly. Also, counselors will be trained to conduct group education sessions on the basics of HIV prevention, HIV treatment and adherence, and positive living.

During the next two weeks of training they will be trained to conduct rapid testing on partners and to provide post-test counseling to both (where permitted by country guidelines). The training will include didactic presentations, skills-building exercises, and interactive role-plays to ensure skill acquisition.

Materials:

- Workbook with individual counseling strategies outlined for each prevention topic
- Flip Charts with educational materials for group educational sessions
- Clinic waiting area posters with prevention messages for patients
- Patient handouts on prevention topics
- Participant Manual from training with resource information

**Outcomes and minimum meaningful differences**

We anticipate enrolling 200 patients per clinic with 9 clinics per condition (3 per condition per country), for a total of 18 clinics and approximately 3600 patients (at least 2520 retained at 12 months). Power calculations demonstrate that this number of participants will allow us at least 80% power to detect a 25% relative difference between conditions for the primary outcome variable of any unprotected sex during the past 3 months, allowing for up to 30% loss to follow-up.

**Training for all study personnel**

The design of this project requires that the project staff at each study site have clear, defined roles and work cooperatively. Figure 2 below shows a sample country project staffing diagram and the staff duties are discussed in the text that follows.

**Figure 2. Sample country staffing diagram.**

**Country Project Team / Investigators**

**Site Evaluation Coordinator**

**(1 per project site,**

**6 total)**

**Country Project Coordinator**

**(n = 1)**

**Interviewers**

**(2 per project site,**

**12 total)**

**Health Care**

**Providers**

**(Clinic staff, not study staff)**

**Lay Counselors**

**(2-4 per project site,**

**12-24 total)**

**Data Entry Staff**

**(n = 1-2)**

**Site Program Coordinator**

**(1 per intervention site, 3 total**

**6 total))**

*Roles and responsibilities of project staff*

There will be one Country Project Coordinator for each of the countries who will oversee operations at all of the sites in their country. They will visit each participating clinic site at least once per month and troubleshoot and monitor project progress as needed. They will supervise Site Evaluation Coordinators and also manage quality control for data collection. The Country Project Coordinator will provide in-country technical assistance for study data collection issues to the clinic. In addition, the Country Project Coordinator will ensure that data from each site are sent to a central location and will supervise data entry staff, who are responsible for entry of all data from all the clinics in their country. Once data are entered and checked, the data entry staff will transmit the data using the Secure Data Network to CDC-Atlanta weekly..

The Site Evaluation Coordinator will oversee all study evaluation activities at the clinic site. They will supervise data collected by interviewers, ensure interviewers meet responsibilities and goals, collect data from health care provider and lay counselor observations, extract data from patient charts, gather information on clinic services provided, serve as a backup interviewer, and will be responsible for all study and data collection deadlines.

The Site Program Coordinator will be an existing full-time clinic employee (e.g., a nurse or matron, if possible) with approximately 25-50% of their time dedicated to this project. They will oversee implementation of the intervention package by health care workers and counselors at their respective clinic, supervise counselors (where appropriate) to ensure responsibilities are being conducted according to project implementation guidelines, maintain commodities and other needed supplies for project implementation, and assist in record keeping and clinic level data collection. In addition, the Site Program Coordinator will be the primary contact at the clinic for program-related intervention issues; communicate with clinic administration, clinic staff, project coordinator and study team regarding project-related issues; and ask about, address, and report all adverse events associated with the intervention package to the Country Project Coordinator and CDC-Atlanta immediately.

Counselors will be hired by the project to work as part of the clinical team in the HIV clinic. Their main duties will be to conduct group education sessions, individual and couple counseling with HIV-infected clinic patients, and HIV counseling and testing (where allowed by national guidelines) with partners of clinic patients. Also, they will communicate with health care providers and other clinic staff as needed about patient care and referrals and discuss any intervention concerns or patient issues with the Site Program Coordinator.

The project interviewers will collect data from patients, health care providers, and lay counselors. The interviewers will ensure that data collection activities adhere to the study protocol, that the data collected are complete and of the highest possible quality, and that all activities are conducted in a professional manner. Interviewers will complete participant informed consent, conduct patient interviews, administer questionnaires to health care providers and lay counselors, and assist the site evaluation coordinator at the clinic with other parts of the study including following-up and tracking study participants.

*Training for project staff*

Interviewers will receive thorough structured training from project staff on all the data collection forms and procedures. Training agenda items include purpose and scientific objectives of the project, interviewer responsibilities, locating and contacting participants, participant’s rights and informed consent, and confidentiality requirements. The hands-on training will include an introduction to data collection forms and procedures, working through several mock interviews, and completing administrative forms and reporting work progress. Training for interviewers is expected to take approximately 16 hours per interviewer and an additional 8 hours is estimated for follow-up training.

The bulk of the hands-on training, and the most crucial component, will be the mock interviews, and project staff will act as both the trainer and the mock respondent. The trainer will take the interviewers through each step of the interviewing process, from enrolling participants to ending the interview and completing any necessary forms. Within the interview itself, the trainer will demonstrate both the interviewing task being required of them as well as the response task being required of the project participant. Question-by-question instructions and the use of any visual or recall aids will be reviewed.

The trainer will also ask questions of the interviewer or intentionally make mistakes similar to those the interviewers may encounter in a real interview thus requiring the interviewer to learn how to handle unusual circumstances. Also, interviewers will be trained to refer that day to clinic and community counselors and health care providers any patient who displays obvious signs or endorses significant experience of depression or alcohol abuse. However, the project interviewers are not trained mental health professionals and the questionnaires are not diagnostic tools; thus, interviewers will not be responsible for providing any psychological services to patients or for articulating specific concerns or diagnostic impressions related to the referral.

The multilingual interviewers will work though additional mock interviewers in other languages to become familiar with the non-English versions of all the assessments and to discuss and resolve any issues specific to interviewing project participants at their site in languages other than English.

In addition to this type of mock interview, the interviewers will be paired together with each taking a turn at interviewing their partner. This method more closely resembles an actual interview and the performance of each interviewer can be more carefully monitored as the trainers walk around to observe and provide individual instruction.

During this training, the trainers will have ample opportunity, particularly through mock interviews, to work directly with each interviewer. In the context of these interactions, trainers will qualitatively ascertain the interviewer’s skills. At the end of training, any interviewer the trainers or other project staff believe may have difficulty with the project interviewer tasks be asked by project staff to not continue work with the project.

Interviewers will receive a Field Operations Procedures Manual which will serve as a procedural guide during actual data collection.

**PROCEDURES/METHODS: DATA HANDLING AND ANALYSIS**

**Data analysis plan**

The proposed project will be a multi-site nested cohort group-randomized trial (GRT) with a-priori matching at the clinic level. Here, group refers to clinic, and “study condition” or “condition” will refer to intervention vs. control assignment. Matching of clinics will be performed in the design of the project to improve balance across study conditions on factors potentially related to the outcome variables. However, the matching will not be reflected in the analysis, as studies have shown that doing so often reduces power in smaller GRTs due to the loss of degrees of freedom (Diehr et al., 1995). Site (country) will be reflected in the analysis, both as an interaction with intervention effect to test for a differential intervention effect by site, and as a main effect to adjust for pre-intervention differences in the outcome variables of interest. If interactions between site and the intervention effect are statistically significant, we will explore intervention effects within country. However, power will be low for this test as the sample sizes within countries are small.

This project presents a number of challenges that must be addressed in the primary and secondary analyses. First, the analytic approach must take into account the correlation among patients within clinics and multiple measurements on the same participants within the same groups over the course of the project. Second, as with any longitudinal cohort study, there is potential for attrition over the course of the study and differential attrition between intervention and comparison conditions. Finally, many of the outcome measures used to assess the impact of the intervention will be non-normally distributed, including dichotomous indicators for whether risky sexual behaviors occurred and frequency variables indicating how many partners a participant reported.

Prior to beginning analyses to evaluate intervention effects, preliminary analyses will be conducted to determine whether randomization was successful in creating equivalent groups of participants across study conditions at baseline. Any differences between intervention and comparison conditions at baseline will be controlled for in subsequent evaluations of the intervention effect. Analysis will also be conducted for the purpose of describing the baseline sample and examining the distributions of each of the outcome variables. Further analyses will explore distributional assumptions related to each outcome measure.

For the main outcome analysis, numerous options are available for the test of the effectiveness of the intervention. Mixed-model regression methods are often used for the analysis of GRTs and easily accommodate the over-time correlation at the group and member level, as well as the correlation of observations within groups. Using different procedures within SAS, such as PROC MIXED, PROC GLIMMIX, and PROC NLMIXED, mixed-model regression can be performed for many common statistical distributions, and some less-common ones often used in HIV research (e.g., censored log-normal for viral load measurements). We propose to use mixed-model regression methods to test for a difference between study conditions at 6 and 12 months, incorporating the baseline measurement as a covariate. Initial models will include fixed effects for the baseline value of the outcome variable, country, study condition, time (6 or 12 month measurement), the country by study condition interaction, the time by study condition interaction, the time by country interaction and the three-way interaction between time, study condition and country. Models will also include random effects for group and group by time. If the country by time by condition interaction is significant, the time by condition interaction will be tested stratified by country to determine the pattern of intervention effects for each country separately. Because power is based on the number of clinics, however, tests for country differences will have low power relative to the overall test for an intervention effect. If the three-way interaction is not significant, it will be dropped from the model and further evaluation of intervention effects will focus on the time by condition term. If this term is significant, condition effects will be evaluated stratified by timepoint; if it is not significant, a pooled estimate of the intervention effects (across timepoints) will be computed. This effect estimate has the advantage of greater power than a test at either timepoint alone, due to the larger number of observations. The random effects for group and time by group will control for the correlation within participant over time in these models. Also, the country by condition effect will be tested to determine whether the study condition effect (pooled across time) differs by country.

In addition to the approach we propose, a variety of other analytic approaches have been used for GRTs, including permutation tests, generalized estimating equations (GEE), and random coefficients models. Permutation tests involve computing a summary statistic for the differences between conditions and comparing this summary statistic to a distribution of all possible such statistics given the design of the study. We chose not to employ this method because, with three timepoints, we would either need to do two separate permutation tests with an alpha correction for multiple hypothesis tests, or do a permutation test on the group-specific slopes over time. The first approach would likely be less powerful than the one we chose, and the second approach would express intervention effects as linear terms, and the approach we chose allows for a nonlinear pattern of intervention effects over time. We avoided using a GEE approach because several simulation studies have shown GEE to yield an inflated Type I error rate in GRTs with fewer than 20 groups per condition (Murray, Hannan, & Baker, 1996; Feng, McLerran, & Grizzle, 1996). We also chose not to employ a random coefficients model approach because intervention effects are specified as linear trends in the random coefficients model and we have little prior evidence on which to base assumptions about the pattern of intervention effects.

The main outcome analysis will use an intent-to-treat approach (i.e., each participant will be analyzed in the study condition to which he or she was randomly assigned). Secondary analyses will explore the impact of actual intervention received, which will vary across participants. These analyses will need to control for or stratify by participant ARV status as those on ARVs will likely have more contact with providers.

Following the analysis of the observed data, we will repeat the primary outcome analysis using multiple imputation to address the potential for bias due to missing data. Multiple imputation ‘fills in’ the missing data using the association between variables in the dataset, and reflects the uncertainty in the imputed values by basing variance estimates on between- and within-imputation variability. This project is likely to have a moderate amount of missing data, and ignoring this missingness may lead to bias in the estimates of the intervention effect.

Tables 3 and 4 below provide sample table shells for the outcome analyses.

Table 3. Sample outcome analysis table shell indicating percentages for primary outcome variable by study condition and data collection period.

|  | Baseline  N (%) | | 6-Month Follow-up  N (%) | | 12-Month Follow-up  N (%) | |
| --- | --- | --- | --- | --- | --- | --- |
| Variable | Intervention  (n=) | Comparison  (n=) | Intervention  (n=) | Comparison  (n=) | Intervention  (n=) | Comparison  (n=) |
| Any unprotected sex past 3 months |  |  |  |  |  |  |
|  |  |  |  |  |  |  |
|  |  |  |  |  |  |  |
|  |  |  |  |  |  |  |
|  |  |  |  |  |  |  |
|  |  |  |  |  |  |  |
|  |  |  |  |  |  |  |
|  |  |  |  |  |  |  |

Table 4. Sample outcome analysis table shell indicating odds ratios and 95% confidence intervals for primary outcome variable by study condition and data collection period.

|  | Baseline  OR (95% CI) | | 6-Month Follow-up  OR (95% CI) | | 12-Month Follow-up  OR (95% CI) | |  |  |
| --- | --- | --- | --- | --- | --- | --- | --- | --- |
| Variable | Intervention | Comparison | Intervention | Comparison | Intervention | Comparison | χ2 | p-value |
| Any unprotected sex past 3 months |  |  |  |  |  |  |  |  |
|  |  |  |  |  |  |  |  |  |
|  |  |  |  |  |  |  |  |  |
|  |  |  |  |  |  |  |  |  |
|  |  |  |  |  |  |  |  |  |
|  |  |  |  |  |  |  |  |  |
|  |  |  |  |  |  |  |  |  |

**Data collection**

Pilot study

Prior to implementing the project a pilot study will be conducted with approximately 20 clinic patients in each country. The purpose of this pilot will be to determine the length of time to complete the patient questionnaire and determine whether there are items or procedures that need to be improved prior to full project implementation. Participants will be recruited from patients who come into the clinic during the week of the pilot study. A single clinic in each country will serve as the pilot study site. These clinics will be different sites than the intervention and comparison clinics in order to maintain the eligible sample of participants attending the study clinics. The pilot study sites are Katatura Hospital (Namibia), Ocean Road Cancer Institute (Tanzania), Gatundu District Hospital (Kenya).

Potential participants will resemble the project participants as much as possible with regard to gender, length of time attending clinic, and whether they are on ARVs. An interviewer will read potential participants a consent form (Appendix A), ask if they have any questions, and ask them to sign the consent. The interviewer will then proceed with administering the questionnaire. After each section the interviewer will ask the participant some questions (Appendix B) related to the length of the section and whether items were confusing (and if so, which items). Participants will then be thanked for their time and offered a soda or other drink. No additional reimbursement will be provided, as is the custom in the participating countries. Data from the pilot study will be used to inform any needed revisions to the procedures or measures. Once that information has been used to inform the main study, the pilot data will be destroyed. No pilot data will be used in the main study analysis.

Contact information

The contact information form will be collected immediately after the participant completes the consent form to participate in the study. This form will include information on how to best contact the participant, including where they stay, phone numbers, and what is the appropriate mechanism to get in touch with them in order to respect the confidentiality of their study participation. This form will remain in their file and the contact information will be updated during the 6-month follow-up interview.

Patient Questionnaire

All clinic patient study participants will complete an interviewer-administered questionnaire at 3 time points: baseline (enrollment), 6-month, and 12-month follow up (see Figure 2 below for Study flow diagram for clinic patients). All interviews will take place at the HIV clinics after the participants’ regularly scheduled clinic visit whenever possible. All interviews will take place in a private area of the HIV clinic, and the interview will last approximately one hour. Each participant will be individually interviewed by the project interviewer, who will be part of the research team and not a staff member of the clinic. Upon completion, the participant will be offered a soda or other drink and reimbursed for transportation costs to the clinic. No additional reimbursement will be provided, as is the custom in the participating countries. Participants will be encouraged to attend all regular clinic visits as scheduled by their health care provider over the course of the project.

Participants will be asked to provide contact information (see Appendix G) during the baseline interview. They will be asked the best method for contacting them (e.g., text message, phone call, in-person visit, mail/post) for follow-up visit reminders. Approximately one week before their 6- and 12 month follow-up visits, participants will be reminded of their appointments via their preferred method of being contacted. As much as possible the 6- and 12-month visits will be coordinated with regular clinic visits.

Project enrollment will occur over of a 2-3 month period at both intervention and comparison clinics. Following completion of the baseline data collection, the intervention will be implemented at the intervention clinics. Because of the baseline data collection period, the 6-month follow-up will be scheduled for a time estimated to be 6-months after the intervention is integrated into the clinic, so that all intervention clinic participants will have an opportunity to be exposed to the intervention following staff training and implementation. To make the intervention and comparison clinic project procedures as similar as possible, the intervention and comparison clinic 6-month follow-up interviews will occur during the same time period. The same battery of questionnaires will be administered to participants at the 6- and 12-month follow-up interviews, with the exception of some of the sociodemographic questions and items asking about whether something had “ever” happened. At each follow up interview, participants will be offered a soda or other drink and reimbursed for travel costs to the clinic upon completion of the interviews.

**Intervention implemented**

Health care providers trained

Lay counselors trained

Integration of family planning and STI services

**Customary care provided**

Health care providers continue with current standard of care (and lay counselors, if already in place)

Patient receives prevention interventions during all routine clinic visits as follows:

From health care provider

Prevention messages

FP counseling & contraception

STI management

From lay counselor

Prevention & TX counseling

Partner HIV testing

Alcohol intervention

Patient receives current standard of care from health care provider during all routine clinic visits

**Baseline Data Collection**

Questionnaire administered

**Comparison Clinics**

**Intervention Clinics**

**Study Visit 3**

**Comparison clinics receive intervention materials and training**

Health care providers and lay counselors trained

Integration of family planning and STI services

**Study Visit 1**

**Recruitment & Screening**

Randomly selected for participation

Eligibility determined

Consent process

**HIV prevention continues as integrated standard of care in intervention clinics**

**12 Month Follow-up**

Patient completes study questionnaire

**12 Month Follow-up**

Patient completes study questionnaire

**6 Month Follow-up**

Patient completes study questionnaire

**6 Month Follow-up**

Patient completes study questionnaire

**Study Visit 2**

**Figure 2. Study flow diagram for clinic patients.**

Health Care Provider and Lay Counselor Assessment

Prior to attending the training for the interventions, health care providers and lay counselors in the intervention clinics will be asked to complete baseline questionnaires about the services they deliver in their clinic and their routine standard of care with patients (Appendix I). At 6- and 12-months following implementation of the interventions, they will be asked to complete questionnaires about the acceptability of the intervention messages, materials and protocol, as well as the feasibility of implementing these prevention interventions into HIV care and treatment settings (Appendix J). In addition, they will be asked about the fidelity with which they have been able to implement them.

Health care providers in the comparison clinics will also complete baseline questionnaires about the services they deliver in their clinic and their routine standard of care with patients. The questionnaires will be completed again at 6- and 12-months in order to determine whether there were any changes in types of services delivered. If counselors are also in place in the comparison clinics as usual standard of care then they will complete the lay counselor questionnaires.

Additionally, project staff will observe about 3% of provider clinic visits and lay counselor counseling sessions in both the intervention and comparison clinics. The project staff will approach the provider or lay counselor and request permission to observe a patient visit. The provider or lay counselor will ask the patient for permission to have their clinic visit or counseling session observed. If the patient agrees, he/she will sign a consent form (Appendix F) granting permission to be observed. No personal or identifying information will be obtained from the patient, and they may or may not be enrolled in this project, given that this is a clinic-based intervention. The project staff will complete an observation checklist during the visit documenting the HIV prevention interventions that were discussed or delivered during the visit.

Medical Chart Review and Clinic Records Review

Clinic Services: The Clinic Services Form (Appendix O) captures HIV care and services delivered as well as contraceptives and STI treatment medications dispensed to HIV-positive patients at the clinic level. This will be completed by the project coordinator monthly in all clinics. The project coordinator will gather this clinic-level information from clinic records and clinic administrators.

Medical Chart Review: After each participant’s follow-up visit the study interviewer or coordinator will use the patient’s medical chart to gather information such as the clinic visits since last extraction, clinical indicators of HIV (e.g., CD4 count, WHO classification), medications, partner’s HIV status, reproductive health history, STI treatment provided (see Appendix P).

**Information management and analysis software**

All completed questionnaires, forms, and data collection instruments will be stored in a locked space at each clinic. Data entry will be done in each country using a MS Access database created at CDC-Atlanta and provided to the Country Project Coordinator in each country. Data will be analyzed using the most recent version of SAS.

**Data entry, editing, and management**

Figure 3 provides a data collection and flow diagram. Interviewers will be responsible for ensuring that they complete the patient interview correctly and in its entirety (unless patients refuse questions) for every enrolled participant, as well as the health care provider and lay

Country Evaluation Coordinator

Site Coordinator

Data manager

CDC-Atlanta

Interviewers

Lay counselors

Health care providers

Patients

Questionnaire

Clinical Care

Integration of services

Integration of services

Chart abstraction

Observation

Observation

Lay counselors

Health care providers

Patients

**Figure 3. Data collection and data flow.**

1. Interviewers collect data from patients, health care providers, and lay counselors.

2. Site project coordinators collect medical chart and observation data.

3. Interviewers check data they collect for accuracy, completeness, and legibility then provide to site project coordinators. Site project coordinators review data documents and provide feedback to interviewers and request clarifications if necessary.

4. All data are provided to country evaluation coordinator who will review the data and request clarifications if necessary.

5. Country evaluation coordinator monitors and supervises data entry and checking within their country.

6. Country evaluation coordinator will provide the data to CDC-Atlanta, where they are all combined into a single data set for analysis. CDC-Atlanta will communicate with the country evaluation coordinators regarding any data issues.

counselor questionnaires. The site coordinator will be responsible for monitoring the interviewer’s data collection activities and checking data collection forms to ensure that all data are correctly collected from each patient, provider, and lay counselor. If data have not been

correctly collected, the site project coordinator will meet with the interviewer to resolve data discrepancies (where possible) or discuss how to ensure that the errors do not occur again. Once data are checked for completeness they will be sent via secure carrier to the country evaluation coordinator who will supervise data entry and checking, which will occur in each country.

Data entry will be done in a Microsoft Access database which will be created by CDC-Atlanta and distributed to each of the country project coordinators. Approximately 10% of the data will be double entered and compared for discrepancies. Once discrepancies are resolved and corrected, data will be sent encrypted electronically once a week to CDC-Atlanta where the data from the three countries will be compiled into a single database.

All participant data will be kept confidential from family, friends, and co-workers, as well as from non-project-related hospital staff, patient providers, and lay counselors. Also, provider and lay counselor data will follow the same procedures of confidentiality and will not be shared with clinic staff or supervisors. Privacy will be maintained by means of participant identification numbers; all persons participating in any aspect of the project (patients, providers, lay counselors) will get an identification number. The list linking numbers to names, as well as any other project data collected, will be stored in a safe, secure, locked location only available to the on-site project staff. Project data will have only a participant number (without linkages or identifiers to patient names) and will only be available to the CDC-Atlanta research team and in-country research teams. Any documents linking participants’ names and study identification numbers will be destroyed within three months of completion of the 12 month data collection. Due to the nature of the project funding, all project data will be owned by CDC-Atlanta. The Publications Committee (described below) will review data analysis proposals by the study team.

**Quality control/assurance**

Project coordinators and patient interviewers will be rigorously trained by CDC-Atlanta and in-country staff. The site coordinator will work with the site interviewers to clean and edit all completed data collection forms and prepare them for data entry. The site coordinator will also be responsible for providing immediate feedback to staff who collected incomplete information and will monitor the staff person’s future work.

A communication system will be in place whereby the CDC-Atlanta staff, project staff, and data entry staff will regularly discuss all aspects of data collection, participant interactions, and problems that may arise. Project coordinators and interviewers will collect the data and project coordinators will be responsible for ensuring data are correctly entered and checked and sending it to CDC-Atlanta on the appropriate, specified dates.

**Bias in data collection, measurement, and analysis**

Non-response bias associated with loss to follow-up is a potential source of bias. Patient participants will be receiving care and treatment at the clinic, thus will be likely to come for scheduled appointments. However, there will be some participants who will move away from the clinic area or drop out of care, as well as some deaths. We will attempt to minimize loss to follow-up by collecting methods of contacting participants to remind them of their appointments. We will also employ statistical methods to reduce the bias due to non-response, including multiple imputation.

**Intermediate reviews and analyses**

Baseline data will be analyzed and prepared for publication while the study is ongoing. The 6- and 12-month data will not be analyzed during the project to examine intervention effects. The data analysis programming will occur prior to the end of data collection in order to be able to test analysis code and have it prepared for analysis once the completed data are provided for analysis.

**Limitations of study**

Potential problems may arise, given that this is a large, multi-site project in a resource-limited setting; thus, alternate plans will be developed. Because attrition may be high, approximately 30% when following HIV-positive patients across one year, baseline sample size will be large to ensure statistical power to detect subtle behavior changes. Also, clinics are extremely busy with high patient volume, and providers may find incorporating the intervention into their routine clinic visits difficult. If all providers do not administer the intervention to all patients (i.e., some patients do not receive the intervention), measuring the effectiveness of the intervention will be difficult. Thus, fidelity measures (e.g., observation, questionnaires) are included in the project to ensure providers and lay counselors conduct the intervention as designed. In addition, the questionnaire data are all self-report, which is potentially subject to socially desirable responding, recall biases, or forgetting.

**PROCEDURES/METHODS: HANDLING OF UNEXPECTED**

**OR ADVERSE EVENTS**

**Response to new/unexpected findings, changes in study environment**

The project protocol is noninvasive and involves minimal to no risk to patients. However, all discussions, issues, and complaints related to the intervention package implementation and evaluation will be reviewed promptly to ensure close monitoring of the impact of the package on the patients, clinic staff, and project team. Any changes in the study environment or problems will immediately be reported to the local project team and discussed with the CDC-Atlanta team and the project Steering Committee. Appropriate action will be taken to resolve or deal with all issues accordingly.

**Identifying, managing, and reporting adverse events**

The project coordinators will be responsible for immediately reporting all unanticipated problems involving risks to subjects or others including adverse events to the in-country research team and CDC-Atlanta team. These unanticipated problems will be discussed, a verbal and/or written action plan will be devised, and an appointed person or agency will implement it within 24 hours of the issue occurring. The research team will maintain written documentation about the event, plan, and resolution. If deemed necessary, a report will be made to the IRB using the CDC’s Incident Report Form 1254 (Appendix Q).

**Emergency care**

There are no expected harmful consequences as a result of participating in this evaluation. However, should an HIV-positive patient express a need for emergent medical care, the project coordinator will attempt to locate immediate care through the health care facility where the patient is participating in the project.

**PROCEDURES/METHODS: DISSEMINATION, NOTIFICATION,**

**AND REPORTING OF RESULTS**

**Notifying participants of their individual results**

Patients, providers, and lay counselors will not receive feedback on their answers to questionnaires.

**Notifying participants of study findings**

Written material summarizing the findings from this project will be made available to patients and providers through the participating clinics upon completion of the project. Providers and lay counselors will be notified when published material is available for their reading.

**Anticipated products/inventions resulting from the study and their use**

It is anticipated that this project will yield several manuscripts, as well as provide data for presentations at conferences. Given the importance of integrating HIV prevention with HIV-positive persons into health care settings, results of this evaluation will be important to disseminate. In addition, the intervention materials, provider trainings, and counselor trainings will be refined (and revised, if indicated) based on this project and made available for dissemination as appropriate.

**Disseminating results to the public**

Data collected during the course of this study will be written into multiple manuscripts and submitted for peer-reviewed publication. Also, the research project team members will seek opportunities to disseminate the findings at professional conferences and meetings to facilitate implementation of the HIV prevention intervention package at other care and treatment settings across Sub-Saharan Africa.

**Publications Committee**

There will be a Publication Committee which will oversee authorship of all presentations and publications resulting from this study. CDC Headquarters will chair the Publications Committee and each participating implementing partner (ICAP-Kenya, ICAP-Tanzania, and Namibia Ministry of Health and Social Services) and CDC in-country teams (CDC-Kenya, CDC-Tanzania, and CDC-Namibia) will have a voting seat on the Publications Committee. A representative from the Office of Global AIDS Coordinator and a representative from the OGAC Prevention with Positives Task Force will also each hold a seat on the Publications Committee. All decisions related to the analysis of data and authorship of publications and presentations will be made by majority agreement of Publication Committee members.

**REFERENCES**

Antelman, G., Smith Fawzi, M. C., Kaaya, S., Mbwambo, J., Msamanga, G. I., Hunter, D. J., et al. (2001). Predictors of HIV-1 status disclosure: A prospective study among HIV-positive pregnant women in Dar Es Salaam, Tanzania. *AIDS, 15,* 1865-74.

Arthur, G., Nduba, V., Forsythe, S., Mutemi, R., Odhiambo, J., & Gilks, C. (2007). Behavior change in clients of health centre-based voluntary counselling and testing services in Kenya. *Sexually Transmitted Infections, 83*, 541-546.

Braithwaite, R. S., McGinnis, K. A., Conigliaro, J., Maisto, S. A., Crystal, S., Day, N. et al. (2005). A temporal and dose-response association between alcohol consumption and medication adherence among veterans in care. *Alcoholism: Clinical and Experimental Research, 29(7):1190-1197*.

Bunnell, R. (2007) [Integrating Prevention into Care and Treatment Settings](http://www.hivimplementers.org/agenda/pdf/N2/N2 Bunnell-Integrating Prevention into Care & Treatment Sett.ppt.pdf). *2007 HIV/AIDS Implementers Meeting,* June, 2007: Kigali, Rwanda.

Bunnell, R., Ekwaru, J. P., Solberg, P., Wamai, N., Bikaako-Kajura, W., Were, W. et al. (2006a). Changes in Sexual Behavior and Risk of HIV Transmission After Two Years of Antiretroviral Therapy and Prevention Interventions in Rural Uganda. *2006 HIV/AIDS Implementers Meeting,* June 12-15, 2006: Durban, South Africa.

Bunnell, R., Ekwaru, J.P., Solberg, P., Wamai, N., Bikaako-Kajura, W., Were, W. et al. (2006b). Changes in sexual behavior and risk of HIV transmission after antiretroviral therapy and prevention interventions in rural Uganda. *AIDS, 20,* 85-92.

Buve, A., Weiss, H. A., Laga, M., Van Dyck, E., Musonda, R., Zekeng, L., et al. (2001a). The epidemiology of gonorrhea, chlamydial infection and syphilis in four Africa cities. *AIDS, 15 (suppl 4),* S79-S88.

Buve, A., Weiss, H. A., Laga, M., Van Dyck, E., Musonda, R., Zekeng, L., et al. (2001b). The epidemiology of trichomoniasis in women in four Africa cities. *AIDS, 15 (suppl 4),* S89-S96.

Celum, C. et al. (2009). Twice-daily acyclovir to reduce HIV-1 transmission from HIV-1 / HSV-2 co-infected persons within HIV-1 serodiscordant couples: a randomized, double-blind, placebo-controlled trial. Presented at *International AIDS Society Conference on HIV Pathogenesis, Treatment and Prevention*, July 19–22, 2009

Celum, C., Wald, A., Hughes, J., Sanchez, J., Reid, S., Delany-Moretlwe, S. et al. (2008). Effect of acyclovir on HIV-1 acquisition in herpes simplex virus 2 seropositive women and men who have sex with men: A randomized, double-blind, placebo-controlled trial. *Lancet, 371*, 2109-2119.

Centers for Disease Control and Prevention (2003). Incorporating HIV Prevention into the Medical Care of Persons Living with HIV. *MMWR Recommendations and Reports*, *52(RR12),* 1-24.

Central Bureau of Statistics (CBS) [Kenya], Ministry of Health (MOH) [Kenya], and ORC Macro. 2003. *Kenya Demographic and Health Survey 2003.* Calverton, Maryland: CBS, MOH, and ORC Macro.

Chesney, M. A., Ickovics, J. R., Chambers, D. B., Gifford, A. L., Neidig, J., Zwickl, B., et al. (2000). Self-reported adherence to antiretroviral medications among participants in HIV clinical trials: The AACTG Adherence Instruments. *AIDS Care, 12(3),* 255-266.

Cleland, J., & Ali, M. M. (2006). Sexual abstinence, contraception, and condom use by young African women: A secondary analysis of survey data. *The Lancet*, *368,* 1788-1793.

Cohen, M. S., Hoffman, I. S., Royce, R. A., Kazembe, P., Dyer, J. R., Daly, C. C. et al. (1997). Reduction of concentration of HIV-1 in semen after treatment of urethritis: implications for prevention of sexual transmission of HIV-1. AIDSCAP Malawi Research Group (1997). *Lancet, 349*, 1868-73.

Cook, R. L., McGinnis, K. A., Kraemer, K. L., Gordon, A. J., Conigliaro, J., Maisto, S. A. et al. (2006). Intoxication before intercourse and risky sexual behavior in male veterans with and without human immunodeficiency virus infection. *Medical Care, 44(8, Suppl 2),* S31-S36.

Dallabetta, G., & Neilsen G. (2005). Efforts to control sexually transmitted infections as a means to limit HIV transmission: What is the evidence? *Current Infectious Disease Reports, 7(1),* 79-84.

Desgrees-du-Lou, A., Msellati, P., Viho, I., Yao, A. D., Yapi, D., Kassi, P. et al. (2002). Contraceptive use, protected sexual intercourse and incidence of pregnancies among African HIV-positive women. DITRAME ANRS 049 Project, Abidjan 1995-2000. *International Journal of STD & AIDS*, *13(7),* 462-468.

Diehr, P., Martin, D.C., Koepsell, T., Cheadle, A. (1995). Breaking the matches in a paired t-test for community interventions when the number of pairs is small. *Statistics in Medicine, 14*, 1491-1504.

Feldblum, P.J., Kuyoh, M., Omari, M., Ryan, K.A., Bwayo, J.J., & Welsh, M. (2000). Baseline STD prevalence in a community intervention trial of the female condom in Kenya. *Sexually Transmitted Infections, 76*, 454-456.

Feng, Z., McLerran, D., Grizzle, J. (1996). A comparison of statistical methods for clustered data analysis with Gaussian error. *Statistics in Medicine, 15*, 1793-1806.

Fisher, J. D., Fisher, W. A., Cornman, D. H., Amico, R. K., Bryan, A., Friedland, G. H. et al. (2006). Clinician-Delivered intervention during routine clinical care reduces unprotected sexual behavior among HIV-positive patients. *Journal of Acquired Immune Deficiency Syndrome, 41(1),* 44-52.

Fleming, D. T., & Wasserheit, J. N. (1999). From epidemiological synergy to public health policy and practice: the contribution of other sexually transmitted diseases to sexual transmission of HIV infection. *Sexually Transmitted Infections, 75(1),* 3-17.

Fritz, K. E., Woelk, G. B., Bassett, M. T., McFarland, W. C., Routh, J. A., Tobaiwa, O. et al. (2002). The association between alcohol use, sexual risk behavior, and HIV infection among men attending beerhalls in Harare, Zimbabwe. *AIDS and Behavior, 6(3),* 221-228.

Gavin, S. R. & Cohen, M. S. (2004). The role of sexually transmitted disease in HIV transmission. *Nature Reviews: Microbiology, 2,* 33-42.

Gayle, H. & Lang J. (2004). Seizing the opportunity to capitalize on the growing access to HIV treatment to expand HIV prevention. *Lancet, 364,* 6-8.

Gregson S, Adamson S, Papaya S, Mundondo J, Nyamukapa CA, Mason PR, Garnett GP, Chandiwana SK, Foster G, Anderson RM. Impact and Process Evaluation of Integrated Community and Clinic-Based HIV-1 Control: A Cluster-Randomised Trial in Eastern Zimbabwe. *PLoS Med*. 2007 Mar 27;4(3):e102.

Halperin, D. & Epstein H. (2004). Concurrent sexual partnerships help to explain Africa’s HIV prevalence: Implications for prevention. *The Lancet, 364*(9428), 4-6.

Herbst, J. H., Sherba, R. T., Crepaz, N., DeLuca, J. B., Zohrabyan, L., Stall, R. D. et al. (2005). A meta-analytic review of HIV behavioral interventions for reducing sexual risk behavior of men who have sex with men. *Journal of Acquired Immune Deficiency Syndrome*, 39(2), 228-241.

Kamali A, Quigley M, Nakiyingi F, et al. (2003). Syndromic management of sexually-transmitted infections and behaviour change interventions on transmission of HIV-1 in rural Uganda: a community randomised trial. *Lancet*, 361:645–52.

Kalichman, S. C., Leickness, C. S., Kaufman, M., Cain, D., & Jooste, S. (2007a). Alcohol use and sexual risks for HIV/AIDS in sub-Saharan Africa: Systematic review of empirical findings. *Prevention Science, 8*, 141-151.

Kalichman, S. C., Simbayi, L. C., Vermaak, R., Cain, D., Jooste, S., & Peltzer K. (2007b). HIV/AIDS Risk Reduction Counseling for Alcohol Using Sexually Transmitted Infections Clinic Patients in Cape Town, South Africa. Journal of Acquired Immune Deficiency Syndromes: JAIDS, 44(5), 594-600.

Kangudie, M., Judmann, G., & van der Veen F. Factors associated with defaulting HAART in Rehoboth District Mission Hospital, Namibia: Programmatic challenges. *2006 HIV/AIDS Implementers Meeting,* June 12-15, 2006: Durban, South Africa.

Kapiga, S. H., & Lugalla, J. L. P. (2003). Male condom use in Tanzania: Results from a national survey. *East African Medical Journal*, *80(4),* 181-190.

Kapiga, S.H., Sam, N.E., Mlay, J., Aboud, S., Ballard, R.C., Shao, J.F., et al. (2006). The epidemiology of HIV-1 infection in northern Tanzania: Results from a community-based study. *AIDS Care, 18*, 379-387.

Kaul R, Kimani J, Nagelkerke NJ, et al. (2004). Monthly antibiotic chemoprophylaxis and incidence of sexually transmitted infections and HIV-1 infection in Kenyan sex workers: a randomized controlled trial. *The Journal of the American Medical Association*, 291:2555–2562.

Kiragu, K., Tanui, I., Baltazar, J., & Wanyungu, J. Alcohol use and HIV testing among health workers in Kenya. *2006 HIV/AIDS Implementers Meeting,* June 12-15, 2006: Durban, South Africa.

King-Spooner, S. (1999). HIV prevention and the positive population. *International Journal of STD & AIDS, 10*, 141 -150.

Lurie, M. N., Williams, B. G., Zuma, K., Mkaya-Mwamburi, D., Garnett, G. P., Sweat, M. D. et al. (2003). Who infects whom? HIV-1 concordance and discordance among migrant and non-migrant couples in South Africa. *AIDS* *17*, 2245-2252.

Magadi, M. A. (2003). Unplanned childrenbearing in Kenya: The socio-demographic correlates and the extent of repeatability among women. *Social Science & Medicine*, *56*, 167-178.

Mast, T.C., Kigozi G., Wabwire-mangen, F., Black, R., Sewankambo, N., Serwadda, D., Gray, R., Wawer, M., Wu, A.W. (2004). Measuring quality of life among HIV-infected women using a culturally adapted questionnaire in Rakai district, Uganda. *AIDS Care, 16*, 81-94.

McKenna, S. L., Muyinda, G. K., Roth, D., Mwali, M., Ng’andu, N., Myrick, A. et al. (1997). Rapid HIV testing and counseling for voluntary testing centers in Africa. *AIDS, 11(suppl1),* S103-S110.

Medley, A., Garcia-Moreno, C., McGill, S., & Maman, S. (2004). Rates, barriers and outcomes of HIV serostatus disclosure among women in developing countries: implications for prevention of mother-to-child transmission programmes. *Bulletin of the World Health Organization, 82*, 299-307.

Morris, M. & Kretzschmar, M. (1997). Concurrent partnerships and the spread of HIV. *AIDS, 11*, 641-48.

Mpangile, G., Praag, E., Sima, M., & Mmbaga, V. (2006). Sexual and child-bearing needs of people on ART: The forgotten agenda. HIV/AIDS Implementers’ Meeting, June 12-15, 2006. Durban, South Africa.

Murray, D. M. (1998). *Design and Analysis of Group-Randomized Trials*. New York: Oxford University Press.

Murray, D. M. & Blitstein, J. L. (2003). Methods to reduce the impact of intraclass correlation in group-randomized trials. *Evaluation review, 27*, 79-103.

Murray, D.M., Hannan, P.J., & Baker, W.L. (1996). A Monte Carlo study of alternative responses to intraclass correlation in community trials. Is it ever possible to avoid Cornfield’s penalties? *Evaluation Review, 20*, 313-337.

Mwaluko, G., Urassa M., Isingo, R., Zaba, B., & Boerma, J.T. (2003). Trends in HIV and sexual behavior in a longitudinal study in a rural population in Tanzania, 1994-2000. *AIDS, 17*, 2645-2651.

Nagot, N., Ouedraogo, A., Foulongne, V., Konate, I., Weiss, H. A., Vergne, L. et al., (2007). Reduction in HIV-1 RNA levels with therapy to suppress herpes simplex virus. *New England Journal of Medicine, 356,* 790-799.

Nakayiwa, S., Abang, B., Packel, L., Lifshay, J., Purcell, D. W., King, R. et al. (2006). Desire for children and pregnancy risk behavior among HIV-positive men and women in Uganda. *AIDS and Behavior, 10,* S95-S104.

National Institute on Alcohol Abuse and Alcoholism – NIAAA (2003). Task Force on Recommended Alcohol Questions - National Council on Alcohol Abuse and Alcoholism Recommended Sets of Alcohol Consumption Questions - October 15-16, 2003. Washington, DC.

Norman, L.R. (2003). Predictors of consistent condom use: a hierarchical analysis of adults from Kenya, Tanzania and Trinidad. *International Journal of STD & AIDS, 14*, 584-590.

Oladapo, O. T., Daniel, O. J., Odusoga, O. L., Ayoola-Sotubo, O. (2005). Fertility desires and intentions of HIV-positive patients at a suburban specialist center. *Journal of the National Medical Association, 97(12),* 1672-1681.

Pals, S.L., Beaty, B.L., Posner, S.F., & Bull, S.S. (2008). Unpublished manuscript.

President’s Emergency Plan for AIDS Relief – PEPFAR (2007). Technical considerations for the FY07 COPs: Prevention for HIV-positive individuals.

Pilcher, C. D., Tien, H. C., Eron, J. J., Vernazza, P. L., Leu, S-Y., Stewart, P. W. et al. (2004). Brief but efficient: Acute HIV infection and the sexual transmission of HIV. *Journal of Infectious Disease, 189*, 1785-92.

Radloff, L. S. (1977). The CES-D Scale: A self-report depression scale for research in the general population*. Applied Psychological Measurement, 1,* 385-401*.*

Richardson, J. L., Milam, J., McCutchan, A. Stoyanoff, S., Bolan, R., Weiss, J. et al. (2004). Effect of brief safer-sex counseling by medical providers to HIV-1 seropositive patients: a multi-clinic assessment. *AIDS*, *18*, 1179-1186.

Ross, D.A., Changalucha, J., Obasi, A.I.N., Todd, J., Plummer, M.L., Cleophas-Mazige, B., et al. (2007). Biological and behavioral impact of an adolescent sexual health intervention in Tanzania: a community-randomized trial. *AIDS, 21*, 1943-1955.

Schreibman, T., & Friedland, G. (2003). Human immunodeficiency virus infection prevention: Strategies for clinicians. *Clinical Infectious Diseases, 36(1),* 1171-1176.

Stanton, B.F., Fitzgerald, A.M., Li, X., Shipena, H., Ricardo, I.B., Galbraith, et al. (1999). HIV risk behaviors, intentions, and perceptions among Namibian youth as assessed by a theory-based questionnaire. *AIDS Education & Prevention, 11*, 132-149.

UNAIDS (2007). AIDS epidemic update: December 2007. Geneva, Switzerland.

Wasserheit, J. N. (1992). Epidemiological synergy. Interrelationships between human immunodeficiency virus infection and other sexually transmitted diseases. *Sex Transmitted Diseases, 19(2),* 61-77.

Watson-Jones, D., Mugeye, K., Mayaud, P., Ndeki, L., Todd, J., Mosha, F., et al. (2000). High prevalence of Trichomoniasis in rural men in Mwanza, Tanzania: results from a population based study. *Sexually Transmitted Infections,* *76,* 355-362.

Watson-Jones, D., Weiss, H. A., Rusizoka, M., Changalucha, J., Baisley, K. Mugeye, K. et al. (2008). Effect of herpes simplex on suppression on incidence of HIV among women in Tanzania. *The New England Journal of Medicine, 358*, 1560-1571.

Wawer MJ, Sewankambo NK, Serwadda D, et al. (1999). Control of sexually transmitted diseases for AIDS prevention in Uganda: a randomised community trial. *Lancet*, 353:525–35.

WHO/UNAIDS (2006). Consultation on STI interventions for preventing HIV: appraisal of the evidence. Geneva, Switzerland.

World Health Organization (2008). Essential Prevention and Care Interventions for Adults and Adolescents Living with HIV in Resource-Limited Settings. World Health Organization: Geneva, Switzerland.

World Health Organization (2007). Guidelines for the management of sexually transmitted infections. World Health Organization: Geneva, Switzerland.

World Health Organization (2006a). Report of first pan African consultation on alcohol policy and its significance for the region. *African Journal of Drug & Alcohol Studies*, 5(2), 186-191.

World Health Organization (2006b). Reproductive Choices and Family Planning for People Living with HIV. World Health Organization: Geneva, Switzerland.

World Health Organization (2005). Integrated Management of Adolescent and Adult Illness: Acute care guidelines. World Health Organization: Geneva, Switzerland.

World Health Organization (2004). Facilitator's Guide for the WHO Basic ART Aid Training Course. World Health Organization: Geneva, Switzerland.

World Health Organization (2003). Gender Dimensions of HIV Status Disclosure to Sexual Partners: Rates, Barriers, and Outcomes. World Health Organization: Geneva, Switzerland.

World Health Organization (2001). The Alcohol Use Disorders Identification Test (AUDIT): Guidelines for Use in Primary Care. World Health Organization: Geneva, Switzerland.

**APPENDICES**

Appendix A: Consent Form: Pilot Study

Appendix B: Pilot Study Questionnaire

Appendix C: Project Eligibility Screening Form

Appendix D: Consent Form: Patient

Appendix E: Consent Form: Health Care Provider and Lay Counselor

Appendix F: Consent Form: Patient for Observation of Clinic Visit

Appendix G: Contact Information Form

Appendix H: Patient Questionnaire

Appendix I: Clinical Care Survey: Health care provider

Appendix J: Integration of Prevention into Care and Treatment: Health care provider

Appendix K: Integration of Prevention into Care and Treatment: Lay counselor

Appendix L: Lay Counselor Record

Appendix M: Health Care Provider Observation Form

Appendix N: Lay Counselor Observation Form

Appendix O: Clinic Services Form

Appendix P: Patient Medical Chart Review Form

Appendix Q: Incident Report Form (1254)

**Appendix A**

**Pilot Study Patient Consent Form**

###### HIV Prevention for People Living with HIV/AIDS:

###### Evaluation of an Intervention Toolkit for HIV Care and Treatment Settings

**Introduction and Purpose:**

You have been asked to be in a research study. This study is being done in Kenya, Namibia, and Tanzania by the [Country] Ministry of Health [In Kenya it is being conducted by ICAP – Kenya] and the Centers for Disease Control and Prevention (CDC) in the United States. We want to learn more about how health care providers in care and treatment clinics can help their patients stay healthy and protect others from HIV infection. We have some questions we will be asking people. We want to see if people understand the questions we are asking. The purpose is to help us find any problems in the study questions and to fix them. If you tell us during the study about any health or emotional problems you have, we may refer you to see a health care provider or counselor. There will be a total of 60 people in this pilot study.

**Procedures:**

If you choose to be in the study, we will ask you some questions. An interviewer will ask you these questions. Some of the questions will be about your background, your health, what you know about HIV, and what services you get at this clinic. Some other questions will ask about your sexual behavior. After each section we will ask you if there were questions that you did not understand. It will take about one hour to finish the interview.

**Risk/Discomforts:**

Some questions will be personal. For example, we will ask you about your sexual behavior.

**Benefits:**

There is no direct benefit to you for being in this study. What we learn from this study will help us develop programs that will help keep HIV positive people healthy and keep other people from getting HIV.

**Confidentiality:**

Your answers will be kept private. Only study staff (interviewers and the project coordinator) will be able to see any information you give us. We will not write your name on any of your answers to our questions. We will give you a number to protect your privacy. We will keep the records in locked files and only study staff will be allowed to look at them. Your name or anything else that might identify you will not appear in any reports.

**Cost to Participant**

There is no cost to you if you participate in this project. If you are referred for any treatment by study staff, you will have to pay any cost that you would normally pay for the service.

**Compensation:**

We will give you a drink at the end of the interview.

**Right to Refuse or Withdraw:**

It is your choice to be in this study. You can skip any question you do not want to answer. You can drop out of the study at any time. If you decide not be in this study or if you drop out, it will not affect the services you receive here at this clinic or any other clinic.

**Persons to Contact:**

If you have any questions related to: 1) your rights; 2) a research-related injury; and 3) the research study itself please contact

Kenya: either Ms Diana Ngombo, ICAP-Kenya Office, Nairobi; Telephone number: 0733 698029; or contact the Secretary, KEMRI/National Ethics Review Committee, PO BOX 54840-00200, Nairobi; Telephone number: 020-272 2541; 0722 205901, 0733 400003 between 9:00 AM and 5:00 PM, Monday - Friday.

Namibia: Nick DeLuca, CDC-Namibia. Telephone number: 61-385-218

Tanzania: Amy Cunningham, Country Director, ICAP-Tanzania. Telephone number: 78 6199293 or 22 2126729.

**Your Consent:**

I have read this consent form or it has been read to me. I have talked about what it says with the project staff. I had a chance to ask questions and my questions were answered. I was given a copy of this consent form. I agree to be in the study.

Signature

Print Name

Date

Interviewer Signature and ID

**Appendix B**

**Pilot Study Questionnaire**

Participant ID #: __________

Interviewer: Ask the following questions after each section of the questionnaire and write the answers below. Please be sure to write the section and the question number for each response the participant provides.

*Interviewer complete this question:*

What language was the interview conducted in? ___________________

1. What is your age?

2. What is your gender?

­_____ Male

­_____ Female

**3. Were there any questions in this section that you didn’t know how to answer?**

**4. Were there any questions in this section that were hard to answer?**

**5. Were there any questions in this section that didn’t make sense?**

**6. Were there any questions in this section that didn’t allow you to give the answer that you wanted to give?**

**7. Was there anything you didn’t like about this group of questions?**

**8. Were there any words in the questions in this section that you didn’t understand?**

**Appendix C**

**Project Eligibility Screening Form**

| **Complete this box AFTER Screening** |  |  |
| --- | --- | --- |
| Eligible to participate:  Willing to Participate: | **YES**  **YES** | **NO**  **NO** |

Clinic ID #: ________________

Interviewer ID #: ________________

Date of screening:

Day:

Month:

Year: 20

**Screening Script**

Hello, my name is _______________. We are asking people to participate in a research project that will help us learn more about how health care providers can help their HIV-positive patients protect their own health and prevent further HIV infection. This project is being sponsored by the [COUNTRY] Ministry of Health and the U.S. Centers for Disease Control and Prevention – or CDC.

May I tell you more about the study? My questions will only take a few minutes.

*(If NO)* That’s fine. We would like to get some information about why people are not interested in participating. Would you mind telling me why you do not want to participate?

Reason for not participating:

____________________________________________________________________

_____________________________________________________________________

_____________________________________________________________________

_____________________________________________________________________

Can you tell me your age?

Thank you.

*Interviewer: Tick person’s gender: Male Female*

*(If YES)* Great. To see if you are able to be in this research study, I will need to ask you a few questions about your health and your sexual behavior. I will keep all of your answers private, meaning I will not share them with other patients, your health care provider, or your family. Also, your involvement is voluntary, and you can refuse to answer a question or stop at any time. If you can participate but choose not to, there will be no negative effect or any other changes to your medical care and services at this clinic. If you are eligible and want to be in the study, I will ask you to come back to the clinic 3 times over the next year for 1-2 hours to answer more questions. You may be able to answer these questions when you come for your regular clinic visits, if the timing of your visits matches the time for you to answer the questions. If you come on a day that is different from your regular clinic visits, you will get *[equivalent to about US$4]* to cover your transportation expenses. You will not get any money today for talking with me about this study.

May I ask you the questions to see if this study is right for you? The questions will only take a few minutes.

*(If YES),* *CONTINUE TO SCREENING QUESTIONS)*

*(If NO),* *STOP AND READ THE FOLLOWING):*

That’s fine. Do you mind letting me know why you do not want to participate?

*[Write reason here:]* ______________________________________________________

Thank you.

**Screening Questions**

*Interviewer: Circle the answer that best applies.*

“Not eligible”: When an answer marked “Not Eligible” is circled, the individual does not meet study criteria for participation. At that point, the screening questions should be stopped and you should read the statement under “Not Eligible Closing” at the end of the questions.

1. Circle gender

|  | Male | 1 |
| --- | --- | --- |
|  | Female | 2 |

2. How old are you?

|  | 18 or older | 1 |
| --- | --- | --- |
| **Not Eligible** | 17 or younger | 2 |
| **Not Eligible** | Don’t know/refused | 3 |

3. How many times have you seen a health care provider (doctor, nurse, clinical officer) at this HIV clinic?

|  | Patient has had 2 or more visits | 1 |
| --- | --- | --- |
| **Not Eligible** | This is patient’s first or second visit to the clinic | 2 |
| **Not Eligible** | Don’t know/refused | 3 |

4. Do you plan to move away from or stop getting care or treatment from this HIV clinic location over the next year?

|  | No | 1 |
| --- | --- | --- |
| **Not Eligible** | Yes | 2 |
| **Not Eligible** | Don’t know / refused | 3 |

Now I am going to ask you just a few personal questions. It is very important that you answer these questions honestly. Remember, I will keep all of your answers private, meaning I will not share them with your doctor or anyone else.

1. Thinking about the past 3 months, have you had sexual intercourse?

|  | Yes | 1 |
| --- | --- | --- |
| **Not Eligible** | No | 2 |
| **Not Eligible** | Don’t know/refused | 3 |

6. Is your spouse or partner participating in a study in this clinic?

| **Not Eligible** | Yes | 1 |
| --- | --- | --- |
|  | No | 2 |
|  | Don’t know | 3 |

7. *For females:* Are you currently pregnant?

*For males:* Is your spouse or any of your partners currently pregnant?

|  | No | 1 |
| --- | --- | --- |
| **Not Eligible** | Yes | 2 |
| **Not Eligible** | Don’t know/refused | 3 |

If none of the answers were in the shaded “Not Eligible” boxes, then the individual is eligible and can participate in the study. Read the next question and “Eligible Closing” below.

8. Are you currently taking antiretroviral medications (ARVs or ART) to treat your HIV?

|  | Yes | 1 |
| --- | --- | --- |
|  | No | 2 |

**Eligible Closing**:

I would like to invite you to participate in this study about how health care providers and counselors can help their patients living with HIV protect their own heath and protect their partners and loved ones. If you choose to participate, you will be asked to answer more questions about HIV and your sexual behavior. You will meet individually with a study interviewer, who will ask you questions. As I said, if you participate in the study, all of your answers will be kept private and will not be shared with your health care provider, family members, or anyone else. We will never list your name with the answers to the questions you tell us. If you come for an interview on a day that is different from your regular clinic visits, you will get *[equivalent to about US$4]* to cover your transportation expenses.

**Are you interested in participating? (circle) YES NO**

*(If NO)* May I ask why you are not interested in participating?

_____________________________________________________________________

_____________________________________________________________________

_____________________________________________________________________

_____________________________________________________________________

*(If YES)* Great. Are you able to answer these questions today before your clinic visit?

Also, I would like to get your name and contact information so that we can schedule your appointments.

*Interviewer: Complete Consent Form and Contact Information Form*

**Not Eligible Closing:**

I’m sorry, but you are not eligible to participate in this study. There are many possible reasons why people are not able to be in the study. The reasons were decided earlier by the people running the study. We appreciate your interest in this study and your willingness to help us in our research about HIV prevention.

We believe that it is important for everyone to protect themselves and their partners from HIV and other sexually transmitted infections. If you have any questions or would like additional information about HIV you should talk with your provider. Thank you very much for taking the time to answer our questions. Take care.

(Note to Interviewer: In order to be considered eligible for the project, patients must meet all of the following:

(1) at least 18 years of age

(2) sexually active in the prior 3 months

(3) at least 2 prior appointments kept at the HIV care and treatment clinic where enrolled

(4) plans to remain in area and receiving services from clinic site where recruited for next 12-months

(5) able and willing to provide informed consent

(6) not currently pregnant (female patients)/female partner not pregnant (male patients)

**Appendix D**

**Patient Consent Form**

**Patient Consent Form**

###### HIV Prevention for People Living with HIV/AIDS:

###### Evaluation of an Intervention Toolkit for HIV Care and Treatment Settings

**Introduction and Purpose:**

You have been asked to be in a research study. This study is being done in Kenya, Namibia, and Tanzania by the [Country] Ministry of Health [In Kenya it is being conducted by ICAP – Kenya] and the Centers for Disease Control and Prevention (CDC) in the United States. We want to learn more about how health care providers and lay counselors in HIV care and treatment clinics can help their patients stay healthy and protect others from HIV infection. Some clinics in this study will continue to provide the same care to patients that they have been giving. In other study clinics, health care providers and counselors will provide additional prevention information to patients. At the end of the study, the different clinics will be compared and the importance of providing more prevention information to patients will be better understood. Overall, about 3,600 clinic patients in the three different African countries will be in this study. [In Kenya 1200 people at 6 health facilities will participate in the study.] Also, about 180 health care providers and 35 lay counselors will participate.

**Procedures:**

If you choose to be in the study, an interviewer will ask you a series of questions. Some questions will be about your background, your health, and what you know about HIV. We will ask about what services you get at this clinic, how you are feeling, and how you and your partner manage HIV, and other issues in your relationships. Other questions will be sensitive. We will ask about sex and your partners. We will ask if you have been sexually or physically abused. If you tell us during the study about any health or emotional problems you have, we may ask you to see a health care provider or counselor.

We will also record some information from your clinic and laboratory records. What we will record will be about your health, HIV status, and things you have been treated for. This will help us understand more about the care you get at this clinic.

After this interview, there will be two more interviews during the next year. One time will be about 6 months from now. Another time will be about 1 year from now. We will give you times when you can come in for the interview. If you want, you can answer questions on the same day that you come to see your health care provider. The questions asked will be about the same as the first interview. We will ask you for some information so that we can contact you to remind you about your interview as it gets closer.

It will take about one hour to finish the interview each time.

**Risk/Discomforts:**

Some questions will be personal, like about your sexual behavior. Also, if your health care provider talks with you about your sexual behavior, it is possible that you might feel uncomfortable about what he/she says. If that happens, we can refer you to someone who can talk with you about your concerns, if you like.

If you or your partner get pregnant during the time you are in the study, you can still be in the study. There are no risks to you, your partner, or your child from being in this study.

**Benefits:**

There is no direct benefit to you for being in this study. What we learn from this study will help us develop programs that will help keep HIV-positive people healthy and other people from getting HIV.

**Confidentiality:**

If you decide to answer questions, what you talk about will be kept private. Only study staff (interviewers and the project coordinator) will be able to see any information you give us. We will not share this information with anyone, including your health care provider. All of the answers we get will be put together so that no one will know what you said. That means there will be no way for anyone to link what you tell us today with your name or identifying information. Any information we collect that can help us find you will be kept separate from the questions you answer. We will keep the records in locked files. Only study staff will be allowed to look at them. Your name or anything else that might identify you will not appear in any reports.

**Cost to Participant**

There is no cost to you if you participate in this project. If you are referred for any service by study staff, you will have to pay any cost that you would normally pay for the service. However, most services do not have a cost.

**Compensation:**

Each day that you participate we will give you a drink, such as a soda or water. If you come for an interview on a day that you do not have a clinic visit we will give you money for transportation. This amount will be *[each country will insert amount here which will be approximately US$4]*.

**Right to Refuse or Withdraw:**

It is your choice to be in this study. You can skip any question you do not want to answer. You can drop out of the study at any time. If you decide not to be in this study or if you drop out, it will not affect the services you receive here at this clinic or any other clinic. Also, the study staff may decide to take you out of the study if they decide it is in your best interest, or that it is in the best interest of others.

You will be told about any changes to the study that may effect whether you want to continue participating.

**Persons to Contact:**

If you have any questions related to: 1) your rights; 2) a research-related injury; and 3) the research study itself please contact

Kenya: either Ms Diana Ngombo, ICAP-Kenya Office, Nairobi; Telephone number: 0733 698029; or contact the Secretary, KEMRI/National Ethics Review Committee, PO BOX 54840-00200, Nairobi; Telephone number: 020-272 2541; 0722 205901, 0733 400003 between 9:00 AM and 5:00 PM, Monday - Friday.

Namibia: Nick DeLuca, CDC-Namibia. Telephone number: 61-385-218

Tanzania: Amy Cunningham, Country Director, ICAP-Tanzania. Telephone number: 78 6199293 or 22 2126729.

**Your Consent:**

I have read this consent form or it has been read to me. I have talked about what it says with the project staff. I had a chance to ask questions and my questions were answered. I was given a copy of this consent form. I agree to be in the study.

Signature

Print Name

Date

Interviewer Signature and ID

**Appendix E**

**Health Care Provider and Lay Counselor Consent Form**

**Health Care Provider and Lay Counselor Consent Form**

###### HIV Prevention for People Living with HIV/AIDS:

###### Evaluation of an Intervention Toolkit for HIV Care and Treatment Settings

**Introduction and Purpose:**

You have been asked to be in a research study. This study is being done in Kenya, Namibia, and Tanzania by the [Country] Ministry of Health [In Kenya it is being conducted by ICAP – Kenya] and the Centers for Disease Control and Prevention (CDC) in the United States. We want to learn more about how health care providers and lay counselors in HIV care and treatment clinics can help their patients stay healthy and protect others from HIV infection. Overall, about 3,600 clinic patients in the three different African countries will be in this study. [In Kenya 1200 people at 6 health facilities will participate in the study.] Also, about 180 health care providers and 35 lay counselors will participate.

**Procedures:**

If you choose to be in the study, an interviewer will ask you to answer some questions. These questions will be about your routine practices for HIV care and treatment in the HIV clinic. You will fill out questionnaires using a paper and pencil or pen.

After the first questionnaire, there will be 2 more during the next year. One time will be about 6 months from now. The last time will be about 1 year from now.

We will schedule a time for you to complete the questions on a day you are at the HIV clinic. We may ask you your opinion about messages you give or services you offer patients.

It will take about 30 minutes to complete the questionnaires. A study staff person will give you the questionnaires and ensure that you understand the directions.

Also, we may observe you during some of your interactions with patients. We will ask you if we can observe and then you will ask the patient if he or she minds if we come in to observe. We will also get verbal consent from each patient before we observe the visit.

**Risk/Discomforts:**

There are no known risks to you for participating in this study. If you feel uncomfortable with any questions, you may skip them. You may stop participation in the study at any point with no negative consequences to you.

**Benefits:**

There is no direct benefit to you for being in this study. What we learn from this study will help us develop programs that will help keep HIV-positive people healthy and other people from getting HIV.

**Confidentiality:**

If you decide to answer questions, your answers will be kept private. Only study staff (interviewers and project coordinator) will be able to see any information you give us during this project. We will not share this information with anyone. This includes other clinic staff or your supervisors. All of the answers we get will be put together so that no one will know what you said. That means there will be no way for anyone to link what you tell us today with your name or identifying information. We will keep the records in locked files. Only study staff will be allowed to look at them. Your name or anything else that might identify you will not appear in any reports.

**Compensation:**

You will receive no reimbursement for your participation in this study.

**Right to Refuse or Withdraw:**

It is your choice to be in this study. You can skip any question you do not want to answer. You can drop out of the study at any time. If you decide not be in this study or if you decide to leave the study, it will not affect your job at this clinic or any other clinic. Also, the study staff may decide to take you out of the study if they decide it is in your best interest, or that it is in the best interest of others.

You will be told about any changes to the study that may effect whether you want to continue participating.

**Persons to Contact:**

If you have any questions related to: 1) your rights; 2) a research-related injury; and 3) the research study itself please contact

Kenya: either Ms Diana Ngombo, ICAP-Kenya Office, Nairobi; Telephone number: 0733 698029; or contact the Secretary, KEMRI/National Ethics Review Committee, PO BOX 54840-00200, Nairobi; Telephone number: 020-272 2541; 0722 205901, 0733 400003 between 9:00 AM and 5:00 PM, Monday - Friday.

Namibia: Nick DeLuca, CDC-Namibia. Telephone number: 61-385-218

Tanzania: Amy Cunningham, Country Director, ICAP-Tanzania. Telephone number: 78 6199293 or 22 2126729.

**Your Consent:**

I have read this consent form. I have talked about what it says with the project staff. I had a chance to ask questions and my questions were answered. I was given a copy of this consent form. I agree to be in the study.

Signature

Print Name

Date

Interviewer Signature and ID

**Appendix F**

**Patient Consent Form for Observation of Clinic Visit**

**Patient Consent Form for Observation of Clinic Visit**

###### HIV Prevention for People Living with HIV/AIDS:

###### Evaluation of an Intervention Toolkit for HIV Care and Treatment Settings

**Introduction and Purpose:**

You have been asked to be a part of a research study. This study is being done by the [Country] Ministry of Health and the Centers for Disease Control and Prevention (CDC) in the United States. We are doing this project in HIV care and treatment clinics. We want to learn more about how health care providers and lay counselors can help their patients stay healthy and protect others from further HIV infection. We are watching providers and lay counselors during their visits with patients. We want to know what topics they talk about. We would like to watch your visit with the health care provider or lay counselor.

**Procedures:**

If you choose to let us watch your visit today, you will tell us that it is OK for us to watch your visit. We will not ask you any questions or record any information about you. We will record what your health care provider or lay counselor talks to you about. We will also record what information they give you. Whether you agree to let us watch your visit or not will not affect the services you get at this clinic.

**Risk/Discomforts:**

There are no known risks to you for participating in this study. While being observed, you may feel uncomfortable about some personal questions or issues that your health care provider or counselor talks about with you.

**Benefits:**

There is no direct benefit to you for being in this study. What we learn from this study will help us develop programs that will help keep HIV-positive people healthy and other people from getting HIV.

**Confidentiality:**

If you decide to let us watch your visit, what is talked about between you and your health care provider or lay counselor will be kept private. We will not write down anything you say or any information about you. We will only be watching your health care provider or counselor. Only study staff (study interviewers and project coordinator) will be able to see any information we write down. We will not share this information with anyone. There will be no way for anyone to link what happens in the visit with your name or identifying information. We will keep the records in locked files. Only study staff will be allowed to look at them. We will not record your name on anything. Your name or anything else that might identify you will not appear in any reports.

**Compensation:**

You will receive no reimbursement for agreeing to let us watch your visit.

**Right to Refuse or Withdraw:**

It is your choice to be in this study. You can refuse to allow us to observe your visit. You can ask us to leave if you become uncomfortable during the visit.

**Persons to Contact:**

If you have any questions related to: 1) your rights; 2) a research-related injury; and 3) the research study itself please contact

Kenya: either Ms Diana Ngombo, ICAP-Kenya Office, Nairobi; Telephone number: 0733 698029; or contact the Secretary, KEMRI/National Ethics Review Committee, PO BOX 54840-00200, Nairobi; Telephone number: 020-272 2541; 0722 205901, 0733 400003 between 9:00 AM and 5:00 PM, Monday - Friday.

Namibia: Nick DeLuca, CDC-Namibia. Telephone number: 61-385-218

Tanzania: Amy Cunningham, Country Director, ICAP-Tanzania. Telephone number: 78 6199293 or 22 2126729.

Would you like to be in this study?

*If yes then read:*

Please say, “I agree to be in the study.”

*If no then read:*

Thank you for your time.

**Appendix G**

**Contact Information Form**

Interviewer read: Because your continued participation is critical to the success of the study, we want to make sure we will be able to get back in contact with you in time for your next interview. I would like to ask you some questions about the best way to reach you. Please be assured that we will keep information about you confidential, meaning we will not share it with anyone.

Interviewer name and number: _____________________________________________

Is it OK for me to get this contact information from you?

_____ Yes

_____ No 🡺 *[If no, ask next question then do not complete the remainder of*

*this form.]*

Can we contact you again about your next interview?

_____ Yes

_____ No 🡺 *[If No]*: As part of the study, we need for you to come back for

another interview in 6 months. What is the best way to schedule

that interview with you?

*____________________________________________________*

*____________________________________________________*

1. Please give me your full name and spell it for me:

_____________________________________

2. Where are you currently staying?

*[Probe for address or description and location of place.]*

Address:

Description and location:

*[Ask for description of location such as ‘near school.’ If necessary, ask the patient to draw a map of the location.]*

2a. Is this a house, flat, or other type of dwelling?

_____ House

_____ Flat

_____ Other: Specify ___________________________

2b. Whose place is this? __________________________________

*(ENTER ‘SELF’ IF PLACE BELONGS TO PARTICIPANT AND SKIP TO #2e. OTHERWISE, RECORD FULL NAME OF PERSON WHO OWNS THE DWELLING)*

2c. How is this person related to you? ______________________________

2d. Does this person know your HIV status?

_____ Yes

_____ No

2e. Is there a phone number there where we can call you or where you can take phone calls?

_____ Yes

_____ No 🡺 *Skip to 3*

2f. What is the phone number? _____________________________

Is this a cell phone, home phone, work phone, or other phone?

_____ Cell phone

_____ Home phone

_____ Work phone

_____ Other phone: Specify ___________________________

When is the best time for us to call this number? _________________

3. Where else do you sometimes stay? *(IF NONE, SKIP TO #4.)*

*(PROBE FOR ADDRESS OR DESCRIPTION AND LOCATION OF PLACE.)*

Address:

*[Probe for address or description and location of place.]*

Description and location:

*[Ask for description of location such as ‘near school.’ If necessary, ask the patient to draw a map of the location.]*

3a. Is this a house, flat, or other type of dwelling?

_____ House

_____ Flat

_____ Other: Specify ___________________________

3b. Whose place is this? __________________________________

*(ENTER ‘SELF’ IF PLACE BELONGS TO PARTICIPANT AND SKIP TO #3e. OTHERWISE, RECORD FULL NAME OF PERSON)*

3c. How is this person related to you? ______________________________

3d. Does this person know your HIV status?

_____ Yes

_____ No

3e. Is there a phone number there where we can call you or where you can take

phone calls?

_____ Yes

_____ No 🡺 *Skip to 4*

3f. What is the phone number? _____________________________

Is this a cell phone, home phone, work phone, or other phone?

_____ Cell phone

_____ Home phone

_____ Work phone

_____ Other phone: Specify ___________________________

3g. When is the best time for us to call this number? _________________

4. Do you think you will be moving or staying at a different place in the next 6 months or so?

_____ Yes

_____ No 🡺 *Skip to 5*

4a. Where will that be?

Address:

Description and location:

*[Ask for description of location such as ‘near school.’ If necessary, ask the patient to draw a map of the location.]*

4b. Whose place is this? __________________________________

*(ENTER ‘SELF’ IF PLACE BELONGS TO PARTICIPANT AND SKIP TO #5. OTHERWISE, RECORD FULL NAME OF PERSON)*

4c. How is this person related to you? ______________________________

4d. Does this person know your HIV status?

_____ Yes

_____ No

5. I’m going to ask you for phone numbers where we might be able to reach you. Please provide the numbers, whether we can call you there, when is the best time to call you there, and whether we can leave the following message: “This is [*interviewer name*] from the health clinic calling for [*patient name*]. Please ask [*patient name*] to call me back at [*number*].”

| **Phone Type** | **Phone Number** | **Can we call you at this number?** | **When is the best time to call this number?** | **Can we leave the message for you at this number?** |
| --- | --- | --- | --- | --- |
| Cell Phone |  | YES NO |  | YES NO |
| Home Phone |  | YES NO |  | YES NO |
| Work Phone |  | YES NO |  | YES NO |
| Other  Specify: |  | YES NO |  | YES NO |

6. Do you have a job where we can contact you?

_____ Yes

_____ No 🡺 *Skip to 7*

6a. Who are you working for now?

*(ENTER NAME OF EMPLOYER AND LOCATION)*

6b. Does this person know your HIV status?

_____ Yes

_____ No

7. Please give me the names and addresses of two relatives or friends who will know how to get in touch with you. This way we will be able to contact you even if we have somehow lost direct touch with you. We would only attempt to contact these people if we have not be able to reach you directly.

*(RECORD AS MUCH INFORMATION AS POSSIBLE, INCLUDING FULL NAME, ADDRESS OR LOCATION, PHONE NUMBERS AND RELATION TO RESPONDENT FOR 2 CONTACT PERSONS.)*

Contact person #1

Name:

Relationship:

Address/Location:

Phone numbers:

Can we leave a message with them saying, “This is [*interviewer name*] from the health clinic calling for [*patient name*]. Please ask [*patient name*] to call me back at [*number*].”

_____ Yes

_____ No

Contact person #2

Name:

Relationship:

Address/Location:

Phone numbers:

Can we leave a message with them saying, “This is [*interviewer name*] from the health clinic calling for [*patient name*]. Please ask [*patient name*] to call me back at [*number*].”

_____ Yes

_____ No

8. What is the best way to contact you?

By phone? If yes, which phone number? _________________________

In person? If yes, which location? ______________________________

Is there another way? If yes, how? ______________________________

9. What is the best time to contact you? ______________________________

**Appendix H**

**Patient Questionnaire**

HIV Prevention for People Living with HIV/AIDS

Baseline Questionnaire

***Interviewer reads:* “**Thank you for agreeing to meet with me today and participate in this project. Taking part in the survey is up to you. All of the responses that you give will be kept private and will not be shown to others. I will ask you questions that may be uncomfortable to answer. You are free to not answer any questions that you feel are too personal. However, if you take part, you will help us understand more about how to help keep people with HIV healthy and prevent the spread of HIV. If I ask questions you don’t want to answer, just let me know and I will go on to the next question. You can stop the interview at any time. Our meeting will last about 1 hour. Do you have any questions?”

Patient ID #: ______________________

Interviewer ID #: ______________________

Interview Start Time: _________________

Interview End Time: _________________

**Date of interview:**

[ dd / mm / yyyy ]

**Assessment Number:**

_____ 1. Baseline assessment

_____ 2. 6-month assessment

_____ 3. 12-month assessment

**Interview Language:**

_____ 1. English

_____ 2. Kiswahili

_____ 3. Kikuyu

_____ 4. Oshiwambo

_____ 5. Damara-nama

_____ 6. Otjihero

_____ 7. Afrikaans

**Country:** __________________

**Site Name:** ________________

**Sociodemographics**

A1. What is participant’s gender?

_____ 1. Male

_____ 2. Female

A2. In what month and year were you born?

|  |  |
| --- | --- |

[ mm / yyyy ]

*_____ -1. Don’t know*

*_____ -2. Refused*

A3. How old were you on your last birthday? *[compare with # A2 and correct if inconsistent]*

__________ Age in completed years

*_____ -1. Don’t know*

*_____ -2. Refused*

A4. Have you ever attended school*?*

_____ 0. No **🡺** *Skip to A7*

_____ 1. Yes

*_____ -1. Don’t know* 🡺 *Skip to A7*

*_____ -2. Refused* 🡺  *Skip to A7*

A5. What is the highest level of school you attended?

_____ 1. Primary *[typically through 6^th^ year of school*]

_____ 2. Secondary *[typically through 12^th^ year of school or high school]*

_____ 3. Higher *[typically anything beyond secondary school]*

***_____*** *-1. Don’t know*

*_____ -2. Refused*

A6. What is the highest (standard/form/year) you *completed* at that level?

____________________________ Standard/form/year

*_____ -1. Don’t know*

*_____ -2. Refused*

A7. What is your religion? *[Do not read answer choices.]*

_____ 1. Roman Catholic

_____ 2. Protestant

_____ 3. Evangelical / Pentecostal

_____ 4. Islamic / Muslim

_____ 5. Zionist

_____ 6. Apostolic Sect

_____ 7. Other Christian

_____ 8. Other Religion _______________

_____ 9. No Religion

*_____ -1. Don’t know*

*_____ -2. Refused*

A8. What is your marital status now?

_____ 1. Married

_____ 2. Living together as married

_____ 3. Never Married 🡺 *Skip to A12*

_____ 4. Widowed 🡺 *Skip to A12*

_____ 5. Divorced 🡺 *Skip to A12*

_____ 6. Separated 🡺 *Skip to A12*

*_____ -1. Don’t know*

*_____ -2. Refused* 🡺 *Skip to A12*

**FOR MALES ONLY**

A9. Altogether, how many wives do you have or other partners do you live with as if married?

__________ Total number of wives and live-in partners 🡺 *Skip to A11*

*_____ -1. Don’t know*

*_____ -2. Refused*

**FOR FEMALES ONLY**

A10. Including yourself, in total, how many wives or other partners does your husband/partner live with now as if married?

__________ Number of wives and live-in partners

*_____ -1. Don’t know*

*_____ -2. Refused*

**FOR BOTH MALES AND FEMALES**

A11. How long have you been living with your husband/wife/partner?

_____1. Number of days

**_____**2. Number of weeks

_____3. Number of months

_____4. Number of years

*_____ -1. Don’t know month*

*_____ -1. Don’t know year*

*_____ -2. Refused*

A12. Do you have any children?

_____ 0. No 🡺 *Skip to A16*

_____ 1. Yes

*_____ -1. Don’t know*

*_____ -2. Refused*

A13. How many children do you have that are living?

__________ Number of children

*_____ -1. Don’t know*

*_____ -2. Refused*

A14. How many of your children are living with you now?

__________ Number of children 🡺 *If 0, skip to A16*

*_____ -1. Don’t know*

*_____ -2. Refused*

A15. What is the age of your youngest child living with you now?

__________ Age of youngest child

A16. Have you done any paid work in the last 6 months?

_____ 0. No 🡺 *Skip to A18*

_____ 1. Yes

*_____ -1. Don’t know*

*_____ -2. Refused*

A17. Have you done any paid work in the last 7 days?

_____ 0. No

_____ 1. Yes 🡺 *Skip to A19*

*_____ -1. Don’t know*

*_____ -2. Refused*

A18. What is the main reason you are not working for pay now? *[Tick all that apply. Prompt with answer choices if needed.]*

_____ 1. Work occasionally

_____ 2. Work certain times of year

_____ 3. Study/in school

_____ 4. Am married

_____ 5. Spouse against me working

_____ 6. Taking care of children

_____ 7. Need to help in the house

_____ 8. No need to/don’t like

_____ 9. Health problems

_____ 10. Was fired

_____ 11. Can’t find work

_____ 12. Husband/wife/partner supports me

_____ 13. Other (specify) _______________

*_____ -1. Don’t know*

*_____ -2. Refused*

A19. What is your monthly household income? By household, I mean you and your spouse or partner who lives with you.

__________1. Kenyan Shillings

__________2. Namibian Dollars

__________3. Tanzanian Shillings

__________4. Other

If other, please specify which: _______________________

*_____ -1. Don’t know*

*_____ -2. Refused*

| ***Interviewer reads:*** *“*The next questions are about the household where you live/stay. Exclude corridors, walkways, and storage areas.” |
| --- |

A20. How many separate rooms are in your house, including all living areas, bathrooms, washrooms, and any other rooms? Please do not include outside bathrooms.

__________ Number of rooms

*_____ -1. Don’t know*

*_____ -2. Refused*

A21. How many rooms in this household are used for sleeping?

__________ Number of sleeping rooms

*_____ -1. Don’t know*

*_____ -2. Refused*

A22. How many people usually live in your household, including yourself and other adults or children?

__________ Number of people in household

*_____ -1. Don’t know*

*_____ -2. Refused*

| ***Interviewer reads:*** *“*The next questions are about travel to get to this clinic.” |
| --- |

A23. How far in kilometers is your house from this clinic?

__________ Distance in kilometers [1 km = 0.62 miles]

__________ Distance in miles

*_____ -1. Don’t know*

*_____ -2. Refused*

A24. How long does it usually take you to get from your house to this clinic?

__________ Time in minutes [1 hour = 60 minutes]

__________ Time in hours

*_____ -1. Don’t know*

*_____ -2. Refused*

A25. What is your primary means of transportation to this clinic? *[Tick all that apply.]*

_____ 1. Walking

_____ 2. Bicycle

_____ 3. Matatu or dala dala or bus/taxi

_____ 4. Motorcycle

_____ 5. Privately owned car

_____ 6. Special hire taxi

_____ 7. Boat/ferry

_____ 8. Other (specify) ____________________

*_____ -1. Don’t know*

*_____ -2. Refused*

A26. How much money does it cost for you to travel from your house to this clinic one-way?

_____1. Kenyan Shillings

_____2. Namibian Dollars

_____3. Tanzanian Shillings

_____4. Other

If other, please specify which: ______________________

*_____ -1. Don’t know*

*_____ -2. Refused*

A27. Have there been times you were scheduled to come to the clinic or needed to come to the clinic but you were not able to come?

_____ 0. No 🡺 *Skip to B1 in next section*

_____ 1. Yes

*_____ -1. Don’t know*

*_____ -2. Refused*

A28. Why were you not able to make it to the clinic*? [Read all options. Tick all that apply.]*

_____ 1. Not able to get away from work

_____ 2. Not able to pay for transportation

_____ 3. No transportation to clinic

_____ 4. Don’t want people to know I am coming to the clinic

_____ 5. Don’t have enough time to come to clinic

_____ 6. Other: Specify _______________________________

_____ 7. Other: Specify _______________________________

*_____ -1. Don’t know*

*_____ -2. Refused*

**HIV Testing and Care**

| ***Interviewer reads:*** *“*Now I am going to ask you some questions about HIV testing and your HIV care.” |
| --- |

B1. When did you find out that you have HIV?

|  |  |
| --- | --- |

[ mm / yyyy ]

*_____ -1. Don’t know*

*_____ -2. Refused*

B2. Where were you tested for HIV?

**_____** 1. VCT (Voluntary Counseling and Testing) centre

**_____** 2. PMTCT clinic

**_____** 3. Community testing

**_____** 4. Inpatient

**_____** 5. Outpatient

**_____** 6. Other (specify) _____________________________

*_____ -1. Don’t know*

*_____ -2. Refused*

B3. Why were you tested for HIV? *[Read all options. Tick all that apply.]*

_____ 1. Doctor encouraged me to get tested

_____ 2. Family or friend encouraged me to get tested

_____ 3. My partner was tested

_____ 4. I heard on the radio/TV that I should get tested

_____ 5. I was pregnant and the clinic tested me

_____ 6. I felt sick

_____ 7. Other (specify) ________________

*_____ -1. Don’t know*

*_____ -2. Refused*

B4. When was the last time your CD4 count was tested?

|  |  |
| --- | --- |

[ mm / yyyy ]

*_____ -1. Don’t know*

*_____ -2. Refused*

B5. What was your CD4 count last time you were tested?

_____ [CD4 count]

*_____ -1. Don’t know*

*_____ -2. Refused*

B6. How long after you learned you were HIV positive did you go to an HIV clinic for care? *[If started getting care immediately, write ‘0’ in for the month.]*

_________ Months

_________ Years

*_____ -1. Don’t know*

*_____ -2. Refused*

B7. How many total times in the past 6 months have you been to this clinic?

__________ Number of times

*_____ -1. Don’t know*

*_____ -2. Refused*

B7a. How many of these times did you just pick up HIV medications and not see a health care provider?

__________ Number of times

***_____*** *-1. Don’t know*

*_____ -2. Refused*

B8. Were you getting HIV care, treatment, or HIV medications at other clinics before coming to this clinic?

_____ 0. No

_____ 1. Yes

***_____*** *-1. Don’t know*

*_____ -2. Refused*

**Physical and Mental Functioning**

| ***Interviewer Reads:*** *“*Now I am going to ask you some questions related to your health.” |
| --- |

C1. Overall, how would you rate your health during the past 4 weeks?

_____ 1. Excellent

_____ 2. Very Good

_____ 3. Good

_____ 4. Fair

_____ 5. Poor

_____ 6. Very Poor

*_____ -1. Don’t know*

*_____ -2. Refused*

C2. During the past 4 weeks, how much did physical health problems limit your usual physical activities (such as walking or climbing stairs)?

_____ 1. Not at all

_____ 2. Very little

_____ 3. Somewhat

_____ 4. Quite a lot

_____ 5. Could not do physical activities

*_____ -1. Don’t know*

*_____ -2. Refused*

C3. During the past 4 weeks, how much difficulty did you have doing your daily work, both at home and away from home because of your physical health?

_____ 1. Not at all

_____ 2. A little bit

_____ 3. Some

_____ 4. Quite a lot

_____ 5. Could not do daily work

*_____ -1. Don’t know*

*_____ -2. Refused*

C4. How much bodily pain have you had during the past 4 weeks?

_____ 1. None

_____ 2. Very mild

_____ 3. Mild

_____ 4. Moderate

_____ 5. Severe

_____ 6. Very Severe

*_____ -1. Don’t know*

*_____ -2. Refused*

C5. During the past 4 weeks, how much energy did you have?

_____ 1. Very much

_____ 2. Quite a lot

_____ 3. Some

_____ 4. A little

_____ 5. None

*_____ -1. Don’t know*

*_____ -2. Refused*

C6. During the past 4 weeks, how much did your physical health or emotional problems limit your usual social activities with family or friends?

_____ 1. Not at all

_____ 2. Very little

_____ 3. Somewhat

_____ 4. Quite a lot

_____ 5. Could not do social activities

*_____ -1. Don’t know*

*_____ -2. Refused*

C7. During the past 4 weeks, how much have you been bothered by emotional problems (such as feeling anxious, depressed, or irritable)?

_____ 1. Not at all

_____ 2. Slightly

_____ 3. Moderately

_____ 4. Quite a lot

_____ 5. Extremely

*_____ -1. Don’t know*

*_____ -2. Refused*

C8. During the past 4 weeks, how much did personal or emotional problems keep you from doing your usual work, school, or other daily activities?

_____ 1. Not at all

_____ 2. Very little

_____ 3. Somewhat

_____ 4. Quite a lot

_____ 5. Could not do daily activities

*_____ -1. Don’t know*

*_____ -2. Refused*

**Perceived Availability of Support**

| ***Interviewer reads:*** “The following questions have to do with the support you get from people in your life. I will read you a series of questions concerning different types of help people might give you. Please tell me whether someone would be available to provide that kind of help or support if you needed it. Remember that I’m not asking whether or not you need this kind of help at this time, but whether someone could help you if you needed. Please answer ‘yes’ or ‘no’ to each of these questions.” |
| --- |

*[****Interviewer:*** *Circle the correct answer for each question.]*

|  | **No** | **Yes** | *Not Applicable* |
| --- | --- | --- | --- |
| D1. Would someone be available to talk to you if you were upset, nervous or depressed? | 0 | 1 |  |
| D2. Is there someone you could contact if you wanted to talk about an important personal problem you were having? | 0 | 1 |  |
| D3. Is there someone who would help take care of you if you had to stay in bed for several weeks? | 0 | 1 |  |
| D4. Is there someone you could turn to if you needed to borrow money, get help getting to the doctor, or some other small immediate help? | 0 | 1 |  |
| D5. Is there someone you could turn to if you needed to borrow money for a medical emergency? | 0 | 1 |  |
| D6. Would the people in your personal life give you information, suggestions, or guidance if you needed it? | 0 | 1 |  |
| D7. Is there someone you could turn to if you needed advice to help make a decision? | 0 | 1 |  |
| D8. Is there someone who could take care of your children if you got sick? | 0 | 1 | *-3* |

**CES-D (Center for Epidemiologic Studies – Depression) Scale**

| ***Interviewer reads****:* “Now I would like to ask you some questions about how you have been feeling during the past week. I am going to read you a list of ways you may have been feeling. Please tell me how often in the past week you have felt that way using the following scale.” |
| --- |

*[****Interviewer:*** *Circle the correct answer for each question.]*

|  | **Rarely**  **(<1 day)** | **Some days**  **(1-2 days)** | **Occasionally**  **(3-4 days)** | **Most days**  **(5-7 days)** |
| --- | --- | --- | --- | --- |
| E1. I was bothered by things that usually don’t bother me. | 1 | 2 | 3 | 4 |
| E2. I did not feel like eating; my appetite was poor. | 1 | 2 | 3 | 4 |
| E3. I felt that I could not shake off the blues even with help from my family or friends. | 1 | 2 | 3 | 4 |
| E4. I felt I was just as good as other people. | 1 | 2 | 3 | 4 |
| E5. I had trouble keeping my mind on what I was doing. | 1 | 2 | 3 | 4 |
| E6. I felt depressed. | 1 | 2 | 3 | 4 |
| E7. I felt that everything I did was an effort. | 1 | 2 | 3 | 4 |
| E8. I felt hopeful about the future. | 1 | 2 | 3 | 4 |
| E9. I thought my life had been a failure. | 1 | 2 | 3 | 4 |
| E10. I felt fearful. | 1 | 2 | 3 | 4 |
| E11. My sleep was restless. | 1 | 2 | 3 | 4 |
| E12. I was happy. | 1 | 2 | 3 | 4 |
| E13. I talked less than usual. | 1 | 2 | 3 | 4 |
| E14. I felt lonely. | 1 | 2 | 3 | 4 |
| E15. People were unfriendly. | 1 | 2 | 3 | 4 |
| E16. I enjoyed life. | 1 | 2 | 3 | 4 |
| E17. I had crying spells. | 1 | 2 | 3 | 4 |
| E18. I felt sad. | 1 | 2 | 3 | 4 |
| E19. I felt that people dislike me. | 1 | 2 | 3 | 4 |
| E20. I could not get “going.” | 1 | 2 | 3 | 4 |

**Alcohol Use: WHO Alcohol Use Disorders Identification Test (AUDIT)**

| ***Interviewer reads:*** “Now I am going to ask you some questions about your use of alcoholic beverages during the past 6 months. By a drink we mean 15 milliliters (half an ounce) of absolute alcohol. For example, a 350 ml can or glass of beer, a 150 ml glass of wine, or a drink containing 15 ml of liquor. There are no right or wrong answers to this. Please try to be as honest and as accurate as you can be.” |
| --- |

*[****Interviewer:*** *Read questions as written. Explain what is meant by “alcoholic beverages” by using local examples of beer, wine, vodka, etc. Code answers in terms of “standard drinks”.* ***Circle the correct answer for each question****.]*

|  | **0** | **1** | **2** | **3** | **4** |
| --- | --- | --- | --- | --- | --- |
| F1. How often do you have a drink containing alcohol? | Never  [*Skip to F9*] | Monthly or less | 2 to 4 times a month | 2 to 3 times a week | 4 or more times a week |
| F2. How many drinks containing alcohol do you have on a typical day when you are drinking? | 1 or 2 | 3 or 4 | 5 or 6 | 7, 8, or 9 | 10 or more |
| F3. How often do you have six or more drinks on one occasion? | Never | Less than monthly | Monthly | Weekly | Daily or almost daily |
| F4. How often during the last 6 months have you found that you were not able to stop drinking once you had started? | Never | Less than monthly | Monthly | Weekly | Daily or almost daily |
| F5. How often during the last 6 months have you failed to do what was normally expected of you because of drinking? | Never | Less than monthly | Monthly | Weekly | Daily or almost daily |
| F6. How often during the last 6 months have you needed a first drink in the morning to get yourself going after a heavy drinking session? | Never | Less than monthly | Monthly | Weekly | Daily or almost daily |
| F7. How often during the last 6 months have you had a feeling of guilt or remorse after drinking? | Never | Less than monthly | Monthly | Weekly | Daily or almost daily |
| F8. How often during the last 6 months have you been unable to remember what happened the night before because of your drinking? | Never | Less than monthly | Monthly | Weekly | Daily or almost daily |
| F9. Have you or someone else been injured because of your drinking? | No |  | Yes, but not in the last 6 months |  | Yes, during the last 6 months |
| F10. Has a relative or a friend or a doctor or another health care worker been concerned about your drinking or suggested you cut down? | No |  | Yes, but not in the last 6 months |  | Yes, during the last 6 months |

**HIV Antiretroviral Medications and Adherence**

| ***Interviewer reads:*** “Now I have questions about medications you may take for HIV care or treatment. By HIV medications I mean HIV antiretroviral medications, cotrimoxazole, Bactrim, or Septrim. Please do not include multivitamins or medications for TB or other infections.” |
| --- |

G1. Do you take medication for your HIV?

_____ 0. No 🡺 *Skip to G10*

_____ 1. Yes

*_____-1. Don’t know* 🡺 *Skip to G10*

*_____-2. Refused* 🡺 *Skip to G10*

**Only for Participants taking HIV Medications**

G2. When did you first start taking HIV medications?

[ dd / mm / yyyy ]

*_____-1. Don’t know*

*_____-2. Refused*

**G3.**

| ***Interviewer reads:*** “Most people with HIV have many pills to take at different times during the day. Many people find it hard to always remember their pills:   - Some people get busy and forget to carry their pills with them. - Some people find it hard to take their pills according to all the instructions,   such as “with meals” or “on an empty stomach,” “every 8 hours,” “with plenty of fluids.”   - Some people decide to skip pills to avoid side effects or to just not be taking pills that day.   We need to understand how people with HIV are really doing with their pills. Please tell us what you are **actually** doing. Don’t worry about telling us that you don’t take all your pills. We need to know what is really happening, not what you think we “want to hear.”  We would like to know what HIV medications you are taking now. Please tell me   - the names of the HIV medications you have been taking in the last 30 days, - what the medication is for, - how many pills you are supposed to take each time, and - how many times per day you are supposed to take each medication. Each time you take medications during the day is a dose.   Please tell me the name of the HIV medications.” |
| --- |

| **G3. HIV Medication Name** | **What is this medication for?** | **# pills each time (pills each dose)** | **# times per day (doses per day)** |
| --- | --- | --- | --- |
| a. **______________________**  *_____ -1. Don’t know*  *_____ -2. Refused* | **_________________**  *_____ -1. Don’t know*  *_____ -2. Refused* | **___________**  *__-1. Don’t know*  *__ -2. Refused* | **___________**  *__-1. Don’t know*  *__ -2. Refused* |
| b. **______________________**  *_____ -1. Don’t know*  *_____ -2. Refused* | **_________________**  *_____ -1. Don’t know*  *_____ -2. Refused* | **___________**  *__-1. Don’t know*  *__ -2. Refused* | **___________**  *__-1. Don’t know*  *__ -2. Refused* |
| c. **______________________**  *_____ -1. Don’t know*  *_____ -2. Refused* | **_________________**  *_____ -1. Don’t know*  *_____ -2. Refused* | **___________**  *__-1. Don’t know*  *__ -2. Refused* | **___________**  *__-1. Don’t know*  *__ -2. Refused* |
| d. **______________________**  *_____ -1. Don’t know*  *_____ -2. Refused* | **_________________**  *_____ -1. Don’t know*  *_____ -2. Refused* | **___________**  *__-1. Don’t know*  *__ -2. Refused* | **___________**  *__-1. Don’t know*  *__ -2. Refused* |

G4.

| ***Interviewer Reads:*** “The next section of the questionnaire asks about the medications that you may have **missed** taking over the last four days and last 30 days.” |
| --- |

*[****Interviewer:*** *Complete the table below, using one line for each medication the patient is taking. Write in the medications from the previous question. The same medication should be repeated in the same place on both this question and the previous question (for instance in ‘a’ for both questions). For each medication, ask the patient how many DOSES he or she missed in the last 4 days, and in the last 30 days. If patient did not miss any doses, write a zero (0) in the box. If the patient missed ALL their doses, write the number that they missed.*

*Note that the table asks about DOSES, not PILLS.*

*DOSES are the number of times the patient takes their pills during the day. For example, if they take pills in the morning and the evening they have 2 DOSES per day. They may take 1 pill, 2 pills, or more during each DOSE.]*

***[IF PATIENT TOOK ONLY A PORTION OF A DOSE ON ONE OR MORE OF THESE DAYS, PLEASE REPORT THE DOSE(S) AS BEING MISSED.]***

|  | How many doses did you **miss**…  By dose I mean each time you take medications during the day. | |
| --- | --- | --- |
| **Step 1**  Abbreviations / Drug names | **Step 2**  In the last 4 days | **Step 3**  In the last 30 days |
| 1. **__________________________**   ***_____*** *-1. Don’t know*  ***_____*** *-2. Refused* | ____________ doses  ***_____*** *-1. Don’t know*  *_____ -2. Refused* | ____________ doses  ***_____*** *-1. Don’t know*  *_____ -2. Refused* |
| 1. **____________________________**   ***_____*** *-1. Don’t know*  ***_____*** *-2. Refused* | ____________ doses  ***_____*** *-1. Don’t know*  *_____ -2. Refused* | ____________ doses  ***_____*** *-1. Don’t know*  *_____ -2. Refused* |
| 1. **____________________________**   ***_____*** *-1. Don’t know*  ***_____*** *-2. Refused* | ____________ doses  ***_____*** *-1. Don’t know*  *_____ -2. Refused* | ____________ doses  ***_____*** *-1. Don’t know*  *_____ -2. Refused* |
| 1. **____________________________**   ***_____*** *-1. Don’t know*  ***_____*** *-2. Refused* | ____________ doses  ***_____*** *-1. Don’t know*  *_____ -2. Refused* | ____________ doses  ***_____*** *-1. Don’t know*  *_____ -2. Refused* |

G5. Most medications need to be taken on a schedule, such as ‘2 times a day’ or ‘every 12 hours.’ How closely did you follow your specific schedule over the last 4 days?

_____ 0. Never

_____ 1. Some of the time

_____ 2. About half of the time

_____ 3. Most of the time

_____ 4. All of the time

*_____-1. Don’t know*

*______-2. Refused*

G6. *[****Interviewer:*** *Show the participant the drawing and ruler while you are reading the directions.]*

| ***Interviewer Reads:*** “I want to ask about your experience taking your HIV medicines over the past 30 days. Please look at this drawing and ruler. The first cup on the left is empty and represents taking none of your antiretroviral doses in the past 30 days. The last cup on the right is full and represents taking all of your antiretroviral doses in the past 30 days. Using these pictures as a guide, please show me on the line how much of your antiretroviral medicine you took in the past 30 days. For example, if you took half of your doses during the past 30 days, you would select number 5.” |
| --- |

**NONE**

**0**

**ALL**

**10**

**
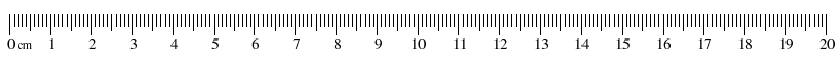
**

*_____-1. Don’t know*

*_____-2. Refused*

G7. When was the last time you missed any of your medications?

_____ 5. Within the past week

_____ 4. 1-2 weeks ago

_____ 3. 2-4 weeks ago

_____ 2. 1-3 months ago

_____ 1. More than 3 months ago

_____ 0. Never skip medications 🡺 *Skip to H1 in next section*

*_____-1. Don’t know*

*_____-2. Refused*

G8. Some people find that they may go without pills for more than 2 days at a time. In the last 6 months, have you ever not taken or missed your medications for more than 2 days at a time?

_____ 0. No 🡺 Go to H1

_____ 1. Yes

*_____-1. Don’t know*

*_____-2. Refused*

G9. Here is a list of possible reasons why you may miss taking your medications for more than 2 days at a time. In the last 6 months, have you missed taking your HIV medications for more than 2 days at a time because you:

|  | **No** | **Yes** | *Don’t know* | *Refused* |
| --- | --- | --- | --- | --- |
| 1. Were away from home? | 0 | 1 | *-1* | *-2* |
| 1. Were busy with other things? | 0 | 1 | *-1* | *-2* |
| 1. Simply forgot? | 0 | 1 | *-1* | *-2* |
| 1. Had too many pills to take? | 0 | 1 | *-1* | *-2* |
| 1. Wanted to avoid side effects? | 0 | 1 | *-1* | *-2* |
| 1. Did not want others to notice you taking medication? | 0 | 1 | *-1* | *-2* |
| 1. Had a change in daily routine? | 0 | 1 | *-1* | *-2* |
| 1. Felt like the drug was toxic/harmful? | 0 | 1 | *-1* | *-2* |
| 1. Fell asleep/slept through dose time? | 0 | 1 | *-1* | *-2* |
| 1. Felt sick or ill? | 0 | 1 | *-1* | *-2* |
| 1. Felt depressed /overwhelmed? | 0 | 1 | *-1* | *-2* |
| 1. Had problems with taking pills at specified times (with meals, on empty stomach, etc.)? | 0 | 1 | *-1* | *-2* |
| 1. Ran out of pills? | 0 | 1 | *-1* | *-2* |
| 1. Felt good? | 0 | 1 | *-1* | *-2* |
| 1. Did not have enough food to take with pills? | 0 | 1 | *-1* | *-2* |
| 1. Could not get transportation to the clinic to pick up more pills? | 0 | 1 | *-1* | *-2* |

*[After completing G9, go to H1 in next section.]*

**Only for Participants NOT taking HIV Medications**

G10.

| ***Interviewer Reads:*** “The following are some reasons other people have told us why they are **not** currently taking any HIV medications. After I read you each reason, please tell me if it describes you.” |
| --- |

|  | **No** | **Yes** | *Don’t know* | *Refused* |
| --- | --- | --- | --- | --- |
| 1. No doctor or other medical provider has ever offered HIV medications to me. | 0 | 1 | *-1* | *-2* |
| 1. A medical provider offered them to me, but I choose not to take HIV medications at this time. | 0 | 1 | *-1* | *-2* |
| 1. I discussed taking HIV medications with my medical provider, but he/she did not think it was a good idea for me now. | 0 | 1 | *-1* | *-2* |
| 1. My CD4 (T-cell) count is too high for me to take HIV medications. | 0 | 1 | *-1* | *-2* |
| 1. I tried HIV medications, but there were too many unpleasant side effects. | 0 | 1 | *-1* | *-2* |
| 1. I tried HIV medications, but they were not effective for me. | 0 | 1 | *-1* | *-2* |
| 1. I tried HIV medications, but they were too difficult to take as prescribed. | 0 | 1 | *-1* | *-2* |
| 1. I cannot afford them. | 0 | 1 | *-1* | *-2* |
| 1. Is there any other reason why you are not currently taking any HIV medications?   ***If yes*:** Reason __________________________ | 0 | 1 | *-1* | *-2* |

**Disclosure**

| ***Interviewer Reads:*** “Now, I am going to ask you some questions about people you have told that you are HIV-positive.” |
| --- |

H1. Have you ever told anyone that you are HIV positive?

_____ 0. No 🡺 *Skip to H5*

_____ 1. Yes

*_____-1. Don’t know*

*_____-2. Refused*

H2. How many people have you told?

__________ Number told

*_____-1. Don’t know*

*_____-2. Refused*

H3. How many household members have you told?

__________ Number told

*_____-1. Don’t know*

*_____-2. Refused*

H4. Have you ever disclosed your HIV status to any of the following people?

|  | **No** | **Yes** | *Don’t know* | *Refused* |
| --- | --- | --- | --- | --- |
| a. Any parent? | 0 | 1 | *-1* | *-2* |
| b. Any biological child? | 0 | 1 | *-1* | *-2* |
| c. Any relative? | 0 | 1 | *-1* | *-2* |
| d. Any friend? | 0 | 1 | *-1* | *-2* |
| e. Any spouse? | 0 | 1 | *-1* | *-2* |
| f. Any sex partner? | 0 | 1 | *-1* | *-2* |

H5. Are you worried about other people finding out your HIV status?

_____ 0. No

_____ 1. Yes

*_____-1. Don’t know*

*_____-2. Refused*

H6. What do you think might happen if people that you have not told found out your HIV status?  *[Do not read responses. Tick all that apply.]*

_____ 1. Might be fired/lose a job/denied a job

_____ 2**.** Family members might treat you differently/not respect your opinion

_____ 3. Might lose friends

_____ 4. Partner might get violent

_____ 5. People might start gossiping about you

_____ 6. Might be kicked out of your home/ become homeless

_____ 7. In-laws might encourage spouse to leave you

_____ 8. Your children might be abused or discriminated against

_____ 9. Your children might become upset/fearful

_____ 10. Might not get good care/hospital/home/school etc.

_____ 11. Might cause the other person’s health to deteriorate or other

person to become anxious

_____ 12. Nothing

_____ 13. Other (Specify):__________________

*_____-1. Don’t know*

*_____-2. Refused*

**HIV/AIDS** **Knowledge**

| ***Interviewer reads:*** “Now I would like to ask you some questions about HIV and AIDS.” |
| --- |

H101. If a man has the virus that causes AIDS, does his sexual partner always have the AIDS virus, almost always, or only sometimes?

_____ 1. Always

_____ 2. Almost always

_____ 3. Only sometimes

*_____-1. Don’t know*

*_____-2. Refused*

H102. If a woman has the virus that causes AIDS, does her sexual partner always have the AIDS virus, almost always, or only sometimes?

_____ 1. Always

_____ 2. Almost always

_____ 3. Only sometimes

*_____-1. Don’t know*

*_____-2. Refused*

H103. What are the main channels of communication from which you receive AIDS information and education? Probe: Any other channels*?* *[Don’t read responses. Record ALL channels mentioned.]*

_____ 1. Radio

_____ 2. Television

_____ 3. Film

_____ 4. Drama

_____ 5. Newspapers/magazines

_____ 6. Brochures

_____ 7. Posters

_____ 8. Billboards

_____ 9. Community notices

_____ 10. Family

_____ 11. Friends

_____ 12. Peers

_____ 13. Health workers

_____ 14. Teachers

_____ 15. Political leaders

_____ 16. Traditional leaders

_____ 17. Religious leaders

_____ 18. Internet

_____ 19. Other people living with HIV/AIDS

_____ 20. Other (specify) _____________________

*_____-1. Don’t know*

*_____-2. Refused*

**Patient Sex Behavior**

| ***Interviewer reads:*  “**Now I would like to ask you some questions about your recent sexual activity. We know these questions are sensitive and want to remind you that your answers are completely private and will not be shared with anyone. No one will know what particular answers you give. It is perfectly normal for persons with HIV to have sex. We are not judging you on your sexual behaviors. We want you to be as honest as you can answering these questions. If we should come to any questions that you don’t want to answer, just let me know and we will go to the next one.  Different people have different definitions of ‘sex’ or ‘sexual intercourse.’ For this study, sex can include several things, such as:   - Vaginal sex, which is when a man puts his penis in a woman’s vagina. - Anal sex, which is when a man puts his penis in another person’s rectum or butt.” |
| --- |

J1. How old were you when you had sexual intercourse for the very first time?

__________Age in years

*_____-1. Don’t know*

*_____-2. Refused*

J2. In total, how many different people have you had sexual intercourse with in your entire life?

__________Total number of lifetime partners

*_____-1. Don’t know*

*_____-2. Refused*

J3. Have you had sexual intercourse in the last 3 months?

_____ 0. No 🡺 *Skip to K1 in next section.*

_____ 1. Yes

*_____-1. Don’t know*

*_____-2. Refused*

J4. In total, how many people have you had sex with in the last 3 months?

__________ Total number of partners

*_____-1. Don’t know*

*_____-2. Refused*

J5. In total, how many people have you had sex with in the last 2 weeks?

__________ Total number of partners

*_____-1. Don’t know*

*_____-2. Refused*

| ***Interviewer reads:*** “I am going to ask you about the people you had sex with over the past 90 days. Please tell me about them starting with your most recent sexual partner.” |
| --- |

*[****Interviewer:*** *Read down each column of the table (for each partner, one at a time),* ***not*** *across each row.]*

|  | **Partner 1** | **Partner 2** | **Partner 3** | **Partner 4** | **Partner 5** |
| --- | --- | --- | --- | --- | --- |
| J6. What is the first name or a nickname of this partner? | ___ *-1. Don’t know*  ___ *-2. Refused* | ___ *-1. Don’t know*  ___ *-2. Refused* | ___ *-1. Don’t know*  ___ *-2. Refused* | ___ *-1. Don’t know*  ___ *-2. Refused* | ___ *-1. Don’t know*  ___ *-2. Refused* |
| For the next question I am going to ask you if a partner is a **spouse**, **main partner**, or **non-main partner**.   - By **spouse** we mean someone who you are married to or living with as if married. - By **main partner** we mean someone who you are NOT married to or living with as if married, but who is a steady partner such as a boyfriend or girlfriend. - By **non-main partner** we mean someone who is NOT your spouse or a main partner, but with whom you have had sex with in the last 3 months. | | | | | |
| J7. Is this person your spouse, main partner, or non-main partner? | ___ 1. Spouse  ___ 2. Main  ___ 3. Non-main  ___ *-1. Don’t know*  ___ *-2. Refused* | ___ 1. Spouse  ___ 2. Main  ___ 3. Non-main  ___ *-1. Don’t know*  ___ *-2. Refused* | ___ 1. Spouse  ___ 2. Main  ___ 3. Non-main  ___ *-1. Don’t know*  ___ *-2. Refused* | ___ 1. Spouse  ___ 2. Main  ___ 3. Non-main  ___ *-1. Don’t know*  ___ *-2. Refused* | ___ 1. Spouse  ___ 2. Main  ___ 3. Non-main  ___ *-1. Don’t know*  ___ *-2. Refused* |
| J8. When was the first time you had sex with this partner? | ________ Days ago  ________ Weeks ago  ________ Months ago  ________ Years ago  ___ *-1. Don’t know*  ___ *-2. Refused* | ________ Days ago  ________ Weeks ago  ________ Months ago  ________ Years ago  ___ *-1. Don’t know*  ___ *-2. Refused* | ________ Days ago  ________ Weeks ago  ________ Months ago  ________ Years ago  ___ *-1. Don’t know*  ___ *-2. Refused* | ________ Days ago  ________ Weeks ago  ________ Months ago  ________ Years ago  ___ *-1. Don’t know*  ___ *-2. Refused* | ________ Days ago  ________ Weeks ago  ________ Months ago  ________ Years ago  ___ *-1. Don’t know*  ___ *-2. Refused* |
| J9. When was the last time you had sex with this partner? | ________ Days ago  ________ Weeks ago  ________ Months ago  ___ *-1. Don’t know*  ___ *-2. Refused* | ________ Days ago  ________ Weeks ago  ________ Months ago  ___ *-1. Don’t know*  ___ *-2. Refused* | ________ Days ago  ________ Weeks ago  ________ Months ago  ___ *-1. Don’t know*  ___ *-2. Refused* | ________ Days ago  ________ Weeks ago  ________ Months ago  ___ *-1. Don’t know*  ___ *-2. Refused* | ________ Days ago  ________ Weeks ago  ________ Months ago  ___ *-1. Don’t know*  ___ *-2. Refused* |
| J10. Are you still in a sexual relationship with this partner? | ___ 0. No  ___ 1. Yes  ___ *-1. Don’t know*  ___ *-2. Refused* | ___ 0. No  ___ 1. Yes  ___ *-1. Don’t know*  ___ *-2. Refused* | ___ 0. No  ___ 1. Yes  ___ *-1. Don’t know*  ___ *-2. Refused* | ___ 0. No  ___ 1. Yes  ___ *-1. Don’t know*  ___ *-2. Refused* | ___ 0. No  ___ 1. Yes  ___ *-1. Don’t know*  ___ *-2. Refused* |
| J11. Since you first had sex with this partner, how many other people have you had sex with during the sexual relationship? | _______ [# of partners]  ___ *-1. Don’t know*  ___ *-2. Refused* | _______ [# of partners]  ___ *-1. Don’t know*  ___ *-2. Refused* | _______ [# of partners]  ___ *-1. Don’t know*  ___ *-2. Refused* | _______ [# of partners]  ___ *-1. Don’t know*  ___ *-2. Refused* | _______ [# of partners]  ___ *-1. Don’t know*  ___ *-2. Refused* |
| J12. Has this partner been tested for HIV? | ___ 0. No**🡪 J16**  ___ 1. Yes  __*-1.Don’t know***🡪J16**  __ *-2. Refused***🡪 J16** | ___ 0. No**🡪 J16**  ___ 1. Yes  __*-1.Don’t know***🡪J16**  __ *-2. Refused***🡪 J16** | ___ 0. No**🡪 J16**  ___ 1. Yes  __*-1.Don’t know***🡪J16**  __ *-2. Refused***🡪 J16** | ___ 0. No**🡪 J16**  ___ 1. Yes  __*-1.Don’t know***🡪J16**  __ *-2. Refused***🡪 J16** | ___ 0. No**🡪 J16**  ___ 1. Yes  __*-1.Don’t know***🡪J16**  __ *-2. Refused***🡪 J16** |
| J13. How long ago was this partner tested for HIV? | ____ Days ago  ____ Months ago  ____ Years ago  ___ *-1. Don’t know*  ___ *-2. Refused* | ____ Days ago  ____ Months ago  ____ Years ago  ___ *-1. Don’t know*  ___ *-2. Refused* | ____ Days ago  ____ Months ago  ____ Years ago  ___ *-1. Don’t know*  ___ *-2. Refused* | ____ Days ago  ____ Months ago  ____ Years ago  ___ *-1. Don’t know*  ___ *-2. Refused* | ____ Days ago  ____ Months ago  ____ Years ago  ___ *-1. Don’t know*  ___ *-2. Refused* |
| J14. Do you know this partner’s HIV status? | ___ 0. No**🡪 J16**  ___ 1. Yes  __*-1.Don’t know***🡪J16**  __ *-2. Refused***🡪 J16** | ___ 0. No**🡪 J16**  ___ 1. Yes  __*-1.Don’t know***🡪J16**  __ *-2. Refused***🡪 J16** | ___ 0. No**🡪 J16**  ___ 1. Yes  __*-1.Don’t know***🡪J16**  __ *-2. Refused***🡪 J16** | ___ 0. No**🡪 J16**  ___ 1. Yes  __*-1.Don’t know***🡪J16**  __ *-2. Refused***🡪 J16** | ___ 0. No**🡪 J16**  ___ 1. Yes  __*-1.Don’t know***🡪J16**  __ *-2. Refused***🡪 J16** |
| J15. What is this partner’s HIV status? | ___ 0. HIV-Positive**🡪 J18**  ___ 1. HIV-Negative  ___ *-1. Don’t know*  ___ *-2. Refused* | ___ 0. HIV-Positive**🡪 J18**  ___ 1. HIV-Negative  ___ *-1. Don’t know*  ___ *-2. Refused* | ___ 0. HIV-Positive**🡪 J18**  ___ 1. HIV-Negative  ___ *-1. Don’t know*  ___ *-2. Refused* | ___ 0. HIV-Positive**🡪 J18**  ___ 1. HIV-Negative  ___ *-1. Don’t know*  ___ *-2. Refused* | ___ 0. HIV-Positive**🡪 J18**  ___ 1. HIV-Negative  ___ *-1. Don’t know*  ___ *-2. Refused* |
| J16. How confident are you that you could talk with this partner in the next month about getting tested for HIV? | ___ 1. Very confident  ___ 2. Confident  ___ 3. Somewhat  confident  ___ 4. Not at all  confident  ___ *-1. Don’t know*  ___ *-2. Refused* | ___ 1. Very confident  ___ 2. Confident  ___ 3. Somewhat  confident  ___ 4. Not at all  confident  ___ *-1. Don’t know*  ___ *-2. Refused* | ___ 1. Very confident  ___ 2. Confident  ___ 3. Somewhat  confident  ___ 4. Not at all  confident  ___ *-1. Don’t know*  ___ *-2. Refused* | ___ 1. Very confident  ___ 2. Confident  ___ 3. Somewhat  confident  ___ 4. Not at all  confident  ___ *-1. Don’t know*  ___ *-2. Refused* | ___ 1. Very confident  ___ 2. Confident  ___ 3. Somewhat  confident  ___ 4. Not at all  confident  ___ *-1. Don’t know*  ___ *-2. Refused* |
| J17. How worried are you about transmitting HIV to this partner? | ___ 1. Very worried  ___ 2. Somewhat  worried  ___ 3. Not at all  worried  ___ *-1. Don’t know*  ___ *-2. Refused* | ___ 1. Very worried  ___ 2. Somewhat  worried  ___ 3. Not at all  worried  ___ *-1. Don’t know*  ___ *-2. Refused* | ___ 1. Very worried  ___ 2. Somewhat  worried  ___ 3. Not at all  worried  ___ *-1. Don’t know*  ___ *-2. Refused* | ___ 1. Very worried  ___ 2. Somewhat  worried  ___ 3. Not at all  worried  ___ *-1. Don’t know*  ___ *-2. Refused* | ___ 1. Very worried  ___ 2. Somewhat  worried  ___ 3. Not at all  worried  ___ *-1. Don’t know*  ___ *-2. Refused* |
| J18. Have you told this partner that you are HIV-positive? | ___ 0. No **🡪 J21**  ___ 1. Yes  __*-1. Don’t know***🡪J22**  __ *-2. Refused* **🡪 J22** | ___ 0. No **🡪 J21**  ___ 1. Yes  __*-1. Don’t know***🡪J22**  __ *-2. Refused* **🡪 J22** | ___ 0. No **🡪 J21**  ___ 1. Yes  __*-1. Don’t know***🡪J22**  __ *-2. Refused* **🡪 J22** | ___ 0. No **🡪 J21**  ___ 1. Yes  __*-1. Don’t know***🡪J22**  __ *-2. Refused* **🡪 J22** | ___ 0. No **🡪 J21**  ___ 1. Yes  __*-1. Don’t know***🡪J22**  __ *-2. Refused* **🡪 J22** |
| J19. How long ago did you tell this partner that you are HIV-positive? | ____ Days ago  ____ Months ago  ____ Years ago  ___ *-1. Don’t know*  ___ *-2. Refused* | ____ Days ago  ____ Months ago  ____ Years ago  ___ *-1. Don’t know*  ___ *-2. Refused* | ____ Days ago  ____ Months ago  ____ Years ago  ___ *-1. Don’t know*  ___ *-2. Refused* | ____ Days ago  ____ Months ago  ____ Years ago  ___ *-1. Don’t know*  ___ *-2. Refused* | ____ Days ago  ____ Months ago  ____ Years ago  ___ *-1. Don’t know*  ___ *-2. Refused* |
| J20. When you told this partner you are HIV-positive did your partner hit, slap, kick, or do anything else to hurt you physically? | ___ 0. No  ___ 1. Yes  ___ *-1. Don’t know*  ___ *-2. Refused*  **Go to J22** | ___ 0. No  ___ 1. Yes  ___ *-1. Don’t know*  ___ *-2. Refused*  **Go to J22** | ___ 0. No  ___ 1. Yes  ___ *-1. Don’t know*  ___ *-2. Refused*  **Go to J22** | ___ 0. No  ___ 1. Yes  ___ *-1. Don’t know*  ___ *-2. Refused*  **Go to J22** | ___ 0. No  ___ 1. Yes  ___ *-1. Don’t know*  ___ *-2. Refused*  **Go to J22** |
| *If ‘no’ to J18:*  J21. How confident are you that you could tell this partner about your HIV status in the next month? | ___ 1. Very confident  ___ 2. Confident  ___ 3. Somewhat confident  ___ 4. Not at all confident  ___ *-1. Don’t know*  ___ *-2. Refused* | ___ 1. Very confident  ___ 2. Confident  ___ 3. Somewhat confident  ___ 4. Not at all confident  ___ *-1. Don’t know*  ___ *-2. Refused* | ___ 1. Very confident  ___ 2. Confident  ___ 3. Somewhat confident  ___ 4. Not at all confident  ___ *-1. Don’t know*  ___ *-2. Refused* | ___ 1. Very confident  ___ 2. Confident  ___ 3. Somewhat confident  ___ 4. Not at all confident  ___ *-1. Don’t know*  ___ *-2. Refused* | ___ 1. Very confident  ___ 2. Confident  ___ 3. Somewhat confident  ___ 4. Not at all confident  ___ *-1. Don’t know*  ___ *-2. Refused* |
| J22. Does this partner provide any financial support to you or any of your children? | ___ 0. No  ___ 1. Yes  ___ *-1. Don’t know*  ___ *-2. Refused* | ___ 0. No  ___ 1. Yes  ___ *-1. Don’t know*  ___ *-2. Refused* | ___ 0. No  ___ 1. Yes  ___ *-1. Don’t know*  ___ *-2. Refused* | ___ 0. No  ___ 1. Yes  ___ *-1. Don’t know*  ___ *-2. Refused* | ___ 0. No  ___ 1. Yes  ___ *-1. Don’t know*  ___ *-2. Refused* |
| J23.Do you provide any financial support to this partner? | ___ 0. No  ___ 1. Yes  ___ *-1. Don’t know*  ___ *-2. Refused* | ___ 0. No  ___ 1. Yes  ___ *-1. Don’t know*  ___ *-2. Refused* | ___ 0. No  ___ 1. Yes  ___ *-1. Don’t know*  ___ *-2. Refused* | ___ 0. No  ___ 1. Yes  ___ *-1. Don’t know*  ___ *-2. Refused* | ___ 0. No  ___ 1. Yes  ___ *-1. Don’t know*  ___ *-2. Refused* |
| J24. In the past 90 days, how many times did you have vaginal sex with this partner? | ________ [# of times]  **If 0, skip to J29**  ___ *-1. Don’t know*  ___ *-2. Refused* | ________ [# of times]  **If 0, skip to J29**  ___ *-1. Don’t know*  ___ *-2. Refused* | ________ [# of times]  **If 0, skip to J29**  ___ *-1. Don’t know*  ___ *-2. Refused* | ________ [# of times]  **If 0, skip to J29**  ___ *-1. Don’t know*  ___ *-2. Refused* | ________ [# of times]  **If 0, skip to J29**  ___ *-1. Don’t know*  ___ *-2. Refused* |
| J25. How many of these times in the past 90 days did you use a condom during vaginal sex? | ________ [# of times]  ___ *-1. Don’t know*  ___ *-2. Refused* | ________ [# of times]  ___ *-1. Don’t know*  ___ *-2. Refused* | ________ [# of times]  ___ *-1. Don’t know*  ___ *-2. Refused* | ________ [# of times]  ___ *-1. Don’t know*  ___ *-2. Refused* | ________ [# of times]  ___ *-1. Don’t know*  ___ *-2. Refused* |
| J26. Did you or your partner use a condom the last time you had vaginal sex together? | ___ 0. No  ___ 1. Yes  ___ *-1. Don’t know*  ___ *-2. Refused* | ___ 0. No  ___ 1. Yes  ___ *-1. Don’t know*  ___ *-2. Refused* | ___ 0. No  ___ 1. Yes  ___ *-1. Don’t know*  ___ *-2. Refused* | ___ 0. No  ___ 1. Yes  ___ *-1. Don’t know*  ___ *-2. Refused* | ___ 0. No  ___ 1. Yes  ___ *-1. Don’t know*  ___ *-2. Refused* |
| J27. During the past 14 days, how many times did you have vaginal sex with this partner? | ________ [# of times]  **If 0, skip to J29**  ___ *-1. Don’t know*  ___ *-2. Refused* | ________ [# of times]  **If 0, skip to J29**  ___ *-1. Don’t know*  ___ *-2. Refused* | ________ [# of times]  **If 0, skip to J29**  ___ *-1. Don’t know*  ___ *-2. Refused* | ________ [# of times]  **If 0, skip to J29**  ___ *-1. Don’t know*  ___ *-2. Refused* | ________ [# of times]  **If 0, skip to J29**  ___ *-1. Don’t know*  ___ *-2. Refused* |
| J28. How many of these times in the past 14 days did you use a condom during vaginal sex? | ________ [# of times]  ___ *-1. Don’t know*  ___ *-2. Refused* | ________ [# of times]  ___ *-1. Don’t know*  ___ *-2. Refused* | ________ [# of times]  ___ *-1. Don’t know*  ___ *-2. Refused* | ________ [# of times]  ___ *-1. Don’t know*  ___ *-2. Refused* | ________ [# of times]  ___ *-1. Don’t know*  ___ *-2. Refused* |
| J29. In the past 90 days, how many times did you have anal sex with this partner? | ________ [# of times]  **If 0, skip to J6**  ___ *-1. Don’t know*  ___ *-2. Refused* | ________ [# of times]  **If 0, skip to J6**  ___ *-1. Don’t know*  ___ *-2. Refused* | ________ [# of times]  **If 0, skip to J6**  ___ *-1. Don’t know*  ___ *-2. Refused* | ________ [# of times]  **If 0, skip to J6**  ___ *-1. Don’t know*  ___ *-2. Refused* | ________ [# of times]  **If 0, skip to K1**  ___ *-1. Don’t know*  ___ *-2. Refused* |
| J30. How many of these times in the past 90 days did you use a condom during anal sex? | ________ [# of times]  ___ *-1. Don’t know*  ___ *-2. Refused* | ________ [# of times]  ___ *-1. Don’t know*  ___ *-2. Refused* | ________ [# of times]  ___ *-1. Don’t know*  ___ *-2. Refused* | ________ [# of times]  ___ *-1. Don’t know*  ___ *-2. Refused* | ________ [# of times]  ___ *-1. Don’t know*  ___ *-2. Refused* |
| J31. Did you or your partner use a condom the last time you had anal sex together? | ___ 0. No  ___ 1. Yes  ___ *-1. Don’t know*  ___ *-2. Refused* | ___ 0. No  ___ 1. Yes  ___ *-1. Don’t know*  ___ *-2. Refused* | ___ 0. No  ___ 1. Yes  ___ *-1. Don’t know*  ___ *-2. Refused* | ___ 0. No  ___ 1. Yes  ___ *-1. Don’t know*  ___ *-2. Refused* | ___ 0. No  ___ 1. Yes  ___ *-1. Don’t know*  ___ *-2. Refused* |
| J32. During the past 14 days, how many times did you have anal sex with this partner? | ________ [# of times]  **If 0, skip to J6**  ___ *-1. Don’t know*  ___ *-2. Refused* | ________ [# of times]  **If 0, skip to J6**  ___ *-1. Don’t know*  ___ *-2. Refused* | ________ [# of times]  **If 0, skip to J6**  ___ *-1. Don’t know*  ___ *-2. Refused* | ________ [# of times]  **If 0, skip to J6**  ___ *-1. Don’t know*  ___ *-2. Refused* | ________ [# of times]  **If 0, skip to K1**  ___ *-1. Don’t know*  ___ *-2. Refused* |
| J33. How many of these times in the past 14 days did you use a condom during anal sex? | ________ [# of times]  ___ *-1. Don’t know*  ___ *-2. Refused* | ________ [# of times]  ___ *-1. Don’t know*  ___ *-2. Refused* | ________ [# of times]  ___ *-1. Don’t know*  ___ *-2. Refused* | ________ [# of times]  ___ *-1. Don’t know*  ___ *-2. Refused* | ________ [# of times]  ___ *-1. Don’t know*  ___ *-2. Refused* |

**[*REMEMBER: Turn back to J6 on Page 25 and fill out the next partner column if the patient has additional partners.]***

**Sex exchange**

| ***Interviewer reads: “***Now I would like to ask you questions about some other important aspects of a person's life. I know that some of these questions are very personal. However, your answers are crucial for helping to understand the condition of people in [COUNTRY]. Let me assure you that your answers are completely confidential and will not be told to anyone and no one else will know that you were asked these questions. If we should come to any question that you do not want to answer, just let me know and we will go on to the next question.” |
| --- |

|  | **No** | **Yes** | *Don’t know* | *Refused* |
| --- | --- | --- | --- | --- |
| K1. Have you ever had sex in order to get a place to stay? | 0  *Skip to K3* | 1 | *-1* | *-2* |
| K2. In the last 6 months, did you have sex in order to get a place to stay? | 0 | 1 | *-1* | *-2* |
| K3. Have you ever had sex to get money? | 0  *Skip to K5* | 1 | *-1* | *-2* |
| K4. In the last 6 months, did you have sex to get money? | 0 | 1 | *-1* | *-2* |
| K5. Have you ever had sex to get food? | 0  *Skip to K7* | 1 | *-1* | *-2* |
| K6. In the last 6 months, did you have sex to get food? | 0 | 1 | *-1* | *-2* |
| K7. Have you ever had sex to get gifts? | 0  *Skip to L1* | 1 | *-1* | *-2* |
| K8. In the last 6 months, did you have sex to get gifts? | 0 | 1 | *-1* | *-2* |

**Violence**

L1. Have any of your current partners hit, slapped, kicked, or done anything else to hurt you physically?

_____ 0. No 🡺 *Skip to L2*

_____ 1. Yes

*_____ -1. Don’t know*  🡺 *Skip to L2*

*_____ -2. Refused*  🡺 *Skip to L2*

*_____ -3. Not Applicable* 🡺 *Skip to L2*

L1a. Did this happen in the last 6 months?

_____ 0. No

_____ 1. Yes

*_____ -1. Don’t know*

*_____ -2. Refused*

L2. Has anyone else ever hit, slapped, kicked, or done anything else to hurt you physically?

_____ 0. No 🡺 *Skip to L3*

_____ 1. Yes

*_____ -1. Don’t know* 🡺 *Skip to L3*

*_____ -2. Refused* 🡺 *Skip to L3*

L2a. Did this happen in the last 6 months?

_____ 0. No

_____ 1. Yes

*_____ -1. Don’t know*

*_____ -2. Refused*

L3. Has anyone ever forced you to have sexual intercourse or perform any other sexual acts?

_____ 0. No 🡺 *Skip to M1 in next section*

_____ 1. Yes

*_____ -1. Don’t know* 🡺 *Skip to M1 in next section*

*_____ -2. Refused* 🡺 *Skip to M1 in next section*

L3a. Did this happen in the last 6 months?

_____ 0. No

_____ 1. Yes

*_____ -1. Don’t know*

*_____ -2. Refused*

**Sexual Self-Efficacy**

| ***Interviewer reads:*** “The next items are about condom use.   - Please answer all items as they apply to your current situation. - If you have not had experience in the given situation, answer how you might feel about it.” |
| --- |

M1. I feel comfortable asking my partner(s) to use condoms.

_____ 0. No

_____ 1. Yes

*_____ -1. Don’t know*

*_____ -2. Refused*

M2. I know how to use male condoms correctly.

_____ 0. No

_____ 1. Yes

*_____ -1. Don’t know*

*_____ -2. Refused*

M3. I know how to use female condoms correctly.

_____ 0. No

_____ 1. Yes

*_____ -1. Don’t know*

*_____ -2. Refused*

M4. I can get enough male condoms to use them every time I have sex.

_____ 0. No

_____ 1. Yes

*_____ -1. Don’t know*

*_____ -2. Refused*

M5. I can get enough female condoms to use them every time I have sex.

_____ 0. No

_____ 1. Yes

*_____ -1. Don’t know*

*_____ -2. Refused*

M6. I can refuse to have sex with my partner if he/she does not want to use condoms.

_____ 0. No

_____ 1. Yes

*_____ -1. Don’t know*

*_____ -2. Refused*

**Sexually Transmitted Infections (STIs)**

| ***Interviewer reads:*** “These next few questions have to do with any experiences you have had with any infections in your private / genital area.” |
| --- |

N1. Have you ever had any of the following sexually transmitted infection symptoms*? [Read the following choices. Tick all that apply]*

_____ 1. Abnormal or unusual discharge from your vagina **(women only)**

_____ 2. Abnormal or unusual discharge from your penis **(men only)**

_____ 3. Sores in your genital area

_____ 4. Pain in your lower abdomen **(women only)**

*_____-1. Don’t know*

*_____-2. Refused*

*[If NO answer choice is ticked above, skip to P1f]*

N2. Have you ever been treated for a sexually transmitted infection or symptoms such as genital discharge, sores in your genital area, pain during urination, or (for women only) pain in your lower abdomen?

_____ 0. No

_____ 1. Yes

*_____-1. Don’t know*

*_____-2. Refused*

**Discharge**

N3. In the past 6 months, have you had abnormal or unusual discharge from the vagina (women)/penis(men)? Please do not include discharge during sexual intercourse.

_____ 0. No 🡺 *Skip to N7*

_____ 1. Yes

*_____-1. Don’t know* 🡺 *Skip to N7*

*_____-2. Refused* 🡺 *Skip to N7*

N4. Did you receive treatment for the discharge?

_____ 0. No 🡺 *Skip to N6*

_____ 1. Yes

*_____-1. Don’t know*🡺 *Skip to N6*

*_____-2. Refused* 🡺 *Skip to N6*

N5. Where did you receive this treatment? [*Tick all that apply].*

_____ 1. HIV clinic

_____ 2. STI clinic

_____ 3. Out patient day clinic

_____ 4. Pharmacy

_____ 5. Traditional healer not in health care clinics

_____ 6. Other (specify) _______________________

*_____-1. Don’t know*

*_____-2. Refused*

N6. Did your sex partner(s) get treated for discharge?

_____ 0. No

_____ 1. Yes

*_____-1. Don’t know*

*_____-2. Refused*

**Genital sores**

N7. In the past 6 months, have you had any sores in your genital area?

_____ 0. No 🡺 *Skip to N11 if* ***FEMALE*** *or P1m if* ***MALE***

_____ 1. Yes

*_____-1. Don’t know* 🡺 *Skip to N11 if* ***FEMALE*** *or P1m if* ***MALE***

*_____-2. Refused* 🡺 *Skip to N11 if* ***FEMALE*** *or P1m if* ***MALE***

N8. Did you receive treatment for the sores in your genital area?

_____ 0. No 🡺 *Skip to N10*

_____ 1. Yes

*_____-1. Don’t know* 🡺 *Skip to N10*

*_____-2. Refused* 🡺 *Skip to N10*

N9. Where did you receive this treatment? [*Tick all that apply].*

_____ 1. HIV clinic

_____ 2. STI clinic

_____ 3. Out patient day clinic

_____ 4. Pharmacy

_____ 5. Traditional healer not in health care clinics

_____ 6. Other (specify)____________________

*_____-1. Don’t know*

*_____-2. Refused*

N10. Did your sex partner(s) get treated for genital sores?

_____ 0. No

_____ 1. Yes

*_____-1. Don’t know*

*_____-2. Refused*

*Males skip to P1m in next section*

**FOR FEMALES ONLY**

**Lower Abdominal Pain:**

N11. In the past 6 months, have you had pain in your lower abdomen?

_____ 0. No 🡺 *Skip to the beginning of the* **Family Planning** *section*

_____ 1. Yes

*_____-1. Don’t know* 🡺 *Skip to the beginning of the* **Family Planning** *section*

*_____-2. Refused* 🡺 *Skip to the beginning of the* **Family Planning** *section*

N12. Did your health care provider treat this lower abdominal pain as a sexually transmitted infection?

_____ 0. No 🡺 *Skip to the beginning of the* **Family Planning** *section*

_____ 1. Yes

*_____-1. Don’t know* 🡺 *Skip to the beginning of the* **Family Planning** *section*

*_____-2. Refused* 🡺 *Skip to the beginning of the* **Family Planning** *section*

N13. Did you receive treatment for this lower abdominal pain?

_____ 0. No 🡺 *Skip to N15*

_____ 1. Yes

*_____-1. Don’t know* 🡺 *Skip to N15*

*_____-2. Refused* 🡺 *Skip to N15*

N14. Where did you receive this treatment? [*Tick all that apply].*

_____ 1. HIV clinic

_____ 2. STI clinic

_____ 3. Out patient day clinic

_____ 4. Pharmacy

_____ 5. Traditional healer not in health care clinics

_____ 6. Other (specify) ____________________

*_____-1. Don’t know*

*_____-2. Refused*

N15. Did your sex partner(s) get treated for a sexually transmitted infection?

_____ 0. No

_____ 1. Yes

*_____-1. Don’t know*

*_____-2. Refused*

**Family Planning**

| ***Interviewer reads:*** *“*Now I am going to ask you some questions about pregnancy, family planning, and your desire for children.” |
| --- |

**FOR MALES: GO TO QUESTION P1m ON PAGE 38**

**FOR FEMALES ONLY**

P1f. Has anyone in this HIV clinic ever spoken with you about contraceptives or family planning?

_____ 0. No🡺 *Skip to P2f*

_____ 1. Yes

*_____-1. Don’t know*

*_____-2. Refused*

P1fa. Which of the following staff at this HIV clinic have ever discussed contraceptives or family planning with you?

_____ 1. Health Care Provider (e.g, Clinical Officer, Doctor)

_____ 2. Nurse

_____ 3. Counselor

_____ 4. Pharmacist

_____ 5. Other (specify) ____________________

*_____-1. Don’t know*

*_____-2. Refused*

P2f. Are you pregnant now?

_____ 0. No

_____ 1. Yes 🡺 *Skip to P11f*

*_____-1. Don’t know*

*_____-2. Refused*

P3f. Do you want to get pregnant in the next 6 months?

_____ 0. No

_____ 1. Yes

*_____-1. Don’t know*

*_____-2. Refused*

P4f. Does your spouse or main sex partner want you to get pregnant in the next 6 months?

_____ 0. No

_____ 1. Yes

_____ 2. Don’t have a spouse or main sex partner

*_____-1. Don’t know*

*_____-2. Refused*

P5f. Have you ever used any method to avoid getting pregnant?

_____ 0. No

_____ 1. Yes

*_____-1. Don’t know*

*_____-2. Refused*

P6f. Are you currently using any of these methods to avoid getting pregnant?

|  | **No** | **Yes** | *Don’t know* | *Refused* |
| --- | --- | --- | --- | --- |
| a. Pill | 0 | 1 | -1 | -2 |
| b. IUD (Intra-Uterine Device) | 0 | 1 | -1 | -2 |
| c. Injectable | 0 | 1 | -1 | -2 |
| d. Implants | 0 | 1 | -1 | -2 |
| e. Condom | 0 | 1 | -1 | -2 |
| f. Diaphragm | 0 | 1 | -1 | -2 |
| g. Foam/jelly | 0 | 1 | -1 | -2 |
| h. Lactational amenorrhea | 0 | 1 | -1 | -2 |
| i. Rhythm Method | 0 | 1 | -1 | -2 |
| j. Withdrawl | 0 | 1 | -1 | -2 |
| k. Female Sterilization | 0 | 1 | -1 | -2 |
| l. Male Sterilization | 0 | 1 | -1 | -2 |
| m. Other (Specify) ______________________ | 0 | 1 | -1 | -2 |

🡪 **If NO to ALL of above, then ask P7f. Otherwise Skip** 🡪**P8f.**

P7f. I am going to read you some reasons people sometimes give for not using any method to avoid getting pregnant. For each of these, please tell me if this is a reason why you are not using any method.

|  | **No** | **Yes** | *Don’t know* | *Refused* |
| --- | --- | --- | --- | --- |
| a. I want to have children | 0 | 1 | -1 | -2 |
| b. My partner or I cannot get pregnant | 0 | 1 | -1 | -2 |
| c. I do not like the side effects of contraceptives | 0 | 1 | -1 | -2 |
| d. Contraceptives are inconvenient | 0 | 1 | -1 | -2 |
| e. I do not like the contraceptive methods available to me | 0 | 1 | -1 | -2 |
| f. Contraceptives are too hard for me to get | 0 | 1 | -1 | -2 |
| g. My religion does not allow me to use contraceptives | 0 | 1 | -1 | -2 |
| h. Contraceptives cost too much | 0 | 1 | -1 | -2 |
| i. My partner opposes using contraceptives | 0 | 1 | -1 | -2 |
| j. Contraceptives have never been offered to me | 0 | 1 | -1 | -2 |
| k. I do not know enough about contraceptives to know what to use | 0 | 1 | -1 | -2 |
| l. I just have not thought about using contraceptives | 0 | 1 | -1 | -2 |
| m. Other (Specify) _____________________________ | 0 | 1 | -1 | -2 |

*[ After answering, Skip to P13f]*

**Write up to three method(s) from P6f:**

**Do not include female sterilization or male sterilization.**

|  | Method 1  ___ *-1. Don’t know*  ___ *-2. Refuse* | Method 2  ___ *-1. Don’t know*  ___ *-2. Refuse* | Method 3  ___ *-1. Don’t know*  ___ *-2. Refuse* |
| --- | --- | --- | --- |
| P8f. The last time you got *[insert method here]* who prescribed it to you or gave it to you?  SKIP THIS QUESTION IF METHOD IS LACTATIONAL AMENORRHEA, RHYTHM METHOD, OR WITHDRAWL | ___ 1. Someone in this  clinic  ___ 2. Someone in another  part of this health  facility  ___ 3. A provider at  another health  facility  ___ 4. Got method without  a prescription  ___ 5. Other source  (specify)  ________________  ___ *-1. Don’t know*  ___ *-2. Refused* | ___ 1. Someone in this  clinic  ___ 2. Someone in another  part of this health  facility  ___ 3. A provider at  another health  facility  ___ 4. Got method without  a prescription  ___ 5. Other source  (specify)  ________________  ___ *-1. Don’t know*  ___ *-2. Refused* | ___ 1. Someone in this  clinic  ___ 2. Someone in another  part of this health  facility  ___ 3. A provider at  another health  facility  ___ 4. Got method without  a prescription  ___ 5. Other source  (specify)  ________________  ___ *-1. Don’t know*  ___ *-2. Refused* |
| P9f. For how long have you been using *[insert method here]* now without stopping? | ________ [# of months]  ________ [# of years]  ___ *-1. Don’t know*  ___ *-2. Refused* | ________ [# of months]  ________ [# of years]  ___ *-1. Don’t know*  ___ *-2. Refused* | ________ [# of months]  ________ [# of years]  ___ *-1. Don’t know*  ___ *-2. Refused* |
| P10f. Does your spouse or main sex partner know that you are using this method of contraception? | _____ 0. No  _____ 1. Yes  _____ 2. Don’t have a spouse or main sex partner  ___ *-1. Don’t know*  ___ *-2. Refused* | _____ 0. No  _____ 1. Yes  _____ 2. Don’t have a spouse or main sex partner  ___ *-1. Don’t know*  ___ *-2. Refused* | _____ 0. No  _____ 1. Yes  _____ 2. Don’t have a spouse or main sex partner  ___ *-1. Don’t know*  ___ *-2. Refused* |

*[After answering, skip to P13f]*

P11f. How many months pregnant are you?

__________ # months pregnant

*_____-1. Don’t know*

*_____-2. Refused*

P12f. At the time you became pregnant, did you want to become pregnant *then*, did you want to wait until *later*, or did you *not want* to have any (more) children?

_____ 1. Then

_____ 2. Later

_____ 3. Not at all

*_____-1. Don’t know*

*_____-2. Refused*

P13f. In your relationship with your spouse or partner, who decides if or when you should have more children? *[Tick one.]*

_____ 1. I do

_____ 2. My partner does

_____ 3. We decide together

_____ 4. My extended family does

_____ 5. No one really decides, we just let whatever is going to

happen, happen

_____ 6. No opinion

_____ 7. Other (Specify) _____________________

*_____-1. Don’t know*

*_____-2. Refused*

P14f. In the past month, have you talked with your spouse or main partner about contraceptives or planning your family?

_____ 0. No

_____ 1. Yes

*_____-1. Don’t know*

*_____-2. Refused*

P15f. How confident are you that you could talk with your spouse or main partner in the next month about contraceptives or planning your family?

_____ 1. Very confident

_____ 2. Confident

_____ 3. Somewhat confident

_____ 4. Not at all confident

*_____-1. Don’t know*

*_____-2. Refused*

*[Skip items for men and go to Q1 in next section]*

**FOR MALES ONLY**

| ***Interviewer reads:*** “Now I am going to ask you questions about pregnancy, family planning, and your desire for children with your main partner.” |
| --- |

P1m. Has anyone in this HIV clinic ever spoken with you about contraceptives or family planning?

_____ 0. No🡺 *Skip to P2m*

_____ 1. Yes

*_____-1. Don’t know*

*_____-2. Refused*

P1ma. Which of the following staff at this HIV clinic have ever discussed contraceptives or family planning with you?

_____ 1. Health Care Provider (e.g, Clinical Officer, Doctor)

_____ 2. Nurse

_____ 3. Counselor

_____ 4. Pharmacist

_____ 5. Other (specify) ____________________

*_____-1. Don’t know*

*_____-2. Refused*

P2m. Is the person you consider to be your spouse or main partner currently pregnant?

_____ 0. No

_____ 1. Yes 🡺 *Skip to P8m*

_____ 2. No main partner 🡺 *Skip to Q1 in next section*

*_____-1. Don’t know*

*_____-2. Refused*

P3m. Do you want her to get pregnant in the next 6 months?

_____ 0. No

_____ 1. Yes

*_____-1. Don’t know*

*_____-2. Refused*

P4m. Does your spouse or main partner want to get pregnant in the next 6 months?

_____ 0. No

_____ 1. Yes

*_____-1. Don’t know*

*_____-2. Refused*

P5m. Have you ever used any method to avoid getting your spouse or partner(s) pregnant?

_____ 0. No 🡺 *Skip to P7m*

_____ 1. Yes

*_____-1. Don’t know*

*_____-2. Refused*

P6m. Are you, your spouse, or your main sex partner currently using any of these methods to avoid getting pregnant?

|  | **No** | **Yes** | *Don’t know* | *Refused* |
| --- | --- | --- | --- | --- |
| a. Pill | 0 | 1 | *-1* | *-2* |
| b. IUD (Intra-Uterine Device) | 0 | 1 | *-1* | *-2* |
| c. Injectable | 0 | 1 | *-1* | *-2* |
| d. Implants | 0 | 1 | *-1* | *-2* |
| e. Condom | 0 | 1 | *-1* | *-2* |
| f. Diaphragm | 0 | 1 | *-1* | *-2* |
| g. Foam/jelly | 0 | 1 | *-1* | *-2* |
| h. Lactational amenorrhea | 0 | 1 | *-1* | *-2* |
| i. Rhythm Method | 0 | 1 | *-1* | *-2* |
| j. Withdrawl | 0 | 1 | *-1* | *-2* |
| k. Female Sterilization | 0 | 1 | *-1* | *-2* |
| l. Male Sterilization | 0 | 1 | *-1* | *-2* |
| m. Other (Specify) ______________________ | 0 | 1 | *-1* | *-2* |

🡪 **If NO to ALL of above, then ask P7m. Otherwise Skip** 🡪**P10m.**

P7m. I am going to read you some reasons people sometimes give for not using any method to avoid getting pregnant. For each of these, please tell me if this is a reason why you and your partner are not using any method.

|  | **No** | **Yes** | ***Don’t know*** | ***Refused*** |
| --- | --- | --- | --- | --- |
| a. I want to have children | 0 | 1 | *-1* | *-2* |
| b. My partner or I cannot get pregnant | 0 | 1 | *-1* | *-2* |
| c. I do not like using condoms | 0 | 1 | *-1* | *-2* |
| d. My partner does not like using condoms | 0 | 1 | *-1* | *-2* |
| e. My partner does not like the side effects of contraceptives | 0 | 1 | *-1* | *-2* |
| f. Contraceptives are inconvenient | 0 | 1 | *-1* | *-2* |
| g. My partner does not like the contraceptive methods that are available | 0 | 1 | *-1* | *-2* |
| h. Contraceptives are too hard for my partner to get | 0 | 1 | *-1* | *-2* |
| i. My religion does not allow me to use contraceptives | 0 | 1 | *-1* | *-2* |
| j. Contraceptives cost too much | 0 | 1 | *-1* | *-2* |
| k. My partner opposes using contraceptives | 0 | 1 | *-1* | *-2* |
| l. Contraceptives have never been offered to my partner | 0 | 1 | *-1* | *-2* |
| m. I do not know enough about contraceptives to know what to use or for my partner to use | 0 | 1 | *-1* | *-2* |
| n. I just have not thought about using contraceptives | 0 | 1 | *-1* | *-2* |
| o. Other (Specify)  ________________________________  ________________________________ | 0 | 1 | *-1* | *-2* |

***AFTER ANSWERING P7m, SKIP TO P10m.***

P8m. How many months pregnant is your spouse or main partner now?

__________ # months pregnant

*_____-1. Don’t know*

*_____-2. Refused*

P9m. At the time your spouse or main partner became pregnant, did you want her to become pregnant *then*, did you want to wait until *later*, or did you *not want* to have any (more) children?

_____ 1. Then

_____ 2. Later

_____ 3. Not at all

*_____-1. Don’t know*

*_____-2. Refused*

P10m. In your relationship with your spouse or main partner, who decides if or when you should have more children? *[Tick one.]*

_____ 1. I do

_____ 2. My partner does

_____ 3. We decide together

_____ 4. My extended family does

_____ 5. No one really decides, we just let whatever is going to

happen, happen

_____ 6. No opinion

_____ 7. Other (Specify) ________________________

*_____-1. Don’t know*

*_____-2. Refused*

P11m. Have you talked with your spouse or main partner about contraceptives or planning your family in the past month?

_____ 0. No

_____ 1. Yes

*_____-1. Don’t know*

*_____-2. Refused*

P12m. How confident are you that you could talk with your spouse or main partner in the next month about contraceptives or planning your family?

_____ 1. Very confident

_____ 2. Confident

_____ 3. Somewhat confident

_____ 4. Not at all confident

*_____-1. Don’t know*

*_____-2. Refused*

**Prevention Services Received**

| ***Interviewer reads:*** “Now I am going to ask you questions about your experience with your visits to this HIV clinic during the past 6 months.” |
| --- |

**Health care provider**

Q1. In the past 6 months, on average about how long did you meet with the health care provider each time?

__________ minutes

*_____-1. Don’t know*

*_____-2. Refused*

Q2. During your visits over the past 6 months did you feel like you had enough time to discuss your health or other concerns with the health care provider?

_____ 0. No

_____ 1. Yes

*_____-1. Don’t know*

*_____-2. Refused*

Q3. When was the last time you met with a health care provider in this clinic?

*[If participant met with health care provider today, tick box below, otherwise fill in how long it has been.]*

⁮ Today

_____ 1. days ago

_____ 2. weeks ago

_____ 3. months ago

*_____-1. Don’t know*

*_____-2. Refused*

Q4. I am going to read you some topics your health care provider might have talked to you about during your visits in the past 6 months. Please tell me if your health care provider did or did not talk to you about these during your visits in the past 6 months.

*[****Interviewer:*** *Read each response option to participant. Read the “yes” and “no” answer choices aloud to the participant. Don’t read the “don’t remember” and “refused” answer choices aloud to the participant.]*

|  | **No** | **Yes** | *Don’t remember* | *Refused* |
| --- | --- | --- | --- | --- |
| a. Your health and any problems you are having | 0 | 1 | *-1* | *-2* |
| b. Taking ARV medications as prescribed *[only for those on ARVs]* | 0 | 1 | *-1* | *-2* |
| c. Whether you are (your partner is) pregnant or plan to get pregnant | 0 | 1 | *-1* | *-2* |
| d. Whether you were experiencing any genital discharge, genital ulcers, *[Females only]* or lower abdominal pain. | 0 | 1 | *-1* | *-2* |
| e. Counseling on family planning | 0 | 1 | *-1* | *-2* |
| f. Counseling on safer pregnancy | 0 | 1 | *-1* | *-2* |
| g. Disclosing HIV status to your spouse or partner(s) | 0 | 1 | *-1* | *-2* |
| h. Getting your sex partner(s) tested for HIV | 0 | 1 | *-1* | *-2* |
| i. Using condoms correctly and consistently | 0 | 1 | *-1* | *-2* |
| j. Reducing your number of sex partners | 0 | 1 | *-1* | *-2* |
| k. Reducing your alcohol use | 0 | 1 | *-1* | *-2* |
| l. Healthy/positive living | 0 | 1 | *-1* | *-2* |
| m. Setting an HIV prevention goal | 0 | 1 | *-1* | *-2* |
| n. Are there any other things you talked about that I did not mention?  ***If yes****:* Specify  _____________________________  _____________________________ | 0 | 1 | *-1* | *-2* |

Q5. How helpful has getting information on these topics from the health care providers been for you?

_____ 1. Very helpful

_____ 2. Somewhat helpful

_____ 3. Not helpful

*_____-1. Don’t know*

*_____-2. Refused*

Q6. I am going to read you a list of topics you may have discussed with your health care providers. For each one, please tell me whether you made any changes in what you did or what you discussed with your partner(s) after talking about this with your health care providers?

|  | **No** | **Yes** | *Don’t remember* | *Refused* |
| --- | --- | --- | --- | --- |
| a. Took ARV medications as prescribed *[only for those on ARVs]* | 0 | 1 | -1 | -2 |
| b. Disclosed HIV status to spouse or partner(s) | 0 | 1 | -1 | -2 |
| c. Got sex partner(s) tested for HIV | 0 | 1 | -1 | -2 |
| d. Used condoms correctly and consistently | 0 | 1 | -1 | -2 |
| e. Reduced number of sex partners | 0 | 1 | -1 | -2 |
| f. Addressed family planning issues (contraceptives or safer pregnancy) | 0 | 1 | -1 | -2 |
| g. Reduced alcohol use | 0 | 1 | -1 | -2 |
| h. Healthy/positive living | 0 | 1 | -1 | -2 |
| i. Anything else? Specify:  _________________________________ | 0 | 1 | -1 | -2 |

Q7. Some topics can be difficult to talk about, such as sexual behavior, condom use, and alcohol use. How comfortable do you feel discussing these topics with your health care provider?

_____ 1. Very comfortable

_____ 2. Comfortable

_____ 3. Neither comfortable nor uncomfortable

_____ 4. Uncomfortable

_____ 5. Very uncomfortable

*_____-1. Don’t know*

*_____-2. Refused*

Q8. During your visits with health care providers in the past 6 months, were you given condoms?

_____ 0. No 🡺 *Skip to Q8b*

_____ 1. Yes

*_____-1. Don’t know*

*_____-2. Refused*

Q8a. How many condoms were you given during your last visit?

__________ # condoms

*_____-1. Don’t know*

*_____-2. Refused*

Q8b. In the past 6 months, were condoms available for you to take in this clinic?

_____ 0. No

_____ 1. Yes

*_____-1. Don’t know*

*_____-2. Refused*

Q9. During your visits with health care providers in the past 6 months, did any health care provider refer you to…

|  | **No** | **Yes** | ***Don’t remember*** | ***Refused*** |
| --- | --- | --- | --- | --- |
| a. a counselor? | 0 | 1 | *-1* | *-2* |
| b. a family planning clinic different from this clinic? | 0 | 1 | *-1* | *-2* |
| c. a STI clinic different from this clinic? | 0 | 1 | *-1* | *-2* |
| d. any other clinic or service?  ***If yes****:* Specify  _____________________________________________  _____________________________________________ | 0 | 1 | *-1* | *-2* |

**Counselor**

| ***Interviewer reads:*** “Now I am going to ask you some questions about meeting with counselors at this clinic in the past 6 months. These questions are similar to the questions I just asked you, but instead of asking you about health care providers like doctors, medical officers, nurses, and pharmacists, I will be asking you only about your meetings with counselors.” |
| --- |

R1. In the past 6 months, how many times did you meet with a counselor when you came to this clinic?

__________ # times met with counselor 🡺 *If 0, skip to R1a*

🡺 *If 1, skip to R2*

🡺 *If 2 or more, skip to R1b*

*_____-1. Don’t know*

*_____-2. Refused*

R1a. Would it have been helpful to see a counselor?

_____ 0. No 🡺 *Skip to* **END**

_____ 1. Yes 🡺 *Skip to* **END**

*_____-1. Don’t know* 🡺 *Skip to* **END**

*_____-2. Refused* 🡺 *Skip to* **END**

R1b. Did you meet with the same counselor each time?

_____ 0. No

_____ 1. Yes

*_____-1. Don’t know*

*_____-2. Refused*

R2. Did you want to see a counselor more often?

_____ 0. No 🡺 *Skip to R3*

_____ 1. Yes

*_____-1. Don’t know*

*_____-2. Refused*

R2a.What kept you from seeing a counselor more often?

*[Read all options and tick all that apply.]*

_____ 1. My schedule was too busy

_____ 2. The counselor’s schedule was too busy

_____ 3. Not able to come to the clinic

_____ 4. I did not have anything to discuss with the

counselor

_____ 5. I did not know it was an option to meet more often

_____ 6. Other: Specify ___________

*_____ -1. Don’t know*

*_____ -2. Refused*

R3. During your visits over the past 6 months with the counselor(s), on average, about how long did you meet with the counselor each time?

__________ minutes

*_____-1. Don’t know*

*_____-2. Refused*

R4. During your visit over the past 6 months, did you feel like you had enough time to discuss your health or other concerns with the counselor(s)?

_____ 0. No

_____ 1. Yes

*_____-1. Don’t know*

*_____-2. Refused*

R5. When was the last time you met with a counselor in this clinic?

*[If participant met with counselor today, tick box below, otherwise fill in how long it has been.]*

⁮ Today

_____ 1. days ago

_____ 2. weeks ago

_____ 3. months ago

*_____-1. Don’t know*

*_____-2. Refused*

R6. I am going to read you some topics your counselor(s) might have talked with you about during your visits in the past 6 months. Please tell me if your counselor(s) did or did not talk to you about these during your visits in the past 6 months.

[***Interviewer:*** *Read each response option to participant.]*

|  | **No** | **Yes** | ***Don’t remember*** | ***Refused*** |
| --- | --- | --- | --- | --- |
| a. Your health and any problems you are having now | 0 | 1 | *-1* | *-2* |
| b. Preparing for antiretroviral therapy *[only for those* ***not*** *on ART]* | 0 | 1 | *-1* | *-2* |
| c. Taking ARV medications as prescribed *[only for those on ARVs]* | 0 | 1 | *-1* | *-2* |
| d. Disclosing your HIV status to your spouse or partner(s) | 0 | 1 | *-1* | *-2* |
| e. Getting your sex partner(s) tested for HIV | 0 | 1 | *-1* | *-2* |
| f. Using condoms correctly and consistently | 0 | 1 | *-1* | *-2* |
| g. Reducing your number of sex partners | 0 | 1 | *-1* | *-2* |
| h. Reducing your alcohol use | 0 | 1 | *-1* | *-2* |
| i. Healthy/positive living | 0 | 1 | *-1* | *-2* |
| j. Coping with HIV | 0 | 1 | *-1* | *-2* |
| k. Setting an HIV prevention goal | 0 | 1 | *-1* | *-2* |
| l. Are there any other things you talked about that I did not mention?  ***If yes****:* Specify _____________________________ __________________________ | 0 | 1 | *-1* | *-2* |
| m. Is there anything else you talked about?  ***If yes****:* Specify ____________________________ _____________________________ | 0 | 1 | *-1* | *-2* |

R7. How helpful has getting information on these topics from the counselors been for you?

_____ 1. Very helpful

_____ 2. Somewhat helpful

_____ 3. Not helpful

*_____-1. Don’t know*

*_____-2. Refused*

R8. I am going to read you a list of topics you may have discussed with the counselor. For each one, please tell me whether you made any changes in what you did or what you discussed with your partner(s) after talking about this with the counselor?

|  | **No** | **Yes** | *Don’t remember* | *Refused* |
| --- | --- | --- | --- | --- |
| a. Took ARV medications as prescribed *[only for those on ARVs]* | 0 | 1 | *-1* | *-2* |
| b. Disclosed HIV status to spouse or partner(s) | 0 | 1 | *-1* | *-2* |
| c. Got sex partner(s) tested for HIV | 0 | 1 | *-1* | *-2* |
| d. Used condoms correctly and consistently | 0 | 1 | *-1* | *-2* |
| e. Reduced number of sex partners | 0 | 1 | *-1* | *-2* |
| f. Addressed family planning issues (contraceptives or safer pregnancy) | 0 | 1 | *-1* | *-2* |
| g. Reduced alcohol use | 0 | 1 | *-1* | *-2* |
| h. Healthy/positive living | 0 | 1 | *-1* | *-2* |
| i. Anything else? Specify: _____________________________________ | 0 | 1 | *-1* | *-2* |

R9. Some topics can be difficult to talk about, such as sexual behavior, condom use, and alcohol use. How comfortable do you feel discussing these topics with the counselors?

_____ 1. Very comfortable

_____ 2. Comfortable

_____ 3. Neither comfortable nor uncomfortable

_____ 4. Uncomfortable

_____ 5. Very uncomfortable

*_____-1. Don’t know*

*_____-2. Refused*

R10. During your visits with a counselor(s) in the past 6 months, were you given condoms?

_____ 0. No 🡺 *Skip to* **END**

_____ 1. Yes

*_____-1. Don’t know*

*_____-2. Refused*

R10a. How many condoms were you given the last visit?

__________ # condoms

*_____-1. Don’t know*

*_____-2. Refused*

| ***Interviewer reads:* “**Thank you for taking the time to participate in this interview. We will need to make a follow-up appointment for you in six months for you. Do you have any questions or did you want to add anything else?” |
| --- |

**Comments on Interview:**

*Interviewer, please add any comments regarding this participant or problems with interview, etc.*

**Appendix I**

**Health Care Provider Clinical Care Survey**

Instructions: Health care providers vary in the topics they discuss and services they offer HIV-positive patients in HIV clinics during routine appointments. Please answer each of the questions below about the patients you see on a typical day and topics you discuss or services you regularly provide patients when they see you in the HIV clinic. There are no right or wrong answers.

Health Care Provider ID #: __________

Date: ( _____ **/** _____ **/** __________)

dd mm yyyy

Assessment Number:

_____ 1. Baseline assessment

_____ 2. 6-month assessment

_____ 3. 12-month assessment

1. About how many HIV-positive patients do you see each day in this HIV clinic?

_____ # patients

2. At this clinic, on average, how many minutes do you spend with each HIV-positive patient… ***[If you don’t spend any time with a certain type of HIV-positive patient, enter a “0” for that answer choice.]***

|  | **Number of minutes** |
| --- | --- |
| a. who is receiving HIV antiretroviral medications? |  |
| b. who is receiving care but not HIV antiretroviral medications? |  |
| c. the first time you see a patient at this clinic? |  |

3. There are many important issues in HIV care. Given the large number of patients, health care providers may not be able to discuss all issues during regular clinic visits. Please select how often you typically discuss the following topics with your patients during regular clinic visits.

|  | **Never** | **Rarely** | **Some-times** | **Most of the time** | **Always** |
| --- | --- | --- | --- | --- | --- |
| a. Medication adherence | 1 | 2 | 3 | 4 | 5 |
| b. Avoiding infections such as malaria | 1 | 2 | 3 | 4 | 5 |
| c. Nutritional needs | 1 | 2 | 3 | 4 | 5 |
| d. Appropriate level of physical activity | 1 | 2 | 3 | 4 | 5 |
| e. Avoiding harmful or ineffective expensive treatments | 1 | 2 | 3 | 4 | 5 |
| f. Community support groups | 1 | 2 | 3 | 4 | 5 |
| g. Alcohol consumption (frequency and amount) | 1 | 2 | 3 | 4 | 5 |
| h. Impact of alcohol on medication adherence and/or high risk behavior | 1 | 2 | 3 | 4 | 5 |
| i. Pregnancy plans: Intentions to get pregnant or avoid pregnancy | 1 | 2 | 3 | 4 | 5 |
| j. Contraception | 1 | 2 | 3 | 4 | 5 |
| k. Birth spacing | 1 | 2 | 3 | 4 | 5 |
| l. Safer ways to get pregnant | 1 | 2 | 3 | 4 | 5 |
| m. Disclosing HIV status to partner(s) | 1 | 2 | 3 | 4 | 5 |
| n. Whether partner(s) have been tested | 1 | 2 | 3 | 4 | 5 |
| o. Sexual risk reduction | 1 | 2 | 3 | 4 | 5 |
| p. Discussing STI symptoms | 1 | 2 | 3 | 4 | 5 |
| q. Risks of acquiring STIs | 1 | 2 | 3 | 4 | 5 |
| r. Risks of transmitting HIV to partner(s) | 1 | 2 | 3 | 4 | 5 |
| s. Risks of transmitting HIV to baby | 1 | 2 | 3 | 4 | 5 |
| **Are there other topics you typically discuss with patients? If so, please list below and circle how often you discuss these.** | | | | | |
| t. Topic: ______________________ __________________________ | 1 | 2 | 3 | 4 | 5 |
| u. Topic: _____________________ __________________________ | 1 | 2 | 3 | 4 | 5 |
| v. Topic: _____________________ __________________________ | 1 | 2 | 3 | 4 | 5 |
| x. Topic: _____________________ __________________________ | 1 | 2 | 3 | 4 | 5 |
| **Condoms** | | | | | |
| y. How often do you provide condoms to your patients? | 1 | 2 | 3 | 4 | 5 |

5. For each of the topics below, please circle whether you provide this service to your patients at this HIV clinic, refer to another clinic (such as Family Planning or OPD), or a community program for this service, or neither.

|  | **Provide at this clinic** | **Refer to another clinic or program** | **Neither** |
| --- | --- | --- | --- |
| **Family Planning** | | | |
| a. Provide contraceptive pills | 1 | 2 | 3 |
| b. Provide contraceptive injectables | 1 | 2 | 3 |
| c. Insert IUDs | 1 | 2 | 3 |
| d. Conduct sterilizations | 1 | 2 | 3 |
| **STIs** | | | |
| e. Diagnose STI syndromes | 1 | 2 | 3 |
| f. Treat STIs | 1 | 2 | 3 |
| g. Ask patients with STIs to bring their partner(s) in for assessment and treatment | 1 | 2 | 3 |
| h. Encourage bringing partner(s) in for STI treatment | 1 | 2 | 3 |
| **Other topics** | | | |
| i. Offer HIV testing to partner(s) of HIV-positive patients | 1 | 2 | 3 |
| j. Provide support groups for people living with HIV/AIDS | 1 | 2 | 3 |
| k. Provide peer educators for people living with HIV/AIDS | 1 | 2 | 3 |
| l. Provide outreach support (for example, home-based care, home visits or other outreach) for people living with HIV/AIDS | 1 | 2 | 3 |

6. Have any new programs or services been added to your clinic in the last 6 months?

_____ 0. No

_____ 1. Yes

*_____-1. Don’t know*

*_____-2 Refused*

If yes:

Please briefly describe these new programs or services:

_______________________________________________________________

_______________________________________________________________

_______________________________________________________________

_______________________________________________________________

**Appendix J**

**Integration of HIV Prevention into Care and Treatment:**

**Health Care Provider**

Instructions: Health care providers vary in the topics they discuss and services they offer HIV-positive patients in HIV clinics during routine appointments. Please answer each of the questions below in a way that most accurately describes what you do in general at the HIV clinic or with your HIV-positive patients. We also are trying to understand how feasible it is to do HIV prevention activities during routine visits and how acceptable these activities are to you and your patients. There are no right or wrong answers.

Health Care Provider ID #: __________ *[Interviewer: Fill this in before giving to health care provider.]*

Date: ( _____ **/** _____ **/** __________ )

dd mm yyyy

Assessment Number:

_____ 1. Baseline assessment

_____ 2. 6-month assessment

_____ 3. 12-month assessment

1. What is your gender?

_____ 1. Male

_____ 2. Female

2. In what month and year were you born?

(  **____ / _______** )

mm yyyy

***_____*** *-1. Don’t know*

*_____ -2. Refused*

3. How old were you on your last birthday?

__________ Age in completed years

4. What is the highest level of school you attended?

_____ 1. Primary

_____ 2. Secondary

_____ 3. Higher

5. What is the highest (standard/form/year) you *completed* at that level?

__________ Standard/form/year

6. What best describes what type of health care provider you are?

_____ 1. Doctor

_____ 2. Physician

_____ 3. Clinical officer

_____ 4. Nurse

_____ 5. Other (specify)_____________________________

7. How long have you been working as this type of health care professional? *[If < 1 month, code as “1” for months]*

_____1. Months

_____2. Years

8. Do you work full time or part time in this clinic?

_____ 1. Full time

_____ 2. Part time

9. How long have you been working at this clinic? *[If < 1 month, code as “1” for months]*

_____1. Months

_____2. Years

10. How often are you able to meet with patients in a private space in the clinic?

_____ 1. Always

_____ 2. Most of the time

_____ 3. Sometimes

_____ 4. Rarely

_____ 5. Never

11. For each of the items below, please select how much you agree or disagree that it is feasible to do these activities with HIV-positive patients during routine clinic visits.

|  | **Strongly disagree** | **Disagree** | **Neither agree nor disagree** | **Agree** | **Strongly agree** |
| --- | --- | --- | --- | --- | --- |
| a. Discuss HIV testing of partner(s) | 1 | 2 | 3 | 4 | 5 |
| b. Discuss disclosure of HIV status to partner(s) | 1 | 2 | 3 | 4 | 5 |
| c. Discuss sexual risk reduction | 1 | 2 | 3 | 4 | 5 |
| d. Assess patients for alcohol use | 1 | 2 | 3 | 4 | 5 |
| e. Assess patient adherence to ARVs (if taking ARVs) | 1 | 2 | 3 | 4 | 5 |
| f. Assess patients for signs and symptoms of STIs | 1 | 2 | 3 | 4 | 5 |
| g. Treat STIs | 1 | 2 | 3 | 4 | 5 |
| h. Assess patient’s pregnancy status | 1 | 2 | 3 | 4 | 5 |
| i. Assess patient’s pregnancy intentions | 1 | 2 | 3 | 4 | 5 |
| j. Provide basic contraceptives | 1 | 2 | 3 | 4 | 5 |
| k. Give patients condoms at every visit | 1 | 2 | 3 | 4 | 5 |
| l. Overall, how much do you agree or disagree that it is feasible to do these activities during routine clinic visits? | 1 | 2 | 3 | 4 | 5 |

12. For each of the items below, please select how much you agree or disagree that it is important for you to do these activities with HIV-positive patients during routine clinic visits.

|  | **Strongly disagree** | **Disagree** | **Neither agree nor disagree** | **Agree** | **Strongly agree** |
| --- | --- | --- | --- | --- | --- |
| a. Discuss HIV testing of partner(s) | 1 | 2 | 3 | 4 | 5 |
| b. Discuss disclosure of HIV status to partner(s) | 1 | 2 | 3 | 4 | 5 |
| c. Discuss sexual risk reduction | 1 | 2 | 3 | 4 | 5 |
| d. Assess patients for alcohol use | 1 | 2 | 3 | 4 | 5 |
| e. Assess patient adherence to ARVs (if taking ARVs) | 1 | 2 | 3 | 4 | 5 |
| f. Assess patients for signs and symptoms of STIs | 1 | 2 | 3 | 4 | 5 |
| g. Treat STIs | 1 | 2 | 3 | 4 | 5 |
| h. Assess patient’s pregnancy status | 1 | 2 | 3 | 4 | 5 |
| i. Assess patient’s pregnancy intentions | 1 | 2 | 3 | 4 | 5 |
| j. Provide basic contraceptives | 1 | 2 | 3 | 4 | 5 |
| k. Give patients condoms at every visit | 1 | 2 | 3 | 4 | 5 |
| l. Overall, how much do you agree or disagree that it is important for you to discuss HIV prevention during routine clinic visits? | 1 | 2 | 3 | 4 | 5 |

13. For each of the items below, please select how much you agree or disagree that your patients were comfortable receiving these messages or services from health care providers during routine clinic visits.

|  | **Strongly disagree** | **Disagree** | **Neither agree nor disagree** | **Agree** | **Strongly agree** |
| --- | --- | --- | --- | --- | --- |
| a. HIV testing of partner(s) | 1 | 2 | 3 | 4 | 5 |
| b. Disclosure of HIV status to partner(s) | 1 | 2 | 3 | 4 | 5 |
| c. Sexual risk reduction | 1 | 2 | 3 | 4 | 5 |
| d. Alcohol use | 1 | 2 | 3 | 4 | 5 |
| e. Assess patient adherence to ARVs (if taking ARVs) | 1 | 2 | 3 | 4 | 5 |
| f. Assess patients for signs and symptoms of STIs | 1 | 2 | 3 | 4 | 5 |
| g. Treating STIs | 1 | 2 | 3 | 4 | 5 |
| h. Assess patient’s pregnancy status | 1 | 2 | 3 | 4 | 5 |
| i. Assess patient’s pregnancy intentions | 1 | 2 | 3 | 4 | 5 |
| j. Providing basic contraceptives | 1 | 2 | 3 | 4 | 5 |
| k. Providing patients condoms at every visit | 1 | 2 | 3 | 4 | 5 |
| l. Overall, how much do you agree or disagree that patients were comfortable receiving these messages or services during routine clinic visits? | 1 | 2 | 3 | 4 | 5 |

14. How often do you use these tools or job aids to help you to discuss HIV prevention topics with your HIV-positive patients during routine clinic visits?

|  | **Never** | **Rarely** | **Some-times** | **Most of the time** | **Always** |
| --- | --- | --- | --- | --- | --- |
| a. 5 Steps one page job aid | 1 | 2 | 3 | 4 | 5 |
| b. Patient handouts | 1 | 2 | 3 | 4 | 5 |
| c. Posters | 1 | 2 | 3 | 4 | 5 |
| d. HIV prevention messages flipchart | 1 | 2 | 3 | 4 | 5 |
| e. Family planning flipchart | 1 | 2 | 3 | 4 | 5 |
| f. Other (Specify) __________________________ __________________________ | 1 | 2 | 3 | 4 | 5 |

15. Some of the HIV prevention topics can be difficult to talk about, like sexual behavior. How comfortable do you feel discussing these topics with patients?

_____ 1. Very comfortable

_____ 2. Comfortable

_____ 3. Neither comfortable nor uncomfortable

_____ 4. Uncomfortable

_____ 5. Very uncomfortable

16. How has conducting the HIV prevention activities with your HIV-positive patients affected your daily schedule?

_____ 1. I feel busier than before I was doing the intervention with my patients

_____ 2. I feel about as busy as before

_____ 3. I feel less busy than before

17. Compared to 6 months ago, do you see more, the same number, or fewer patients?

_____ 1. I see more patients

_____ 2. I see about the same number of patients

_____ 3. I see fewer patients

18. What kind of impact do you think that conducting the HIV prevention activities has had on your relationship with your patients?

_____ 1. Positive impact

_____ 2. Some positive and some negative impact

_____ 3. Negative impact

_____ 4. No impact

19. What kind of impact do you think that conducting the HIV prevention activities has had on the care you provide your HIV-positive patients?

_____ 1. Positive impact

_____ 2. Some positive and some negative impact

_____ 3. Negative impact

_____ 4. No impact

20. What kind of impact do you think that conducting the HIV prevention activities has had on the health of your HIV-positive patients?

_____ 1. Positive impact

_____ 2. Some positive and some negative impact

_____ 3. Negative impact

_____ 4. No impact

21. What kind of impact do you think that conducting the HIV prevention activities has had on the HIV risk behaviors of your HIV-positive patients?

_____ 1. Positive impact

_____ 2. Some positive and some negative impact

_____ 3. Negative impact

_____ 4. No impact

22. Below are different types of staff who might be at an HIV clinic. For each one, please select how much you agree or disagree that it is the responsibility of those staff to discuss HIV prevention with HIV-positive persons at the HIV clinic during routine clinic visits.

|  | **Strongly disagree** | **Disagree** | **Neither agree nor disagree** | **Agree** | **Strongly agree** |
| --- | --- | --- | --- | --- | --- |
| a. Doctors or physicians | 1 | 2 | 3 | 4 | 5 |
| b. Clinical officers | 1 | 2 | 3 | 4 | 5 |
| c. Nurses | 1 | 2 | 3 | 4 | 5 |
| d. Pharmacists | 1 | 2 | 3 | 4 | 5 |
| e. Counselors | 1 | 2 | 3 | 4 | 5 |
| f. Social workers | 1 | 2 | 3 | 4 | 5 |
| g. Other types of staff. Please specify:**_____________** | 1 | 2 | 3 | 4 | 5 |
| h. Other types of staff. Please specify:**____________** | 1 | 2 | 3 | 4 | 5 |
| i. Other types of staff. Please specify:_____________ | 1 | 2 | 3 | 4 | 5 |

**Appendix K**

**Integration of HIV Prevention into Care and Treatment:**

**Lay Counselor**

Instructions: Read each question and select the answer that most accurately describes what you do in general at the HIV clinic or with your HIV-positive patients. There are no right or wrong answers.

Lay Counselor ID #**:** __________

Date: ( _____ **/** _____ **/** __________ )

dd mm yyyy

Assessment Number:

_____ 1. Baseline assessment

_____ 2. 6-month assessment

_____ 3. 12-month assessment

1. What is your gender?

_____ 1. Male

_____ 2. Female

2. In what month and year were you born?

(  **__ / ____** ) (mm/yyyy)

***_____*** *-1. Don’t know*

*_____ -2. Refused*

3. How old were you on your last birthday?

__________ Age in completed years

4. Have you ever attended school*?*

_____ 0. No 🡺 *Skip to 7*

_____ 1. Yes

5. What is the highest level of school you attended?

_____ 1. Primary

_____ 2. Secondary

_____ 3. Higher

6. What is the highest (standard/form/year) you *completed* at that level?

__________ Standard/form/year

7. How long have you been working as a counselor? *[If < 1 month, code as “1” for months]*

_____1. Months

_____2. Years

8. Did you hang the HIV prevention poster in the waiting area?

_____ 0. No

_____ 1. Yes

9. About how many HIV-positive patients do you see each day in this HIV clinic?

_____ # patients

10. On average, how many minutes do you spend with each HIV-positive patient?

_____ # minutes

11. How often are you able to meet with patients in a private space in the clinic?

_____ 1. Always

_____ 2. Most of the time

_____ 3. Sometimes

_____ 4. Rarely

_____ 5. Never

12. When patients came to you, how often did they have a referral card from a health care provider?

_____ 1. Always

_____ 2. Most of the time

_____ 3. Sometimes

_____ 4. Rarely

_____ 5. Never

13. When patients did not have a referral card, how did they get to you for counseling?

|  | **Never** | **Rarely** | **Some-times** | **Most of the time** | **Always** |
| --- | --- | --- | --- | --- | --- |
| a. The health care provider, such as a medical doctor, or nurse, told them to come. | 1 | 2 | 3 | 4 | 5 |
| b. Another mental health provider, such as a social worker or other counselor, told them to come. | 1 | 2 | 3 | 4 | 5 |
| c. The pharmacist sent them to me. | 1 | 2 | 3 | 4 | 5 |
| d. Before or after the group education session, the patient asked to see me for counseling at the clinic. | 1 | 2 | 3 | 4 | 5 |

14. Since the training on the HIV prevention intervention package (or over the last 6 months), how often did you do the activities listed below?

|  | **Never** | **Rarely** | **Some-times** | **Most of the time** | **Always** |
| --- | --- | --- | --- | --- | --- |
| **Individual Counseling Sessions** |  |  |  |  |  |
| a. Discuss disclosure of HIV status to partner(s) | 1 | 2 | 3 | 4 | 5 |
| b. Encourage patients to bring their partner(s) to the clinic for HIV testing. | 1 | 2 | 3 | 4 | 5 |
| c. Give patient partner HIV testing slips | 1 | 2 | 3 | 4 | 5 |
| d. Discuss sexual risk reduction | 1 | 2 | 3 | 4 | 5 |
| e. Assess patients for alcohol use | 1 | 2 | 3 | 4 | 5 |
| f. Counsel on alcohol reduction (if applicable) | 1 | 2 | 3 | 4 | 5 |
| g. Assess patient adherence to ARVs (if taking ARVs) | 1 | 2 | 3 | 4 | 5 |
| h. Assist patients with adherence to ARVs (if applicable) | 1 | 2 | 3 | 4 | 5 |
| i. Discuss getting ready to begin ART (if applicable) | 1 | 2 | 3 | 4 | 5 |
| j. Give patients condoms at every session. | 1 | 2 | 3 | 4 | 5 |
| k. Assist patients in developing a plan to change the behavior identified | 1 | 2 | 3 | 4 | 5 |
| l. Test partner(s) for HIV | 1 | 2 | 3 | 4 | 5 |
| m. Test couples for HIV | 1 | 2 | 3 | 4 | 5 |
| **Group Counseling Sessions**  Conducted group education sessions on: |  |  |  |  |  |
| n. HIV basics | 1 | 2 | 3 | 4 | 5 |
| o. HIV treatment and adherence | 1 | 2 | 3 | 4 | 5 |
| p. Prevention of sexual transmission | 1 | 2 | 3 | 4 | 5 |
| q. Prevention of mother-to-child transmission | 1 | 2 | 3 | 4 | 5 |
| r. Healthy living | 1 | 2 | 3 | 4 | 5 |

15. For each of the items below, please select how much you agree or disagree that it is feasible to do these activities with HIV-positive patients during your sessions with them.

|  | **Strongly disagree** | **Disagree** | **Neither agree nor disagree** | **Agree** | **Strongly agree** |
| --- | --- | --- | --- | --- | --- |
| **Individual Counseling Sessions** |  |  |  |  |  |
| a. Discuss disclosure of HIV status to partner(s) | 1 | 2 | 3 | 4 | 5 |
| b. Encourage patients to bring their partner(s) to the clinic for HIV testing. | 1 | 2 | 3 | 4 | 5 |
| c. Give patient partner HIV testing slips | 1 | 2 | 3 | 4 | 5 |
| d. Discuss sexual risk reduction | 1 | 2 | 3 | 4 | 5 |
| e. Assess patients for alcohol use | 1 | 2 | 3 | 4 | 5 |
| f. Counsel on alcohol reduction (if applicable) | 1 | 2 | 3 | 4 | 5 |
| g. Assess patient adherence to ARVs (if taking ARVs) | 1 | 2 | 3 | 4 | 5 |
| h. Assist patients with adherence to ARVs (if applicable) | 1 | 2 | 3 | 4 | 5 |
| i. Discuss getting ready to begin ART (if applicable) | 1 | 2 | 3 | 4 | 5 |
| j. Give patients condoms at every session | 1 | 2 | 3 | 4 | 5 |
| k. Assist patients in developing a plan to change the behavior identified | 1 | 2 | 3 | 4 | 5 |
| l. Test partner(s) for HIV | 1 | 2 | 3 | 4 | 5 |
| m. Test couples for HIV | 1 | 2 | 3 | 4 | 5 |
| **Group Counseling Sessions**  Conducted group education sessions on: |  |  |  |  |  |
| n. HIV basics | 1 | 2 | 3 | 4 | 5 |
| o. HIV treatment and adherence | 1 | 2 | 3 | 4 | 5 |
| p. Prevention of sexual transmission | 1 | 2 | 3 | 4 | 5 |
| q. Prevention of mother-to-child transmission | 1 | 2 | 3 | 4 | 5 |
| r. Healthy living | 1 | 2 | 3 | 4 | 5 |
| s. Overall, how much do you agree or disagree that it is feasible to do these activities during routine counseling sessions? | 1 | 2 | 3 | 4 | 5 |

16. For each of the items below, please select how much you agree or disagree that it is important for you to do these activities with HIV-positive patients during your sessions with them.

|  | **Strongly disagree** | **Disagree** | **Neither agree nor disagree** | **Agree** | **Strongly agree** |
| --- | --- | --- | --- | --- | --- |
| **Individual Counseling Sessions** |  |  |  |  |  |
| a. Discuss disclosure of HIV status to partner(s) | 1 | 2 | 3 | 4 | 5 |
| b. Encourage patients to bring their partner(s) to the clinic for HIV testing. | 1 | 2 | 3 | 4 | 5 |
| c. Give patient partner HIV testing slips | 1 | 2 | 3 | 4 | 5 |
| d. Discuss sexual risk reduction | 1 | 2 | 3 | 4 | 5 |
| e. Assess patients for alcohol use | 1 | 2 | 3 | 4 | 5 |
| f. Counsel on alcohol reduction (if applicable) | 1 | 2 | 3 | 4 | 5 |
| g. Assess patient adherence to ARVs (if taking ARVs) | 1 | 2 | 3 | 4 | 5 |
| h. Assist patients with adherence to ARVs (if applicable) | 1 | 2 | 3 | 4 | 5 |
| i. Discuss getting ready to begin ART (if applicable) | 1 | 2 | 3 | 4 | 5 |
| j. Give patients condoms at every session | 1 | 2 | 3 | 4 | 5 |
| k. Assist patients in developing a plan to change the behavior identified | 1 | 2 | 3 | 4 | 5 |
| l. Test partner(s) for HIV | 1 | 2 | 3 | 4 | 5 |
| m. Test couples for HIV | 1 | 2 | 3 | 4 | 5 |
| **Group Counseling Sessions**  Conducted group education sessions on: |  |  |  |  |  |
| n. HIV basics | 1 | 2 | 3 | 4 | 5 |
| o. HIV treatment and adherence | 1 | 2 | 3 | 4 | 5 |
| p. Prevention of sexual transmission | 1 | 2 | 3 | 4 | 5 |
| q. Prevention of mother-to-child transmission | 1 | 2 | 3 | 4 | 5 |
| r. Healthy living | 1 | 2 | 3 | 4 | 5 |
| s. Overall, how much do you agree or disagree that it is important for you to do these activities during routine counseling sessions? | 1 | 2 | 3 | 4 | 5 |

17. For each of the items below, please select how much you agree or disagree that your patients were comfortable receiving these messages or services from lay counselors during routine clinic visits.

|  | **Strongly disagree** | **Disagree** | **Neither agree nor disagree** | **Agree** | **Strongly agree** |
| --- | --- | --- | --- | --- | --- |
| **Individual Counseling Sessions** |  |  |  |  |  |
| a. Disclosing HIV status to partner(s) | 1 | 2 | 3 | 4 | 5 |
| b. Encouraging patients to bring their partner(s) to the clinic for HIV testing. | 1 | 2 | 3 | 4 | 5 |
| c. Providing partner HIV testing slips | 1 | 2 | 3 | 4 | 5 |
| d. Sexual risk reduction | 1 | 2 | 3 | 4 | 5 |
| e. Assessing patients for alcohol use | 1 | 2 | 3 | 4 | 5 |
| f. Counseling on alcohol reduction (if applicable) | 1 | 2 | 3 | 4 | 5 |
| g. Assessing patient adherence to ARVs (if taking ARVs) | 1 | 2 | 3 | 4 | 5 |
| h. Assisting with adherence to ARVs (if applicable) | 1 | 2 | 3 | 4 | 5 |
| i. Getting ready to begin ART (if applicable) | 1 | 2 | 3 | 4 | 5 |
| j. Providing condoms at every session | 1 | 2 | 3 | 4 | 5 |
| k. Assisting patients in developing a plan to change the behavior identified | 1 | 2 | 3 | 4 | 5 |
| l. Test partner(s) for HIV | 1 | 2 | 3 | 4 | 5 |
| m. Test couples for HIV | 1 | 2 | 3 | 4 | 5 |
| **Group Counseling Sessions**  Conducted group education sessions on: |  |  |  |  |  |
| n. HIV basics | 1 | 2 | 3 | 4 | 5 |
| o. HIV treatment and adherence | 1 | 2 | 3 | 4 | 5 |
| p. Prevention of sexual transmission | 1 | 2 | 3 | 4 | 5 |
| q. Prevention of mother-to-child transmission | 1 | 2 | 3 | 4 | 5 |
| r. Healthy living | 1 | 2 | 3 | 4 | 5 |
| s. Overall, how much do you agree or disagree that your patients were comfortable receiving these messages or services during routine counseling sessions? | 1 | 2 | 3 | 4 | 5 |

18. Some of the HIV prevention topics can be difficult to talk about, like sexual behavior. How comfortable do you feel discussing these topics with patients?

_____ 1. Very comfortable

_____ 2. Comfortable

_____ 3. Neither comfortable nor uncomfortable

_____ 4. Uncomfortable

_____ 5. Very uncomfortable

19. Compared to 6 months ago, do you see more, the same number, or fewer patients?

_____ 1. I see more patients

_____ 2. I see about the same number of patients

_____ 3. I see fewer patients

20. What kind of impact do you think that conducting the HIV prevention activities has had on the health of the patients you have met within this clinic?

_____ 1. Positive impact

_____ 2. Some positive and some negative impact

_____ 3. Negative impact

_____ 4. No impact

21. What kind of impact do you think that conducting the HIV prevention activities has had on the HIV risk behaviors of the HIV-positive patients you have met within this clinic?

_____ 1. Positive impact

_____ 2. Some positive and some negative impact

_____ 3. Negative impact

_____ 4. No impact

22. Below are different types of staff who might be at an HIV clinic. For each one, please select how much you agree or disagree that it is the responsibility of those staff to discuss HIV prevention with HIV-positive persons at the HIV clinic during routine clinic visits.

|  | **Strongly disagree** | **Disagree** | **Neither agree nor disagree** | **Agree** | **Strongly agree** |
| --- | --- | --- | --- | --- | --- |
| a. Doctors or physicians | 1 | 2 | 3 | 4 | 5 |
| b. Clinical officers | 1 | 2 | 3 | 4 | 5 |
| c. Nurses | 1 | 2 | 3 | 4 | 5 |
| d. Pharmacists | 1 | 2 | 3 | 4 | 5 |
| e. Counselors | 1 | 2 | 3 | 4 | 5 |
| f. Social workers | 1 | 2 | 3 | 4 | 5 |
| g. Other types of staff. Please specify: ________________ | 1 | 2 | 3 | 4 | 5 |
| h. Other types of staff. Please specify: ______________ | 1 | 2 | 3 | 4 | 5 |
| i. Other types of staff. Please specify: ______________ | 1 | 2 | 3 | 4 | 5 |

**Appendix L**

**Lay Counselor Record**

Patient Name ______________________________ CTC Number ____________

Age ____________ Sex ____________

| **Date of Visit**  **(dd/mm/yyyy)** | **Visit Length (minutes)** | **Topics Discussed** | **Next Steps / Patient Goal** | **Counselor’s Name** | **Comments**  (If partner tested note details here–when, where, etc.) | **Date of Next Appointment**  **(dd/mm/yyyy)** |
| --- | --- | --- | --- | --- | --- | --- |
|  |  | **Discussed:**  ⁮ HIV status disclosure to partner(s)  ⁮ partner(s) HIV testing  ⁮ safer sex  ⁮ alcohol use  ⁮ preparing for ART  ⁮ ART adherence  ⁮ **Tested partner for HIV** |  |  |  |  |
|  |  | **Discussed:**  ⁮ HIV status disclosure to partner(s)  ⁮ partner(s) HIV testing  ⁮ safer sex  ⁮ alcohol use  ⁮ preparing for ART  ⁮ ART adherence  ⁮ **Tested partner for HIV** |  |  |  |  |
|  |  | **Discussed:**  ⁮ HIV status disclosure to partner(s)  ⁮ partner(s) HIV testing  ⁮ safer sex  ⁮ alcohol use  ⁮ preparing for ART  ⁮ ART adherence  ⁮ **Tested partner for HIV** |  |  |  |  |

**Appendix M**

**Health Care Provider Observation Form**

Instructions: To be completed while observing a health care provider during a clinic appointment with a patient.

Observer ID #: __________

Health Care Provider ID #: __________

Date of interview: ( _____ **/** _____ **/** __________)

dd mm yyyy

Assessment Number:

_____ 1. Baseline assessment

_____ 2. 6-month assessment

_____ 3. 12-month assessment

Patient session start time**:** __________

Patient session end time**:** __________

Total visit time**:** __________ minutes *[Calculate after visit is over]*

1. Is the HIV prevention poster on the wall in the office?

_____ 0. No

_____ 1. Yes

2. Is the HIV prevention one-page job aid in a place where it can be easily viewed and/or used?

_____ 0. No

_____ 1. Yes

3. What was patient’s primary reason for clinic visit?

_____ 1. Routine scheduled visit

_____ 2. Acute illness. Describe what patient presented to provider**:**

_______________________________________________________________

_______________________________________________________________

_______________________________________________________________

4. If patient had acute illness or something else that did not allow provider to conduct HIV prevention activities, tick box below and do not complete remainder of questionnaire.

_____ 1. HIV prevention not conducted due to acute patient illness.

_____ 2. HIV prevention not conducted due to other reasons.

Specify why: _______________________________________________________________

_______________________________________________________________

_______________________________________________________________

For each of the items below, circle whether the provider discussed the topic with the patient. If provider did not discuss the topic, provide information about why the provider did not discuss.

|  | **No** | **Yes** | ***Not applicable*** |
| --- | --- | --- | --- |
| 5. Assess whether the patient is sexually active | 0 | 1 | *-3* |
| 6. *(if yes)* Discuss partner(s) HIV status | 0 | 1 | *-3* |
| 7. *(if sexually active)* Ask whether partner has (or partners have) been tested for HIV | 0 | 1 | *-3* |
| 8. Assess whether patient has disclosed to sex partner(s) | 0 | 1 | *-3* |
| 9. *(if has not disclosed to partners)* Encourage disclosure to sex partner(s) | 0 | 1 | *-3* |
| 10. Discuss adopting safer sex behaviors | 0 | 1 | *-3* |
| 11. Discuss abstaining from sex | 0 | 1 | *-3* |
| 12. Discuss reducing number of sex partners | 0 | 1 | *-3* |
| 13. Discuss being faithful to one partner | 0 | 1 | *-3* |
| 14. Discuss using condoms *every time* patient has sex | 0 | 1 | *-3* |
| 15. Explain negative consequences of sex without a condom | 0 | 1 | *-3* |
| 16. Assess alcohol use | 0 | 1 | *-3* |
| 17. *(if needed)* Advise on risky alcohol use and refer to counselor | 0 | 1 | *-3* |
| 18. Assess adherence to ARVs | 0 | 1 | *-3* |
| 19. Assess adherence to other medications | 0 | 1 | *-3* |
| 20. *(if needed)* Refer for adherence counseling | 0 | 1 | *-3* |
| 21. Assess for signs/symptoms of STIs by asking STI screening questions | 0 | 1 | *-3* |
| 22. *(if STI symptoms present)* Conduct exam for STIs | 0 | 1 | *-3* |
| 23. *(if STI symptoms present)* Prescribe treatment | 0 | 1 | *-3* |
| 24. *(if STI symptoms present)* Give Contact Slip for partner STI management | 0 | 1 | *-3* |
| 25. Assess patient/partner pregnancy status | 0 | 1 | *-3* |
| 26. Assess patient/partner pregnancy intentions | 0 | 1 | *-3* |
| 27. Offer appropriate family planning counseling and services | 0 | 1 | *-3* |
| 28. *Tick one of the following:*  ⁮ 1. if pregnant, counsel then refer to PMTCT  ⁮ 2. if does not desire pregnancy, discuss and provide contraception if desired by patient  ⁮ 3. if desires pregnancy, then discuss safer conception methods |  |  |  |
| 29. Encourage patient to work on an HIV prevention goal | 0 | 1 | *-3* |
| 30. Give patient HIV prevention handouts | 0 | 1 | *-3* |
| 31. Give patient condoms  If condoms given, how many? ____________ | 0 | 1 | *-3* |
| 32. Refer patient to lay counselor | 0 | 1 | *-3* |
| 33. Refer patient to other services or clinic. Specify: ____________ | 0 | 1 | *-3* |

| 34. Write reasons why health care provider did not discuss HIV prevention activities. |
| --- |

**Appendix N**

**Lay Counselor Observation Form**

Instructions: To be completed while observing a lay counselor during an individual visit with a patient.

Observer ID #: __________

Lay counselor ID #: __________

Date of interview: ( ____ **/** _____ **/ ____**____)

dd mm yyyy

Assessment Number:

_____ 1. Baseline assessment

_____ 2. 6-month assessment

_____ 3. 12-month assessment

Patient session start time**:** __________

Patient session end time**:** __________

Total visit time**:** __________ minutes *[Calculate after visit is over]*

Circle the appropriate answer to each question below:

|  | **No** | **Yes** | ***Not applicable*** |
| --- | --- | --- | --- |
| 1. Is the HIV prevention poster on the wall in the office? | 0 | 1 | *-3* |
| 1. Is the HIV prevention poster on the wall in the waiting area? | 0 | 1 | *-3* |
| 1. Is the HIV prevention job aid in a place where it can be easily viewed and/or used? | 0 | 1 | *-3* |
| 1. Is the session taking place in a private location? | 0 | 1 | *-3* |

For each of the items below, circle whether the counselor discussed the topic with the patient. If provider did not discuss the topic, provide information why the provider did not discuss.

|  | **No** | **Yes** | ***Not applicable*** |
| --- | --- | --- | --- |
| 1. Discuss disclosure of HIV status to partner(s) | 0 | 1 | *-3* |
| 1. Discuss partner’s HIV status | 0 | 1 | *-3* |
| 1. Encourage patients to bring their partner(s) to the clinic for HIV testing. | 0 | 1 | *-3* |
| 1. Give patient partner HIV testing slips | 0 | 1 | *-3* |
| 1. Discuss safer sex behaviors | 0 | 1 | *-3* |
| 1. Discuss abstaining from sex | 0 | 1 | *-3* |
| 1. Discuss reducing number of sex partners | 0 | 1 | *-3* |
| 1. Discuss using condoms consistently and correctly | 0 | 1 | *-3* |
| 1. Inform patients of negative consequences of sex without a condom | 0 | 1 | *-3* |
| 1. Assess patients for alcohol use | 0 | 1 | *-3* |
| 1. Counsel on alcohol reduction (if applicable) | 0 | 1 | *-3* |
| 1. Assess patient adherence to ARVs (if taking ARVs) | 0 | 1 | *-3* |
| 1. Assist patients with adherence to ARVs (if applicable) | 0 | 1 | *-3* |
| 1. Discuss getting ready to begin ART (if applicable) | 0 | 1 | *-3* |
| 1. Give patients condoms | 0 | 1 | *-3* |
| 1. Assist patients in developing a plan to change the behavior identified | 0 | 1 | *-3* |
| 1. Test partner(s) for HIV | 0 | 1 | *-3* |
| 1. Test couples for HIV | 0 | 1 | *-3* |

| 23. Write reasons why lay counselor did not discuss HIV prevention activities. |
| --- |

**Appendix O**

**Clinic Services Form**

| ***This section is completed every 6 months:*** |
| --- |

Instructions: Information on this form will be collected from clinic records or clinic databases in collaboration with the clinic administration or partners connected to clinic. This section should be completed every 6 months.

Clinic ID #**:** _________________

Data collector ID #**:** _________________

Source(s) of information**:** _________________

Date information was collected: ( _____ **/ _____ / ________** )

dd mm yyyy

Assessment Number:

_____ 1. Baseline

_____ 2. 6-month assessment

_____ 3. 12-month assessment

1. How many of each cadre is currently working at this HIV clinic? *[If there are no members of a certain cadre working at the clinic, enter “0” for that answer choice].*

_____1. Doctors

_____2. Clinical Officers

_____3. Nurse

_____4. Nurse assistants

_____5. Pharmacists

_____6. Counselors (not funded by the project)

_____7. Lay Counselors

2. Tick which days of the week this clinic is open and list the hours the HIV clinic is open.

| _____1. Monday  __________Time clinic opens  __________Time clinic closes  _____2. Tuesday  __________Time clinic opens  __________Time clinic closes  _____3. Wednesday  __________Time clinic opens  __________Time clinic closes  _____4. Thursday  __________Time clinic opens  __________Time clinic closes  _____5. Friday  __________Time clinic opens  __________Time clinic closes |
| --- |

3. How many total patients are there currently enrolled at the HIV clinic?

________ # total clinic patients

4. How many HIV-positive patients are currently attending the clinic for care and services but not taking antiretroviral medications?

________ # patients in care only

5. How many HIV-positive patients are currently on antiretroviral treatment?

________ # patients on ARVs

6. How many HIV-positive patients are seen at the clinic each day, on average?

________ # HIV-positive patients seen/day

7. How many HIV-positive patients on antiretroviral treatment have stopped coming to the clinic (defaulted) in the past 6 months?

________ # on ARVs – defaulted

8. How many HIV-positive patients currently attending the clinic for care and services but not taking antiretroviral medications have stopped coming to the clinic in the last 6 months?

________ # not on ARVs who stopped coming to the clinic

9. How many HIV-positive patients on antiretroviral treatment have missed appointments in the past 6 months?

________ # on ARVs missed appointments

10. How many HIV-positive patients currently attending the clinic for care and services but not taking antiretroviral medications have missed appointments in the last 6 months?

________ # not on ARVs who missed appointments

11. In this HIV clinic, how many HIV tests were conducted in the last 6 months?

________ # HIV tests

| ***The section below is completed monthly:*** |
| --- |

Clinic Name: _________________________________

Data collector ID #: _________________________________

Source(s) of information: _________________________________

Date this information was collected:

( _____ / _____ / __________)

dd mm yyyy

Data Collection Month: *[Tick one]*

_____ 1. January (1^st^ – 31^st^)

_____ 2. February (1^st^ – 28^th^)

_____ 3. March (1^st^ – 31^st^)

_____ 4. April (1^st^ – 30^th^)

_____ 5. May (1^st^ – 31^st^)

_____ 6. June (1^st^ – 30^th^)

_____ 7. July (1^st^ – 31^st^)

_____ 8. August (1^st^ – 31^st^)

_____ 9. September (1^st^ – 30^th^)

_____10.October (1^st^ – 31^st^)

_____11.November (1^st^ – 30^th^)

_____12.December (1^st^ – 31^st^)

1. Number of prescriptions or packets of contraceptive pills given to HIV-positive women enrolled at the HIV clinic in the past month.

**_____________** Number of Prescriptions

1. Number of prescriptions or packets of contraceptive injections given to HIV-positive women enrolled at the HIV clinic in the past month.

**_____________** Number of contraceptive injections

1. Number of HIV-positive patients in the HIV clinic treated for an STI in the past month.

**_____________** Number of patients

**Appendix P**

**Patient Medical Chart Review Form**

Instructions: Information on this form will be collected from patient’s clinic records including patient care forms completed by the health care providers and the Lay Counselor Record Form.

Clinic ID #**:** _____________

Data collector ID #: _____________

Patient ID #: _____________

Patient Gender: _____ Male

_____ Female

Patient Date of Birth: ( ____ **/ ____ / ______** ) (dd/mm/yyyy)

Date Form Completed: ( ____ **/ ____ / ______** ) (dd/mm/yyyy)

Assessment Number:

_____ 1. Baseline assessment

_____ 2. 6-month assessment

_____ 3. 12-month assessment

1. Date patient began receiving medical care for HIV (include care received at previous clinics if patient is a transfer from another clinic):

( ____ **/ ____ / ______** ) (dd/mm/yyyy)

2. Date diagnosed with HIV:

( ____ **/ ____ / ______** ) (dd/mm/yyyy)

3a. Date of last CD-4 count:

( ____ **/ ____ / ______** ) (dd/mm/yyyy)

3b. What was patient’s last CD-4 count?

_____ CD-4 count

4a. Most recent WHO Clinical Stage of HIV/AIDS:

_____ 1. Stage I

_____ 2. Stage II

_____ 3. Stage III

_____ 4. Stage IV

4b. Date of most recent WHO Clinical Stage?

( ____ **/ ____ / ______** ) (dd/mm/yyyy)

5. Is the patient currently taking HIV antiretroviral medications?

_____ 0. No

_____ 1. Yes

6. Date patient began ARVs

( ____ **/ ____ / ______** ) (dd/mm/yyyy)

7. For the last 6 months, list the patient’s prescribed HIV medications, dose, and number of days prescribed for each medication .

| **Date of visit /**  **date prescribed**  **(dd/mm/yyyy)** | **HIV Medication Name**  (include co-trimoxazole, if prescribed) | **Dose** | **# days prescribed** |
| --- | --- | --- | --- |
|  |  |  |  |
|  |  |  |  |
|  |  |  |  |
|  |  |  |  |
|  |  |  |  |
|  |  |  |  |
|  |  |  |  |
|  |  |  |  |
|  |  |  |  |

**8. Family Planning and Sexually Transmitted Infections over the last 6 months:**

| **Date of Clinic Appointment** | **Date:**  **___________**  (dd/mm/yyyy) | **Date:**  **___________**  (dd/mm/yyyy) | **Date:**  **___________**  (dd/mm/yyyy) | **Date:**  **___________**  (dd/mm/yyyy) | **Date:**  **___________**  (dd/mm/yyyy) | **Date:**  **___________**  (dd/mm/yyyy) | **Date:**  **___________**  (dd/mm/yyyy) | **Date:**  **___________**  (dd/mm/yyyy) | **Date:**  **___________**  (dd/mm/yyyy) |
| --- | --- | --- | --- | --- | --- | --- | --- | --- | --- |
| **Females Only** |  |  |  |  |  |  |  |  |  |
| Pregnant | __ 0. No  __ 1. Yes  __-1. Don’t know | __ 0. No  __ 1. Yes  __-1. Don’t know | __ 0. No  __ 1. Yes  __-1. Don’t know | __ 0. No  __ 1. Yes  __-1. Don’t know | __ 0. No  __ 1. Yes  __-1. Don’t know | __ 0. No  __ 1. Yes  __-1. Don’t know | __ 0. No  __ 1. Yes  __-1. Don’t know | __ 0. No  __ 1. Yes  __-1. Don’t know | __ 0. No  __ 1. Yes  __-1. Don’t know |
| **Pregnant women only:** | |  |  |  |  |  |  |  |  |
| Delivery date (dd/mm/yyyy) | ___________ | ___________ | ___________ | ___________ | ___________ | ___________ | ___________ | ___________ | ___________ |
| Taking Efavirenz | __ 0. No  __ 1. Yes | __ 0. No  __ 1. Yes | __ 0. No  __ 1. Yes | __ 0. No  __ 1. Yes | __ 0. No  __ 1. Yes | __ 0. No  __ 1. Yes | __ 0. No  __ 1. Yes | __ 0. No  __ 1. Yes | __ 0. No  __ 1. Yes |
| Referred to PMTCT | __ 0. No  __ 1. Yes | __ 0. No  __ 1. Yes | __ 0. No  __ 1. Yes | __ 0. No  __ 1. Yes | __ 0. No  __ 1. Yes | __ 0. No  __ 1. Yes | __ 0. No  __ 1. Yes | __ 0. No  __ 1. Yes | __ 0. No  __ 1. Yes |
| **Females and Males (spouse/partner)** | | |  |  |  |  |  |  |  |
| Family planning (FP) methods | __ 0. No  __ 1. Yes  **If yes:**  ⁮ 1. Pill  ⁮ 2. Injectables  ⁮ 3. Implants  ⁮ 4. Condoms  ⁮ 5. IUD  ⁮ 6. Other: ____________ | __ 0. No  __ 1. Yes  **If yes:**  ⁮ 1. Pill  ⁮ 2. Injectables  ⁮ 3. Implants  ⁮ 4. Condoms  ⁮ 5. IUD  ⁮ 6. Other: ____________ | __ 0. No  __ 1. Yes  **If yes:**  ⁮ 1. Pill  ⁮ 2. Injectables  ⁮ 3. Implants  ⁮ 4. Condoms  ⁮ 5. IUD  ⁮ 6. Other: ____________ | __ 0. No  __ 1. Yes  **If yes:**  ⁮ 1. Pill  ⁮ 2. Injectables  ⁮ 3. Implants  ⁮ 4. Condoms  ⁮ 5. IUD  ⁮ 6. Other: ____________ | __ 0. No  __ 1. Yes  **If yes:**  ⁮ 1. Pill  ⁮ 2. Injectables  ⁮ 3. Implants  ⁮ 4. Condoms  ⁮ 5. IUD  ⁮ 6. Other: ____________ | __ 0. No  __ 1. Yes  **If yes:**  ⁮ 1. Pill  ⁮ 2. Injectables  ⁮ 3. Implants  ⁮ 4. Condoms  ⁮ 5. IUD  ⁮ 6. Other: ____________ | __ 0. No  __ 1. Yes  **If yes:**  ⁮ 1. Pill  ⁮ 2. Injectables  ⁮ 3. Implants  ⁮ 4. Condoms  ⁮ 5. IUD  ⁮ 6. Other: ____________ | __ 0. No  __ 1. Yes  **If yes:**  ⁮ 1. Pill  ⁮ 2. Injectables  ⁮ 3. Implants  ⁮ 4. Condoms  ⁮ 5. IUD  ⁮ 6. Other: ____________ | __ 0. No  __ 1. Yes  **If yes:**  ⁮ 1. Pill  ⁮ 2. Injectables  ⁮ 3. Implants  ⁮ 4. Condoms  ⁮ 5. IUD  ⁮ 6. Other: ____________ |
| Who provided FP methods? | ⁮ 1. This clinic  ⁮ 2. FP clinic  ⁮ 3. Other clinic | ⁮ 1. This clinic  ⁮ 2. FP clinic  ⁮ 3. Other clinic | ⁮ 1. This clinic  ⁮ 2. FP clinic  ⁮ 3. Other clinic | ⁮ 1. This clinic  ⁮ 2. FP clinic  ⁮ 3. Other clinic | ⁮ 1. This clinic  ⁮ 2. FP clinic  ⁮ 3. Other clinic | ⁮ 1. This clinic  ⁮ 2. FP clinic  ⁮ 3. Other clinic | ⁮ 1. This clinic  ⁮ 2. FP clinic  ⁮ 3. Other clinic | ⁮ 1. This clinic  ⁮ 2. FP clinic  ⁮ 3. Other clinic | ⁮ 1. This clinic  ⁮ 2. FP clinic  ⁮ 3. Other clinic |
| Discussed safer pregnancy | __ 0. No  __ 1. Yes | __ 0. No  __ 1. Yes | __ 0. No  __ 1. Yes | __ 0. No  __ 1. Yes | __ 0. No  __ 1. Yes | __ 0. No  __ 1. Yes | __ 0. No  __ 1. Yes | __ 0. No  __ 1. Yes | __ 0. No  __ 1. Yes |
| **Females and Males: STI Syndromes** | | |  |  |  |  |  |  |  |
| STI Syndrome Identified | __ 0. No  __ 1. Yes | __ 0. No  __ 1. Yes | __ 0. No  __ 1. Yes | __ 0. No  __ 1. Yes | __ 0. No  __ 1. Yes | __ 0. No  __ 1. Yes | __ 0. No  __ 1. Yes | __ 0. No  __ 1. Yes | __ 0. No  __ 1. Yes |
| **If yes, write which STI(s) on the line to the right:**   - Vaginal discharge (VDS) - Urethral discharge (UDS) - Genital ulcer (GUD) - Pelvic inflammatory disease (PID) - Candidiasis - Bacterial vaginosis | **_____________**  List Syndrome(s) Identified  __ Treated at clinic  __ Referred for treatment  __ Partner treated | **_____________**  List Syndrome(s) Identified  __ Treated at clinic  __ Referred for treatment  __ Partner treated | **_____________**  List Syndrome(s) Identified  __ Treated at clinic  __ Referred for treatment  __ Partner treated | **_____________**  List Syndrome(s) Identified  __ Treated at clinic  __ Referred for treatment  __ Partner treated | **_____________**  List Syndrome(s) Identified  __ Treated at clinic  __ Referred for treatment  __ Partner treated | **_____________**  List Syndrome(s) Identified  __ Treated at clinic  __ Referred for treatment  __ Partner treated | **_____________**  List Syndrome(s) Identified  __ Treated at clinic  __ Referred for treatment  __ Partner treated | **_____________**  List Syndrome(s) Identified  __ Treated at clinic  __ Referred for treatment  __ Partner treated | **_____________**  List Syndrome(s) Identified  __ Treated at clinic  __ Referred for treatment  __ Partner treated |

9. Date of appointments, past 6 months:

| (dd/mm/yyyy) | (dd/mm/yyyy) | (dd/mm/yyyy) | (dd/mm/yyyy) | (dd/mm/yyyy) | (dd/mm/yyyy) |
| --- | --- | --- | --- | --- | --- |
|  |  |  |  |  |  |

| (dd/mm/yyyy) | (dd/mm/yyyy) | (dd/mm/yyyy) | (dd/mm/yyyy) | (dd/mm/yyyy) | (dd/mm/yyyy) |
| --- | --- | --- | --- | --- | --- |
|  |  |  |  |  |  |

10. List the dates HIV medications were dispensed and which medications were picked up during the last 6 months (from pharmacy records, if available).

| **Date ARV medications dispensed**  **(dd/mm/yyyy)** | **Names of ARV medications dispensed** | **# of days dispensed** | **Dose** |
| --- | --- | --- | --- |
|  |  |  |  |
|  |  |  |  |
|  |  |  |  |
|  |  |  |  |
|  |  |  |  |
|  |  |  |  |
|  |  |  |  |
|  |  |  |  |
|  |  |  |  |
|  |  |  |  |

**Lay Counselor Record Form**

| **Date of Visit**  **(dd/mm/yyyy)** | **Visit Length (minutes)** | **Topics Discussed** | **Next Steps / Patient Goal** | **Counselor’s Name** | **Comments**  (If partner tested note details here–when, where, etc.) | **Date of Next Appointment**  **(dd/mm/yyyy)** |
| --- | --- | --- | --- | --- | --- | --- |
|  |  | **Discussed:**  ⁮ HIV status disclosure to partner(s)  ⁮ partner(s) HIV testing  ⁮ safer sex  ⁮ alcohol use  ⁮ preparing for ART  ⁮ ART adherence  ⁮ **Tested partner for HIV** |  |  |  |  |
|  |  | **Discussed:**  ⁮ HIV status disclosure to partner(s)  ⁮ partner(s) HIV testing  ⁮ safer sex  ⁮ alcohol use  ⁮ preparing for ART  ⁮ ART adherence  ⁮ **Tested partner for HIV** |  |  |  |  |
|  |  | **Discussed:**  ⁮ HIV status disclosure to partner(s)  ⁮ partner(s) HIV testing  ⁮ safer sex  ⁮ alcohol use  ⁮ preparing for ART  ⁮ ART adherence  ⁮ **Tested partner for HIV** |  |  |  |  |

**Appendix Q**

**Incident Report Form**

# Incident Report

Please report incidents to HRPO by e-mail within 2 working days of CDC’s awareness, to be followed by a completed form 0.1254 within 2 weeks of CDC’s awareness. Supplementary information may be submitted at any time. If reporting an adverse event, include supplemental information, such as on form 0.1254S. See *HRPO Guide: Managing and Reporting Incidents* for further details on how to complete this form.

## Protocol identifiers

CDC protocol ID:       Protocol version number       version date

Protocol title:

## Key CDC personnel

|  | Name and degrees  (FirstName LastName, Degrees) | User ID | SEV # | CDC NC/division |
| --- | --- | --- | --- | --- |
| Primary contact (required) |  |  |  |  |
| Principal investigator (required) |  |  |  |  |
| Person reporting incident |  |  |  |  |

SEV # is CDC’s Scientific Ethics Verification Number. CDC NC/division is the national center (or equivalent) and division (or equivalent), or coordinating center or office if submitted at that level.

## Characterization of incident

### Reporting category

This report covers the following type(s) of incident (check all that apply)

Unanticipated problem(s) involving risks to subjects or others

Adverse event(s) falling into one of the following categories:

Serious, unexpected, and related or possibly related to the research procedures

Serious, expected, but occurring at a significantly higher frequency or severity than expected

Other unexpected adverse events, regardless of severity, that may alter the analysis of the risk versus potential benefit of the research and warrant consideration of substantive changes in the research protocol or consent process

Other unanticipated problem(s) involving risks to subjects or others

Serious or continuing noncompliance with regulations or IRB requirements (e.g., breach of protocol)

Suspension or termination (for reasons other than expiration) [if termination, also submit 0.1253]

Suspension or termination by an IRB

Suspension or termination by someone other than an IRB (e.g., a sponsor)

Other incidents, including those specified in the protocol or requested by the IRB

Briefly explain choice of category/ies (optional):

y _n_ Has this incident, or one similar to or related to it, been previously reported to the IRB under this protocol?

y _n_ Has any other unrelated incident been previously reported to the IRB under this protocol?

### Timing of occurrence and awareness of incident

|  | Begin date | End date (if range) |
| --- | --- | --- |
| Date(s) that incident(s) occurred |  |  |
| Date(s) that site became aware of incident(s) |  |  |
| Date(s) that CDC became aware of incident(s) |  |  |

Further explanation of timing as needed:

### Site of incident occurrence

If incident occurred at multiple sites, indicate primary site here and other sites in the additional comments field.

Institution name:

Institution location:

OHRP Federalwide assurance number:

Support mechanism:

Support award number:

Support end date:

Name of principal investigator at site of occurrence

Title of protocol at site (if different from CDC title)

Tracking number of protocol at site

### Description of incident

Include study identifiers of affected subjects as appropriate. Describe current status, changes in status, and resolution, as appropriate. If this incident is an adverse event, please also describe seriousness, expectedness, and relationship with study intervention.

This incident changes the harm-benefit profile of the research.

### Actions taken to address incident

Explain all actions taken, including suspension of subject enrollment, suspension of research interventions and interactions in already enrolled subjects, revisions of protocol or consent process, and other actions.

The protocol has been or needs to be revised [also submit form 0.1252].

The consent process or document has been or needs to be revised [also submit form 0.1252].

### Other entities to whom this incident is being reported by investigators

List entities to whom investigators are reporting incident, such as the local IRB, DMC, and US and foreign regulators. Do not include entities to whom others are reporting incident. Indicate briefly whether there is regular monitoring, the kind of expertise in monitoring, and the intensity of ongoing review.

## Additional comments
